# Supplementary material for: Glycan remodeled erythrocytes facilitate antigenic characterization of recent A/H3N2 influenza viruses
Source: Nat Commun. 2021 Sep 14;12:5449. doi: 10.1038/s41467-021-25713-1 (PMC8440751; doi:10.1038/s41467-021-25713-1)
Supplement: Supplementary file 1 — Supplementary Information [file 41467_2021_25713_MOESM1_ESM.pdf]

Supplementary Information

Glycan remodeled erythrocytes facilitate antigenic characterization of recent A/H3N2 influenza viruses

Frederik Broszeit†, Rosanne J. van Beek†, Luca Unione, Theo M. Bestebroer, Digantkumar Chapla, Jeong-Yeh Yang, Kelley W. Moremen, Sander Herfst, Ron A.M. Fouchier, Robert P. de Vries* & Geert-Jan Boons*

This PDF file includes:

Materials and Methods 2
  Synthesis of biantennary N-glycans 2
  Structural studies 10
  NMR spectra....................13
Extended data 24
Figure S1. Controls for glycan microarray....................24
Figure S2. Evolution of A/H3N2 hemagglutination ability 25
Figure S3. Concentration-dependent response of representative A/H3N2 viruses in glycan microarray analysis....................26
Figure S4. Glycomic analysis of N-glycosylation of turkey erythrocytes released by Endo F2...27
Figure S5. Relative cell surface occupation of complex and high mannose glycans on chicken and turkey erythrocytes 28
Figure S6. Stability assay of glyco-engineered erythrocytes 29
Table S1. Focus reduction assay 30
Figure S7. Correlation of hemagglutination inhibition and focus reduction assay 31
Figure S8. Receptor binding specificity of representative H1N1 viruses 32
Table S2. Sequence analysis....................33
Figure S9. MD Simulations....................34
Figure S10. CH-π interactions for Gal-6 residue 35
References 36

Materials and Methods

Synthesis of biantennary N-glycans

Materials and methods

Enzymes were expressed as previously described (B3GnT2 and B4GalT1 in [1], PmST1 M144L P34H in [2]). The reaction mixtures were purified using a size exclusion Biogel (P2) from BioRad in Econo glass columns (0.7 x 30 cm / 1.5 x 30 cm / 1.5 x 50 cm) coupled to a BioFrac fraction collector (BioRad). Carbohydrate-containing fractions were detected by thin layer chromatography and an appropriate staining reagent (15 mL AcOH and 3.5 mL *p*-Anisaldehyde in 350 mL EtOH and 50 mL H_2SO_4). Reagents were purchased from Sigma-Aldrich. Uridine 5'-diphosphogalactose (UDP-Gal), uridine 5'-diphospho-N-acetyl-glucosamine (UDP-GlcNAc) and cytidine-5'-monophospho-N-acetylneuraminic acid (CMP-Neu5Ac) were obtained from Roche Diagnostics [UDP-Gal: Cat# 07703562103; UDP-GlcNAc: Cat# 06369855103; CMP-Neu5Ac: Cat# 05974003103]. Final products were purified by high performance liquid chromatography (HPLC) using an XBridge HILIC column (10 mm (Ø) x 250 mm (l), 5 µm particle size) on a semi-preparative liquid chromatography system from Shimadzu (LC-20AT, SIL-20A, CBM-20A, SPD-20A, FRC-10A). The purification was done using 10 mM NH_4HCOO in 10% H_2O in MeCN (buffer B) and 10 mM NH_4HCOO in 100% H_2O (buffer A). The progress of the reactions was monitored on a liquid chromatography mass spectrometry system (LCMS) from Shimadzu (system controller: SCL10A-VP; HPLC pumps: LC10AD-VP; injector: SIL10AD-VP) using a ZIC HILIC column (ZeQuant, PEEK coated guard HPLC column, 3.5 µm particle size, 20 x 2.1 mm). The LC system was attached to a Bruker Daltonics microTOF-Q mass spectrometer.

Synthesis of N-glycans

Sialylglycopeptide (SGP) was extracted from egg yolk and further enzymatically modified to yield compound **1,** which was used as a starting material for the synthesis [3,4]. The terminal galactose of compound **1** was sialylated with an α2,6 specific sialyltransferase mutant P34H/M144L from *Pasteurella multocida* and CMP-Neu5Ac providing compound **2** [2]. This compound was subsequently extended with N-acetyllactosamine (LacNAc) repeats by using mammalian β1,4-galactosyltransferase 1 (B4GalT1) and β1,3-N-acetylglucosamine transferase (B3GnT2) with their corresponding nucleotide sugars UDP-Gal and UDP-GlcNAc, respectively. As a result, compound **3**, **4** and **6** with one, two and three consecutive LacNAc repeats on the MGAT1 (Mannose-3) branch and a terminal α2,6 linked N-acetylneuraminic acid (Neu5Ac) on the MGAT2 (Mannose-6) branch were obtained. These intermediates were used to synthesize the bisialylated compounds **5** and **7** by sialylating the extended MGAT1 (Mannose-3) branch with the α2,6 specific sialyltransferase mutant from *Pasteurella multocida* and CMP-Neu5Ac. Compounds **9** and **11**, modified with a single terminal Neu5Ac on either branch, were prepared by first quantitatively desialylating the intermediates **4** and **6** in an aqueous solution of acetic acid. Afterwards, sialyltransferase ST6Gal1 and CMP-Neu5Ac were used to install a single terminal Neu5Ac moiety providing the products **9** and **11**.

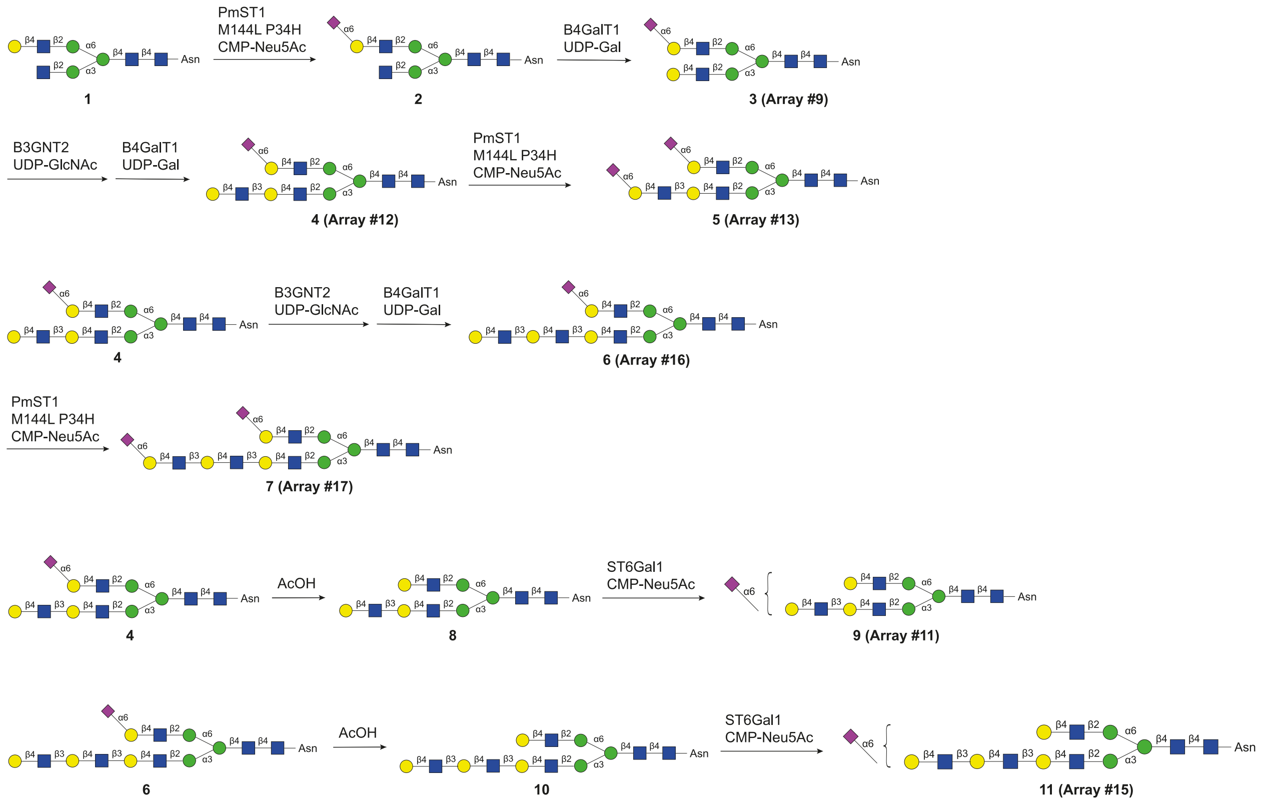

Supplementary Scheme 1. Synthesis of asymmetric, biantennary N-glycans.

General procedure for the installation of α2,6-linked Neu5Ac using PmST1 (P34H/M144L) [2]*:*

The acceptor and CMP-Neu5Ac (2.5 eq) were dissolved in a Tris buffer (100 mM, pH 9, 0.1 wt% BSA) to obtain a concentration of 5 mM. PmST1 M144L P34H (42 µg per µmol acceptor) and CIAP (1 u μL^{-1}, 1 u per µmol of added nucleotide) were added to the reaction mixture and it was incubated overnight at 37 °C with gentle shaking. The progress of the reaction was monitored by LCMS. In case of incomplete conversion after 18 h, additional PmST1 M144L P34H (20 µg per µmol acceptor) was added and the reaction mixture incubated at 37 °C for an additional 24 h. After completion, the reaction mixture was lyophilized and applied to size exclusion chromatography. Carbohydrate-containing fractions were purified by HPLC (**2**: 68%B-66%B in 60 min, 3.3 mL min^{-1}; **5**: 65%B-64%B in 60 min, 3.3 mL min^{-1}; **7**: 65%B-50%B in 100 min, 3.3 mL min^{-1}) providing the product as a white powder (**2**: 16.9 mg, 82%, **5**: 0.48 mg, 17%; **7**: 1.7 mg, 46%).

General procedure for the installation of β1,3-linked glucose using B3GnT2:

The acceptor and UDP-GlcNAc (1.5 eq) were dissolved in a HEPES buffer (50 mM, pH 9.6, 0.1 wt% BSA) containing DTT (1 mM) and $MnCl_2$ (20 mM) to obtain a concentration of 5 mM. B3GnT2 (30 µg per µmol acceptor) and CIAP (1 u μL^{-1}, 1 u per µmol of added nucleotide) were added to the reaction mixture and it was incubated overnight at 37 °C with gentle shaking. The progress of the reaction was monitored by LCMS. In case of incomplete conversion after 18 h, additional UDP-GlcNAc (0.5 eq), CIAP (1 u μL^{-1}, 1 u per µmol of added nucleotide) and B3GnT2 (15 µg per µmol acceptor) were added and the reaction mixture incubated at 37 °C for an additional 24 h. After completion the reaction mixture was lyophilized and applied to size exclusion

chromatography. Carbohydrate-containing fractions were lyophilized and used without further purification.

General procedure for the installation of β1,4-linked galactose using B4GalT1:

The acceptor and UDP-Gal (1.5 eq) were dissolved in a Tris buffer (50 mM, pH 7.3, 0.1wt% BSA) containing $MnCl_2$ (20 mM) to obtain a concentration of 5 mM. B4GalT1 (20 µg per µmol acceptor) and CIAP (1 u μL^{-1}, 1 u per µmol of added nucleotide) were added to the reaction mixture and it was incubated overnight at 37 °C with gentle shaking. The progress of the reaction was monitored by LCMS. In case of incomplete conversion after 18 h, additional UDP-Gal (0.5 eq), CIAP (1 u μL^{-1}, 1 u per µmol of added nucleotide) and B4GalT1 (10 µg per µmol acceptor) were added and the reaction mixture incubated at 37 °C for 24 h. After completion the reaction mixture was lyophilized and applied to size exclusion chromatography. Carbohydrate-containing fractions were purified by HPLC (**3**: 68%B-65%B in 60 min, 3.4 mL min^{-1}; **4**: 65%B for 60 min, 3.4 mL min^{-1}; **6**: 65%B for 60 min, 3.3 mL min^{-1}; **)** providing the products as white powders (**3**: 16.4 mg, 88%; **4**: 19.2 mg, 89%; **6**: 11.3 mg, 75%).

General procedure for the removal terminal Neu5Ac:

The substrate was dissolved in an aqueous solution of acetic acid (2 M) and kept at 65 °C for 24 h. The solvent was removed in an N_2 flow and the reaction mixture was applied to size exclusion chromatography. Carbohydrate-containing fractions were lyophilized and used without further purification.

General procedure for the installation of α2,6-linked Neu5Ac using ST6Gal1:

The acceptor and CMP-Neu5Ac (1.1 eq) were dissolved in a Tris buffer (50 mM, pH 7.3, 0.1 wt% BSA) to obtain a concentration of 2 mM. ST6Gal1 (42 µg per µmol acceptor) was added to the reaction mixture and it was incubated overnight at 37 °C with gentle shaking. The progress of the reaction was monitored by LCMS. In case of incomplete conversion after 18 h, additional ST6Gal1 (20 µg per µmol acceptor) was added and the reaction mixture incubated at 37 °C for 24 h. After completion the reaction mixture was lyophilized and applied to size exclusion chromatography. Carbohydrate-containing fractions were purified by HPLC (**9**: 68%B-64%B in 80 min, 3.4 mL min^{-1}; **11**: 67%B-62%B in 80 min, 3.4 mL min^{-1}) providing the product as a white powder (**9**: 47 µg, 17%, **11**: 50 µg, 10%).

Analytical data

VnmrJ 4 and TopSpin 4 were used to collect NMR data. NMR data was obtained at room temperature on a 600 MHz instrument from Bruker. The chemical shift δ is given in parts per million (ppm) and refers to tetramethylsilane and the residual solvent peak [^{1}H-NMR: $\delta(D_2O)$ = 4.79 ppm]. NMR data is given as follows: ^{1}H-NMR: chemical shift (multiplicity, coupling constants, relative integral, functional group); ^{13}C data are extracted from HSQC spectra and given as follows: chemical shift. Multiplicity is defined as follows: s = singlet; d = doublet; t = triplet; m = multiplet. Signals were assigned by numbering the monosacharide units starting at the reducing end of the oligosaccharide. Monosacharides attached to the mannose-6 branch are indicated by a " ' " (prime) and those attached to the mannose-3 branch without any mark. The assignment was

done by using corresponding 2D-NMR spectra (COSY, HSQC). Due to the use of ammonium formate containing buffers during final purification, several spectra show residual formic acid (8.46 ppm, not shown in the NMR spectra) contamination. The yield/concentration of the final products was determined by NMR spectroscopy, using n-propanol as an internal standard. High resolution masses were measured on an Agilent 6560 Ion Mobility Q-TOF LC-MS system.

2

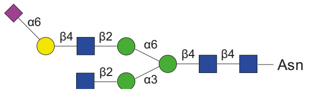

^{1}H NMR (600 MHz, D_2O): δ = 5.13 (s, 1H, H-1, Man-4), 5.08 (d, *J* = 9.8 Hz, 1H, H-1, GlcNAc-1), 4.95 (s, 1H, H-1, Man-4’), 4.79 (s, 1H, H-1, Man-3), 4.64-4.59 (m, 3H, H-1, GlcNAc-5, H-1, GlcNAc-5’), 4.56 (d, *J* = 8.4 Hz, 1H, H-1, GlcNAc-2), 4.46 (d, 1H, H-1, Gal-6’), 4.26 (s, 1H, H-2, Man-3), 4.20 (s, 1H, H-2, Man-4), 4.12 (s, 1H, H-2, Man-4’), 4.03-3.40 (m, 68H, H-2-H-6, GlcNAc-1, H-2-H-6, GlcNAc-2, H-3-H-6, Man-3, H-3-H-6, Man-4, H-3-H-6, Man-4’, H-2-H-6, GlcNAc-5, H-2-H-6, GlcNAc-5’, H-2-H-6, Gal-6’, H-4-H-9, Neu5Ac-7’, αCH-Asn), 2.97-2.84 (m, 2H, βCH_2-Asn), 2.68 (dd, *J* = 12.4 Hz, 4.7 Hz, 1H, H-3eq, Neu5Ac-7’), 2.14-2.10 (m, 19H, NHAc), 1.73 (t, *J* = 12.2 Hz, 1H, H-3ax, NeuAc-7’). ^{13}C NMR from HSQC (150 MHz, D_2O): δ = 103.5, 101.3, 100.5, 99.5, 78.1, 96.9, 99.6, 70.2, 76.4, 76.2, 63.3, 60.4, 50.9, 65.8, 61.1, 69.3, 71.7, 53.6, 60.0, 73.6, 51.8, 65.9, 80.2, 54.8, 60.3, 72.5, 80.7, 78.7, 68.2, 59.8, 61.8, 74.3, 76.2, 68.4, 63.3, 70.7, 67.3, 69.9, 75.8, 35.0, 40.1, 22.3, 40.1. ESI-HRMS: for $C_{71}H_{117}N_7O_{51}$: m/z $[M-2H]^{-2}$; calcd: 940.8316; found: 940.8288.

3 (Array #9)

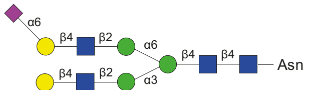

^{1}H NMR (600 MHz, D_2O): δ = 5.13 (s, 1H, H-1, Man-4), 5.08 (d, *J* = 9.8 Hz, 1H, H-1, GlcNAc-1), 4.96 (s, 1H, H-1, Man-4’), 4.78 (s, 1H, H-1, Man-3), 4.64-4.57 (m, 3H, H-1, GlcNAc-2, H-1, GlcNAc-5, H-1, GlcNAc-5’), 4.49-4.44 (m, 2H, H-1, Gal-6, H-1, Gal-6’), 4.26 (s, 1H, H-2, Man-3), 4.20 (s, 1H, H-2, Man-4), 4.12 (s, 1H, H-2, Man-4’), 4.04-3.47 (m, 39H, H-2-H-6, GlcNAc-1, H-2-H-6, GlcNAc-2, H-3-H-6, Man-3, H-3-H-6, Man-4, H-3-H-6, Man-4’, H-2-H-6, GlcNAc-5, H-2-H-6, GlcNAc-5’, H-2-H-6, Gal-6, H-2-H-6, Gal-6’, H-4-H-9, Neu5Ac-7’, αCH-Asn), 2.97-2.84 (m, 2H, βCH_2-Asn), 2.68 (dd, *J* = 12.4 Hz, 4.6 Hz, 1H, H-3eq, Neu5Ac-7’), 2.11-1.99 (m, 17H, NHAc), 1.73 (t, J = 12.2 Hz, 1H, H-3ax, NeuAc-7’). 13C NMR from HSQC (150 MHz, D_2O): δ = 99.5, 78.0, 78.1, 96.9, 100.6, 99.2, 101.3, 99.3, 103.1, 103.3, 70.2, 76.4, 76.2, 63.3, 60.1, 65.9, 51.0, 68.5, 61.7, 69.4, 71.7, 62.6, 53.6, 60.0, 73.8, 51.9, 65.8, 80.4, 60.9, 54.7, 72.3, 78.6, 78.7, 68.2, 61.8, 74.5, 68.3, 63.3, 70.8, 67.3, 35.3, 35.3, 39.9, 30.1, 22.3, 40.0. ESI-HRMS: for $C_{77}H_{127}N_7O_{56}$: m/z $[M-2H]^{-2}$; calcd: 1021.8580; found: 1021.8564.

4 (Array #12)

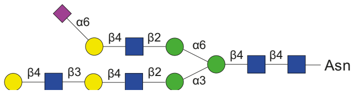

^{1}H NMR (600 MHz, D_2O): δ = 5.12 (s, 1H, H-1, Man-4), 5.08 (d, *J* = 9.6 Hz, 1H, H-1, GlcNAc-1), 4.95 (s, 1H, H-1, Man-4'), 4.78 (s, 1H, H-1, Man-3), 4.71 (d, *J* = 8.4 Hz, 1H, 1-H, GlcNAc-7), 4.64-4.56 (m, 3H, H-1, GlcNAc-2, H-1, GlcNAc-5, H-1, GlcNAc-5'), 4.50-4.44 (m, 3H, H-1, Gal-6, H-1, Gal-6', H-1, Gal-8), 4.26 (s, 1H, H-2, Man-3), 4.19 (s, 1H, H-2, Man-4), 4.16 (s, 2H, H-3, Gal-8), 4.12 (s, 1H, H-2, Man-4'), 4.06-3.46 (m, 43H, H-2-H-6, GlcNAc-1, H-2-H-6, GlcNAc-2, H-3-H-6, Man-3, H-3-H-6, Man-4, H-3-H-6, Man-4', H-2-H-6, GlcNAc-5, H-2-H-6, GlcNAc-5', H-2-H-6, Gal-6, H-2-H-6, Gal-6', H-4-H-9, Neu5Ac-7', H-2-H-6, GlcNAc-7, H-2-H-6, Gal-8, αCH-Asn), 2.98-2.82 (m, 2H, βCH_2-Asn), 2.68 (dd, *J* = 12.9 Hz, 4.3 Hz, 1H, H-3eq, Neu5Ac-7'), 2.12-1.98 (m, 17H, NHAc), 1.73 (t, *J* = 12.2 Hz, 1H, H-3ax, NeuAc-7'). ^{13}C NMR from HSQC (150 MHz, D_2O): δ = 99.5, 78.1, 96.9, 100.5, 102.7, 101.3, 99.4, 103.1, 70.1, 76.4, 68.3, 76.2, 63.3, 50.9, 60.1, 65.8, 68.6, 69.1, 71.8, 62.4, 53.6, 60.0, 73.7, 51.9, 65.8, 54.8, 60.9, 72.3, 78.3, 82.0, 75.0, 80.6, 62.1, 61.7, 74.5, 70.0, 68.4, 63.3, 70.8, 67.3, 35.0, 40.1, 22.2, 40.1. ESI-HRMS: for $C_{91}H_{150}N_8O_{66}$: m/z $[M-2H]^{-2}$; calcd: 1204.4241; found: 1204.4221.

5 (Array #13)

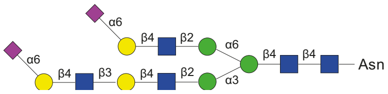

^{1}H NMR (600 MHz, D_2O): δ = 5.10 (s, 1H, H-1, Man-4), 5.06 (d, *J* = 9.8 Hz, 1H, H-1, GlcNAc-1), 4.94 (s, 1H, H-1, Man-4'), 4.76 (s, 1H, H-1, Man-3), 4.72 (d, *J* = 7.7 Hz, 1H, 1-H, GlcNAc-7), 4.62-4.54 (m, 3H, H-1, GlcNAc-2, H-1, GlcNAc-5, H-1, GlcNAc-5'), 4.49-4.41 (m, 3H, H-1, Gal-6, H-1, Gal-6, H-1, Gal-8), 4.24 (s, 1H, H-2, Man-3), 4.18 (d, *J* = 3.7 Hz, 1H, H-2, Man-4), 4.15 (d, *J* = 3.2 Hz, 1H, H-3, Gal-8), 4.11 (d, *J* = 3.4 Hz, 1H, H-2, Man-4'), 4.03-3.44 (m, 44H, H-2-H-6, GlcNAc-1, H-2-H-6, GlcNAc-2, H-3-H-6, Man-3, H-3-H-6, Man-4, H-3-H-6, Man-4', H-2-H-6, GlcNAc-5, H-2-H-6, GlcNAc-5', H-2-H-6, Gal-6, H-2-H-6, Gal-6', H-4-H-9, Neu5Ac-7', H-2-H-6, GlcNAc-7, H-2-H-6, Gal-8, H-4-H-9, Neu5Ac-9, αCH-Asn), 2.96-2.83 (m, 2H, βCH_2-Asn), 2.66 (dd, *J* = 12.6 Hz, 4.5 Hz, 2H, H-3eq, Neu5Ac-9, H-3eq, Neu5Ac-7'), 2.13-1.92 (m, 20H, NHAc), 1.71 (t, *J* = 12.2 Hz, 2H, H-3ax, NeuAc-7', H-3ax, Neu5Ac-9). ^{13}C NMR from HSQC (150 MHz, D_2O): δ = 99.5, 78.1, 96.9, 100.5, 102.6, 101.4, 99.4, 103.3, 70.2, 68.3, 76.2, 63.3, 50.9, 60.1, 65.8, 68.6, 69.3, 71.7, 62.4, 62.6, 53.5, 60.1, 73.6, 51.9, 65.7, 54.7, 60.7, 82.0, 72.4, 80.6, 68.2, 59.7, 62.5, 62.2, 74.4, 68.4, 63.3, 70.7, 67.3, 35.1, 35.0, 40.1, 22.2, 40.1. ESI-HRMS: for $C_{102}H_{167}N_9O_{74}$: m/z $[M-3H]^{-3}$; calcd: 899.9814; found: 899.9787.

6 (Array #16)

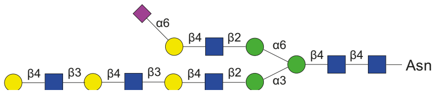

^{1}H NMR (600 MHz, D_2O): δ = 5.13 (s, 1H, H-1, Man-4), 5.08 (d, *J* = 9.8 Hz, 1H, H-1, GlcNAc-1), 4.95 (s, 1H, H-1, Man-4'), 4.78 (s, 1H, H-1, Man-3), 4.71 (d, *J* = 8.5 Hz, 2H, 1-H, GlcNAc-7),

4.65-4.57 (m, 4H, H-1, GlcNAc-2, H-1, GlcNAc-5, H-1, GlcNAc-5', 1-H, GlcNAc-9), 4.50-4.44 (m, 4H, H-1, Gal-6, H-1, Gal-6', H-1, Gal-8, H-1, Gal-10), 4.26 (s, 1H, H-2, Man-3), 4.20 (s, 1H, H-2, Man-4), 4.16 (s, 2H, H-3, Gal-8, H-3, Gal-10), 4.13 (s, 1H, H-2, Man-4'), 4.03-3.48 (m, 53H, H-2-H-6, GlcNAc-1, H-2-H-6, GlcNAc-2, H-3-H-6, Man-3, H-3-H-6, Man-4, H-3-H-6, Man-4', H-2-H-6, GlcNAc-5, H-2-H-6, GlcNAc-5', H-2-H-6, Gal-6, H-2-H-6, Gal-6', H-4-H-9, Neu5Ac-7', H-2-H-6, GlcNAc-7, H-2-H-6, Gal-8, H-2-H-6, GlcNAc-9, H-2-H-6, Gal-10, αCH-Asn), 2.97-2.84 (m, 2H, βCH$_2$-Asn), 2.68 (dd, J = 12.4 Hz, 4.7 Hz, 1H, H-3eq, Neu5Ac-7'), 2.12-2.00 (m, 23H, NHAc), 1.73 (t, J = 12.2 Hz, 1H, H-3ax, Neu5Ac-7'). ^{13}C NMR from HSQC (150 MHz, D$_2$O): δ = 99.5, 78.0, 96.9, 100.5, 102.7, 101.3, 99.3, 102.9, 70.2, 76.4, 68.3, 76.2, 63.3, 50.9, 59.9, 68.5, 61.7, 69.4, 71.7, 62.2, 62.6, 53.5, 59.9, 73.7, 51.9, 55.1, 65.7, 80.4, 80.3, 60.9, 54.7, 72.2, 72.2, 78.2, 82.0, 75.0, 72.4, 80.7, 78.6, 68.2, 59.8, 61.8, 74.5, 78.4, 69.9, 68.4, 63.3, 70.8, 67.3, 35.0, 35.0, 40.0, 22.1, 40.1. ESI-HRMS: for $C_{105}H_{173}N_9O_{76}$: m/z [M-3H]$^{-3}$; calcd: 924.6603; found: 924.6602.

7 (Array #17)

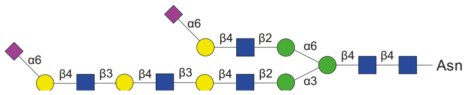

^{1}H NMR (600 MHz, D$_2$O): δ = 5.11 (s, 1H, H-1, Man-4), 5.06 (d, J = 9.8 Hz, 1H, H-1, GlcNAc-1), 4.94 (s, 1H, H-1, Man-4'), 4.77 (s, 1H, H-1, Man-3), 4.74-4.66 (m, 2H, H-1, GlcNAc-7, H-1, GlcNAc-9), 4.63-4.55 (m, 3H, H-1, GlcNAc-2, H-1, GlcNAc-5, H-1, GlcNAc-5'), 4.48-4.42 (m, 4H, H-1, Gal-6, H-1, Gal-6', H-1, Gal-8, H-1, Gal-10), 4.24 (s, 1H, H-2, Man-3), 4.18 (s, 1H, H-2, Man-4), 4.15 (s, 2H, H-3, Gal-6, H-3, Gal-6'), 4.11 (s, 1H, H-2, Man-4'), 4.02-3.47 (m, 45H, H-2-H-6, GlcNAc-1, H-2-H-6, GlcNAc-2, H-3-H-6, Man-3, H-3-H-6, Man-4, H-3-H-6, Man-4', H-2-H-6, GlcNAc-5, H-2-H-6, GlcNAc-5', H-2-H-6, Gal-6, H-2-H-6, Gal-6', H-2-H-6, GlcNAc-7, H-2-H-6, Gal-8, H-2-H-6, GlcNAc-9, H-2-H-6, Gal-10, H-4-H-9, Neu5Ac-11, H-4-H-9, Neu5Ac-7', αCH-Asn), 2.96-2.82 (m, 2H, βCH$_2$-Asn), 2.70-2.63 (m, 2H, H-3eq, Neu5Ac-7', H-3eq, Neu5Ac-11), 2.16-1.98 (m, 22H, NHAc), 1.71 (t, J = 12.1 Hz, 2H, H-3ax, Neu5Ac-7', H-3ax, Neu5Ac-11). ^{13}C NMR from HSQC (150 MHz, D$_2$O): δ = 99.5, 78.0, 96.9, 100.5, 102.6, 101.3, 99.2, 103.3, 70.2, 76.4, 68.3, 76.2, 63.3, 50.9, 60.0, 65.8, 68.4, 61.8, 69.4, 71.7, 62.6, 53.6, 60.0, 73.7, 51.9, 55.0, 65.7, 80.3, 54.7, 60.8, 82.0, 78.3, 74.8, 72.3, 51.9, 80.6, 68.2, 62.5, 74.4, 70.0, 68.4, 63.3, 70.7, 67.3, 34.9, 34.9, 40.1, 22.2, 22.1, 40.1. ESI-HRMS: for $C_{116}H_{190}N_{10}O_{84}$: m/z [M-3H]$^{-3}$; calcd: 1021.6921; found: 1021.6904.

8

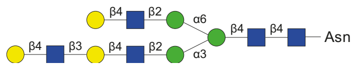

^{1}H NMR (600 MHz, D$_2$O): δ = 5.11 (s, 1H, H-1, Man-4), 5.06 (d, J = 9.8 Hz, 1H, H-1, GlcNAc-1), 4.92 (s, 1H, H-1, Man-4'), 4.77 (s, 1H, H-1, Man-3), 4.69 (d, J = 8.3 Hz, 1H, 1-H, GlcNAc-7), 4.63-4.54 (m, 3H, H-1, GlcNAc-2, H-1, GlcNAc-5, H-1, GlcNAc-5'), 4.49-4.50 (m, 3H, H-1, Gal-6, H-1, Gal-6', H-1, Gal-8), 4.24 (s, 1H, H-2, Man-3), 4.18 (s, 1H, H-2, Man-4), 4.15 (s, 1H, H-3, Gal-8), 4.10 (s, 1H, H-2, Man-4'), 4.01-3.42 (m, 58H, H-2-H-6, GlcNAc-1, H-2-H-6, GlcNAc-2, H-3-H-6, Man-3, H-3-H-6, Man-4, H-3-H-6, Man-4', H-2-H-6, GlcNAc-5, H-2-H-6, GlcNAc-5', H-2-H-6, Gal-6, H-2-H-6, Gal-6', H-2-H-6, GlcNAc-7, H-2-H-6, Gal-8, αCH-Asn), 3.00-2.82 (m,

2H, βCH$_2$-Asn), 2.12-1.96 (m, 17H, NHAc). ^{13}C NMR from HSQC (150 MHz, D$_2$O): δ = 99.5, 78.1, 97.1, 100.4, 100.4, 102.8, 102.8, 101.3, 101.3, 99.5, 102.9, 70.2, 76.4, 68.3, 76.3, 60.0, 65.8, 68.6, 61.7, 61.7, 69.4, 53.6, 59.9, 65.7, 61.0, 54.9, 72.2, 78.5, 75.2, 82.1, 72.5, 59.8, 61.7, 69.9, 74.6, 71.0, 67.3, 35.0, 22.3. ESI-HRMS: for $C_{80}H_{133}N_7O_{58}$: m/z $[M-2H]^{-2}$; calcd: 1058.8764; found: 1058.8761.

9 (Array #11)

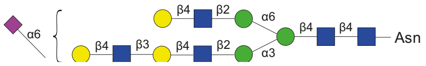

^{1}H NMR (600 MHz, D$_2$O): δ = 5.09 (s, 1H, H-1, Man-4), 5.05 (d, J = 9.9 Hz, 1H, H-1, GlcNAc-1), 4.93 (s, 1H, H-1, Man-4'), 4.75 (s, 1H, H-1, Man-3), 4.67 (d, J = 8.4 Hz, 1H, 1-H, GlcNAc-7), 4.61-4.54 (m, 3H, H-1, GlcNAc-2, H-1, GlcNAc-5, H-1, GlcNAc-5'), 4.47-4.40 (m, 3H, H-1, Gal-6, H-1, Gal-6', H-1, Gal-8), 4.23 (s, 1H, H-2, Man-3), 4.17 (s, 1H, H-2, Man-4), 4.14 (s, 1H, H-3, Gal-8), 4.10 (s, 1H, H-2, Man-4'), 3.99-3.44 (m, 36H, H-2-H-6, GlcNAc-1, H-2-H-6, GlcNAc-2, H-3-H-6, Man-3, H-3-H-6, Man-4, H-3-H-6, Man-4', H-2-H-6, GlcNAc-5, H-2-H-6, GlcNAc-5', H-2-H-6, Gal-6, H-2-H-6, Gal-6', H-2-H-6, GlcNAc-7, H-2-H-6, Gal-8, Neu5Ac-7'/9, αCH-Asn), 2.94-2.81 (m, 2H, βCH$_2$-Asn), 2.65 (d, J = 12.7 Hz, 1H, H-3eq, Neu5Ac-7'/9), 2.08-1.96 (m, 23H, NHAc), 1.70 (t, J = 12.1 Hz, 1H, H-3ax, NeuAc-7'/9). ^{13}C NMR from HSQC (150 MHz, D$_2$O): δ = 99.5, 78.0, 96.9, 100.4, 102.7, 101.2, 98.4, 99.4, 102.8, 102.8, 103.5, 70.1, 76.3, 76.4, 68.3, 68.3, 76.2, 63.2, 50.8, 59.9, 68.4, 61.6, 69.4, 71.7, 71.7, 59.0, 62.6, 59.9, 73.7, 54.9, 80.4, 54.7, 60.9, 72.1, 78.1, 82.0, 69.6, 72.4, 78.0, 80.6, 59.7, 61.6, 70.0, 74.5, 63.3, 70.8, 67.3, 34.9, 40.0, 40.0, 22.3, 22.0, 22.0, 20.0. ESI-HRMS: for $C_{91}H_{150}N_8O_{66}$: m/z $[M-2H]^{-2}$; calcd: 1204.4241; found: 1204.4272.

10

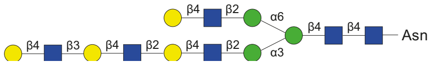

^{1}H NMR (600 MHz, D$_2$O): δ = 5.11 (s, 1H, H-1, Man-4), 5.06 (d, J = 9.7 Hz, 1H, H-1, GlcNAc-1), 4.92 (s, 1H, H-1, Man-4'), 4.76 (s, 1H, H-1, Man-3), 4.69 (2 x d, J = 8.4 Hz, 2H, 1-H, GlcNAc-7, 1-H, GlcNAc-9), 4.64-4.51 (m, 3H, H-1, GlcNAc-2, H-1, GlcNAc-5, H-1, GlcNAc-5', 1-H), 4.47-4.40 (m, 4H, H-1, Gal-6, H-1, Gal-6', H-1, Gal-8, H-1, Gal-10), 4.24 (s, 1H, H-2, Man-3), 4.18 (s, 1H, H-2, Man-4), 4.15 (s, 2H, H-3, Gal-8, H-3, Gal-10), 4.10 (s, 1H, H-2, Man-4'), 4.00-3.44 (m, 51H, H-2-H-6, GlcNAc-1, H-2-H-6, GlcNAc-2, H-3-H-6, Man-3, H-3-H-6, Man-4, H-3-H-6, Man-4', H-2-H-6, GlcNAc-5, H-2-H-6, GlcNAc-5', H-2-H-6, Gal-6, H-2-H-6, Gal-6', H-2-H-6, GlcNAc-7, H-2-H-6, Gal-8, H-2-H-6, GlcNAc-9, H-2-H-6, Gal-10, αCH-Asn), 2.96-2.82 (m, 2H, βCH$_2$-Asn), 2.11-1.97 (m, 17H, NHAc). ^{13}C NMR from HSQC (150 MHz, D$_2$O): δ = 99.5, 78.0, 97.0, 100.4, 102.7, 101.3, 99.4, 102.9, 102.9, 70.2, 76.4, 68.3, 76.3, 50.9, 59.9, 68.5, 61.6, 69.4, 53.6, 59.9, 55.1, 65.7, 60.9, 54.9, 72.2, 78.3, 75.1, 82.0, 72.4, 59.9, 61.6, 70.0, 74.6, 70.9, 67.3, 35.0, 35.0, 22.2, 22.2. ESI-HRMS: for $C_{94}H_{156}N_8O_{68}$: m/z $[M-2H]^{-2}$; calcd: 1241.4425; found: 1241.4440.

11 (Array #15)

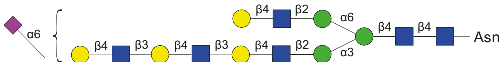

^{1}H NMR (600 MHz, D_2O): δ = 5.09 (s, 1H, H-1, Man-4), 5.05 (d, *J* = 9.6 Hz, 1H, H-1, GlcNAc-1), 4.92 (s, 1H, H-1, Man-4’), 4.76 (s, 1H, H-1, Man-3), 4.68 (d, *J* = 8.3 Hz, 2H, 1-H, GlcNAc-7), 4.61-4.54 (m, 4H, H-1, GlcNAc-2, H-1, GlcNAc-5, H-1, GlcNAc-5’, 1-H, GlcNAc-9), 4.47-4.40 (m, 4H, H-1, Gal-6, H-1, Gal-6’, H-1, Gal-8, H-1, Gal-10), 4.23 (s, 1H, H-2, Man-3), 4.17 (s, 1H, H-2, Man-4), 4.14 (s, 2H, H-3, Gal-8, H-3, Gal-10), 4.09 (s, 1H, H-2, Man-4’), 3.99-3.44 (m, 89H, H-2-H-6, GlcNAc-1, H-2-H-6, GlcNAc-2, H-3-H-6, Man-3, H-3-H-6, Man-4, H-3-H-6, Man-4’, H-2-H-6, GlcNAc-5, H-2-H-6, GlcNAc-5’, H-2-H-6, Gal-6, H-2-H-6, Gal-6’, H-4-H-9, Neu5Ac-7’/11, H-2-H-6, GlcNAc-7, H-2-H-6, Gal-8, H-2-H-6, GlcNAc-9, H-2-H-6, Gal-10, αCH-Asn), 2.95-2.81 (m, 2H, βCH_2-Asn), 2.65 (d, *J* = 12.3 Hz, 1H, H-3eq, Neu5Ac-7’/11), 2.11-1.97 (m, 20H, NHAc), 1.70 (t, *J* = 12.3 Hz, 1H, H-3ax, NeuAc-7’/11). ^{13}C NMR from HSQC (150 MHz, D_2O): δ = 99.5, 78.0, 97.0, 100.4, 102.7, 101.2, 99.4, 102.9, 103.5, 76.3, 68.3, 76.8, 50.8, 59.9, 68.4, 71.1, 53.5, 59.9, 51.8, 55.0, 80.6, 60.9, 78.2, 72.1, 82.0, 78.5, 74.8, 72.4, 68.2, 80.6, 76.2, 74.5, 74.5, 69.9, 68.4, 70.8, 67.3, 35.0, 40.1, 22.1. ESI-HRMS: for $C_{105}H_{173}N_9O_{76}$: m/z [M-2H]$^{-2}$; calcd: 1386.9902; found: 1387.0024.

12 (Array #8)

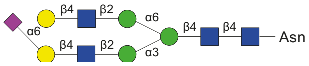

^{1}H NMR (600 MHz, D_2O): δ = 5.12 (s, 1H, H-1, Man-4), 5.06 (d, *J* = 9.7 Hz, 1H, H-1, GlcNAc-1), 4.92 (s, 1H, H-1, Man-4’), 4.76 (s, 1H, H-1, Man-3), 4.63-4.54 (m, 3H, H-1, GlcNAc-2, H-1, GlcNAc-5, H-1, GlcNAc-5’), 4.48-4.42 (m, 2H, H-1, Gal-6, H-1, Gal-6’), 4.25 (s, 1H, H-2, Man-3), 4.19 (s, 1H, H-2, Man-4), 4.10 (s, 1H, H-2, Man-4’), 4.02-3.44 (m, 47H, H-2-H-6, GlcNAc-1, H-2-H-6, GlcNAc-2, H-3-H-6, Man-3, H-3-H-6, Man-4, H-3-H-6, Man-4’, H-2-H-6, GlcNAc-5, H-2-H-6, GlcNAc-5’, H-2-H-6, Gal-6, H-2-H-6, Gal-6’, H-4-H-9, Neu5Ac-7, αCH-Asn), 2.96-2.81 (m, 2H, βCH_2-Asn), 2.66 (dd, *J* = 12.4 Hz, 4.7 Hz, 1H, H-3eq, Neu5Ac-7), 2.11-1.98 (m, 18H, NHAc), 1.71 (t, J = 12.2 Hz, 1H, H-3ax, NeuAc-7). ^{13}C NMR from HSQC (150 MHz, D_2O): δ = 99.5, 78.1, 97.0, 100.3, 101.3, 99.4, 103.1, 70.1, 76.3, 76.3, 60.1, 68.5, 61.6, 71.7, 53.6, 60.0, 73.6, 52.0, 54.7, 60.9, 72.4, 75.2, 78.9, 75.3, 72.4, 68.1, 80.7, 61.8, 74.4, 68.3, 63.4, 70.8, 67.3, 35.0, 40.1, 22.1. ESI-HRMS: for $C_{77}H_{127}N_7O_{56}$: m/z [M-2H]$^{-2}$; calcd: 1021.8580; found: 1021.8575.

Structural studies

It has been found that during the transition from late 90s to early-2000, HA of A/H3N2 viruses have a reduced affinity for the prototypic human receptor, the 6'-SLN [5]. Analysis of protein sequence alignment shows that the main sequences differences reside at the 130-loop (residues 131, 135 and 137), the 150-loop (residues 155-159), the 220-loop (residues 222, 225 and 226), the E190D mutation at the 190-helix, and the appearance of a new glycosylation site at residue 144 and 158. Other residues which contribute to sialic acid binding are highly conserved among HAs including Y98, H183, Y195 and W153 (Table S3).
Although X-ray structural studies [5-8] have provided an understanding of the structural basis of changes in receptor binding specificity, it has not uncovered interactions with extended glycan receptors. Such structural data is difficult to obtain by X-ray crystallography, and therefore, we performing docking experiments of a sialoside having an extended LacNAc moiety with HAs of A/H3N2 viruses representing different evolutionary time points including A/NL/816/91 from 1991 (NL91), A/NL/109/03 from 2003 (NL03) and A/NL/1797/17 from 2017 (NL17).

Modeling of A/NL/816/91 (NL91). NL91 recognized most of the human-type receptors, including compounds that have an α2,6-sialoside on a mono-LacNAc residue (glycans **7-9**, Figure 1A). A somewhat higher responsiveness was observed for receptors having an α2,6-sialoside onto di-LacNAc residue (glycans **10-13**, Figure 1A), while tri-LacNAc containing receptors did not further improve HA binding (glycans **14-17**). Collectively, the results showed that the optimal receptor for NL91 is an N-glycan having two repeating LacNAc moieties modified by a 2,6-linked sialoside (glycan **10** and **13**). Thus, we performed all atoms MD simulations of the complex between the NL91 and the receptor $(LacNAc)_2$α2-6Neu5Ac. The MD simulation showed that the glycan receptor binds the HA protein almost exclusively through the terminal sialic acid. In fact, the analysis of the MD derived trajectory revealed a stable binding pose for the α2,6-sialoside, while the underling di-LacNAc chain explores multiple orientation along the simulation, where only transient intermolecular interactions exist. Comparing X-ray data and the molecular modeling derived structures indicates that the interaction network is preserved in HK68 and NL91. This is consistent with the high structural homology of the sialic binding site of HK68 and NL91 for which only two mutations exist, G135E and N137Y. Actually, these two residues contribute to receptor binding through their backbone atoms (Figure 3A). Specifically, all the hydrophobic interactions, such as those with Y98, H183, Y195 and W153, and H-bond interactions with residues 135-137 and S228 are preserved. In line with the X-ray studies [7], we found that in NL91 the Glu190 engages the Sia-1 O9 through H-bond interaction with an average distance of ~2.9 Å, while L226 makes hydrophobic interactions with the C-6 of the galactose-2 (Figure 3A). For the underling di-LacNAc chain, the MD simulation showed that only the Gal-2 contributes to binding by either engaging E190 or G225 in an H-bond interaction, however, these interactions populate only 10% of the whole MD simulation, which results in a barley defined binding pose for the di-LacNAc chain. The results demonstrate that the HA protein of NL91 recognizes the human receptor through the terminal α2,6-sialoside while the underling glycan does not significantly contribute to binding.

Modeling of A/NL/109/03 (NL03). NL03 recognized far fewer glycans and did not bind to structures having their α2,6-sialosides at a mono-LacNAc moiety (glycans **7-9, 12** and **16** Figure 1A). It recognized structures having the sialoside on at least one di-LacNAc moiety (glycans **10** and **13**), although it shows a significant improvement in responsiveness when interrogated against

structures which contain tri-LacNAc residue (glycans **14**, **15** and **17**). Thus, we performed all atoms MD simulations of the complex between the NL03 and the receptor $(LacNAc)_3\alpha 2$-6NeuAc. The MD simulation showed that the extended LacNAc chain contributes to HA binding. The analysis of the MD derived trajectory revealed a stable binding pose for both the α2,6-sialoside and the underlying tri-LacNAc chain. In agreement with previously reported X-ray studies [7], the analysis of the MD simulations showed that in NL03, the D190 does not participate in sialic acid binding. Instead, the D225 engages the Gal-2 O3 through a H-bond interaction which results in a change in a dihedral angle of the sialic acid-galactose glycosidic bond. The rotation of the Gal-2 residue places all subsequent moieties toward the 190-helix [8]. The MD simulations showed that the Asp190 engages the Gal-4 O2 through H-bond interaction, while the Asn193 provides a H-bond interaction with the acetamide moiety of GlcNAc-3 with an average distance of ~2.5 Å (Figure 3E). Inspection of X-ray crystal structures [7,8] of post 2003 HAs shows that distal mutations to the RBD (A131T, H155T and E156H) reoriented the side chain of Y159, which resulted in an extended receptor binding site (Supplementary Figure 9). The molecular modeling study showed that Gal-6 makes a CH-π interaction with the aromatic ring of Y159 (Figure 3E). The contribution of Y159 in further stabilizing receptor binding was analyzed by monitoring the distance of Gal-6 H3, H4 and H5 against the aromatic ring along the MD simulation (Supplementary Figure 10), which showed a stable interaction. These additional interactions support the hypothesis that post-2000 strains have undergone mutations that compensated for the reduced affinity for the terminal sialoside. Interestingly, the extensive epistatic network which correlates mutations at the RBS with those occurring at distal antigenic site, such as the 150-loop and 190-helix, has been demonstrated by large-scale mutagenesis experiments [7].

Modeling of A/NL/1797/17 (NL17). NL17 and NL19 (3C.2a) showed strong responsiveness only to glycans **14**, **15** and **17** (Figure 1A). These glycans have in common that at least one of the arms is extended by three consecutive LacNAc units that is further modified by an α2,6-sialoside. Thus, a glycan having four LacNAc units arranged in an asymmetrical manner (**15**) represents the minimal receptor for these viruses. Mono-sialylated derivative **15** gave a similar responsiveness compared to the bis-sialosides **14** and **17** indicating that a bidentate binding event does not substantially contribute to recognition as previously suggested [9]. We docked the structure of the NL17 HA protein in complex with the receptor $(LacNAc)_3\alpha 2$-6NeuAc based on the results from MD simulation of NL03 in which we replaced the mutated residues by using the mutagenesis tool implemented in PyMOL. The resulting structure was minimized by using the steepest descent algorithm implemented in the Amber MD program. The orientation of the glycan receptor is very similar in NL03 and NL17. Specifically, NL17 does not present additional amino acids mutation at the sialic acid binding site, leading to the same binding interactions as for NL03 at this site. The Gal-2 is also bound in a similar manner, thus orienting the GlcNAc-3 against the 190-helix. However, in NL17 residue 193 is substituted by Phe for which H-bond interaction is not possible, suggesting that F193 does not contribute to receptor binding (Figure 3G, H and I). The missing interaction with the GlcNAc-3 may be the reason for the lack of binding of di-LacNAc containing glycans observed for NL17 with respect to the reduced binding of NL03 (Figure 1A). Instead, the Y159 is preserved and allows for CH-π interaction with the Gal-6 of the internal LacNAc moiety. The importance of Y159 in extended N-glycans binding is confirmed by the receptor specificity of 3C.3a clade which present the Y159S and F193S mutations. It is expected that these viruses lost the ability to engage the Gal-6 through CH-π interactions while compensate by H-bond interaction between the GlcNAc-3 and the S193, which reflect the re-gained ability of binding sialilated di-

LacNAc glycans (glycans **10** and **13**). The molecular modeling study herein presented support a notion that A/H3N2 viruses have undergone mutations to create and extended binding site.

NMR spectra

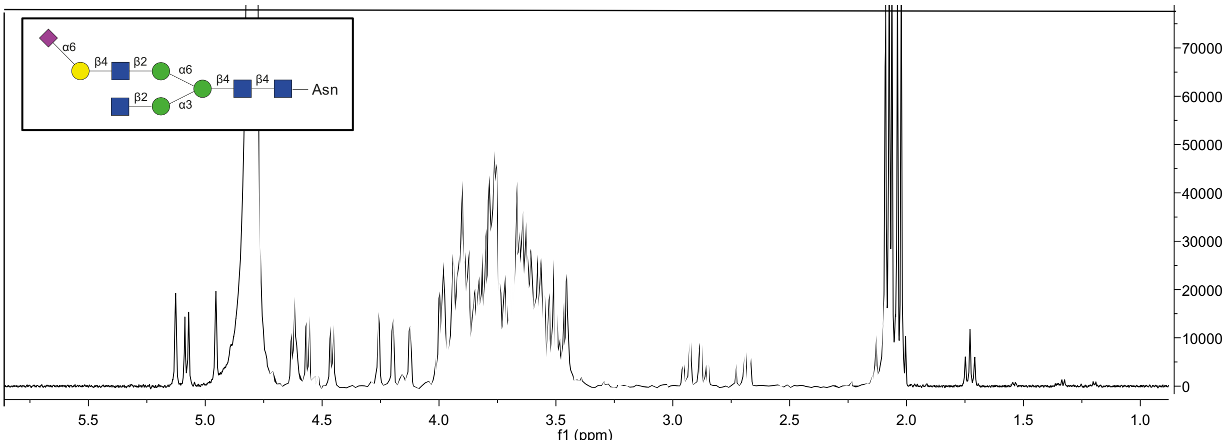

^{1}H 2; 600MHz; D_2O.

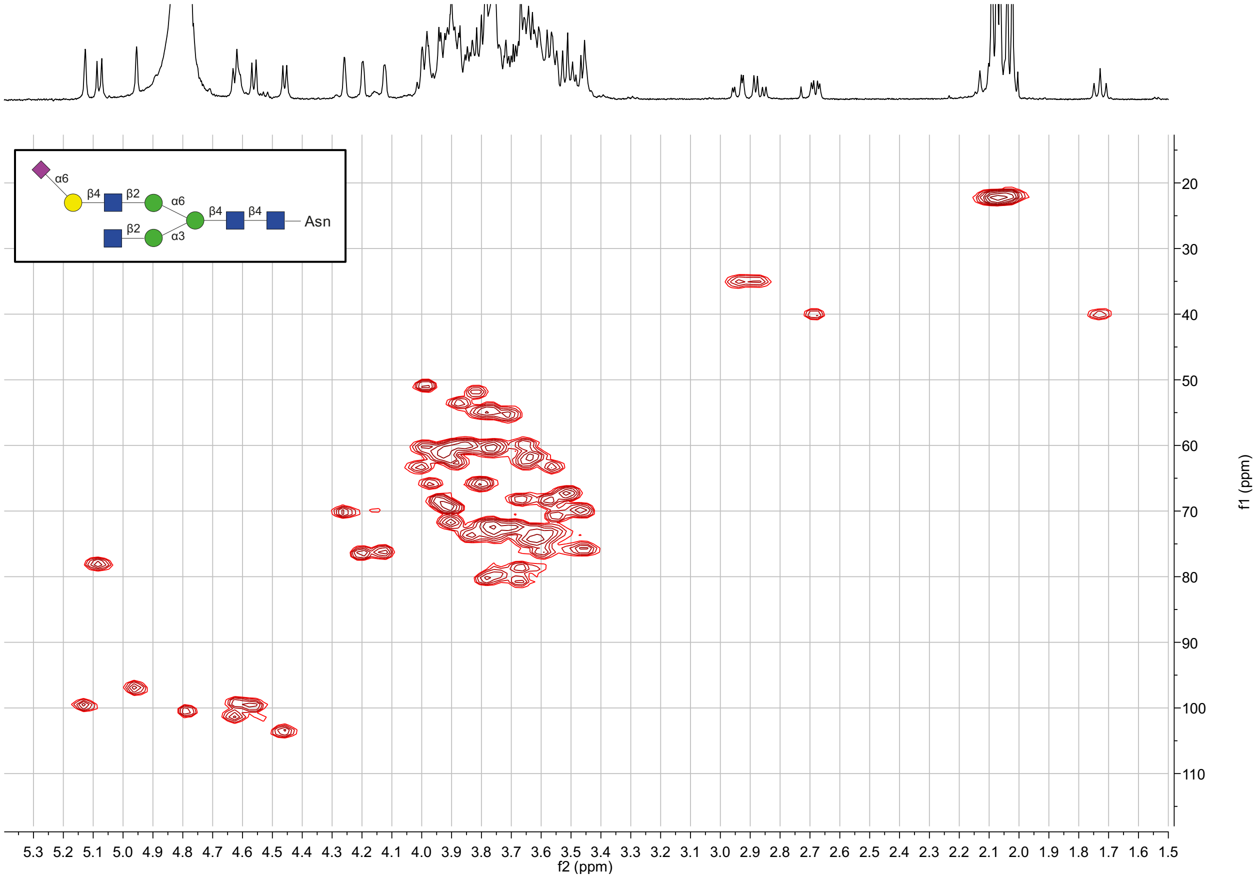

HSQC 2; 600 MHz/150 MHz; D_2O.

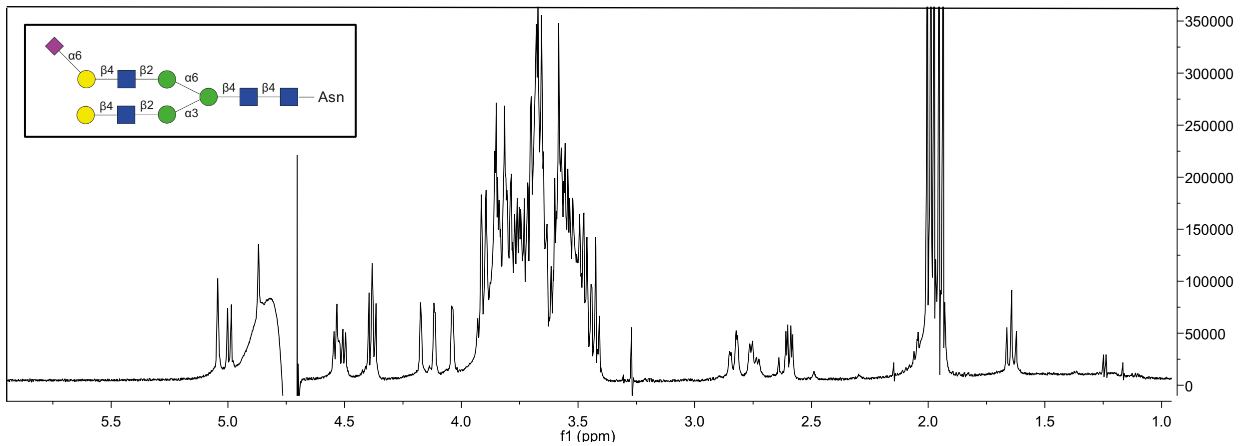

^{1}H 3; 600MHz; D_2O.

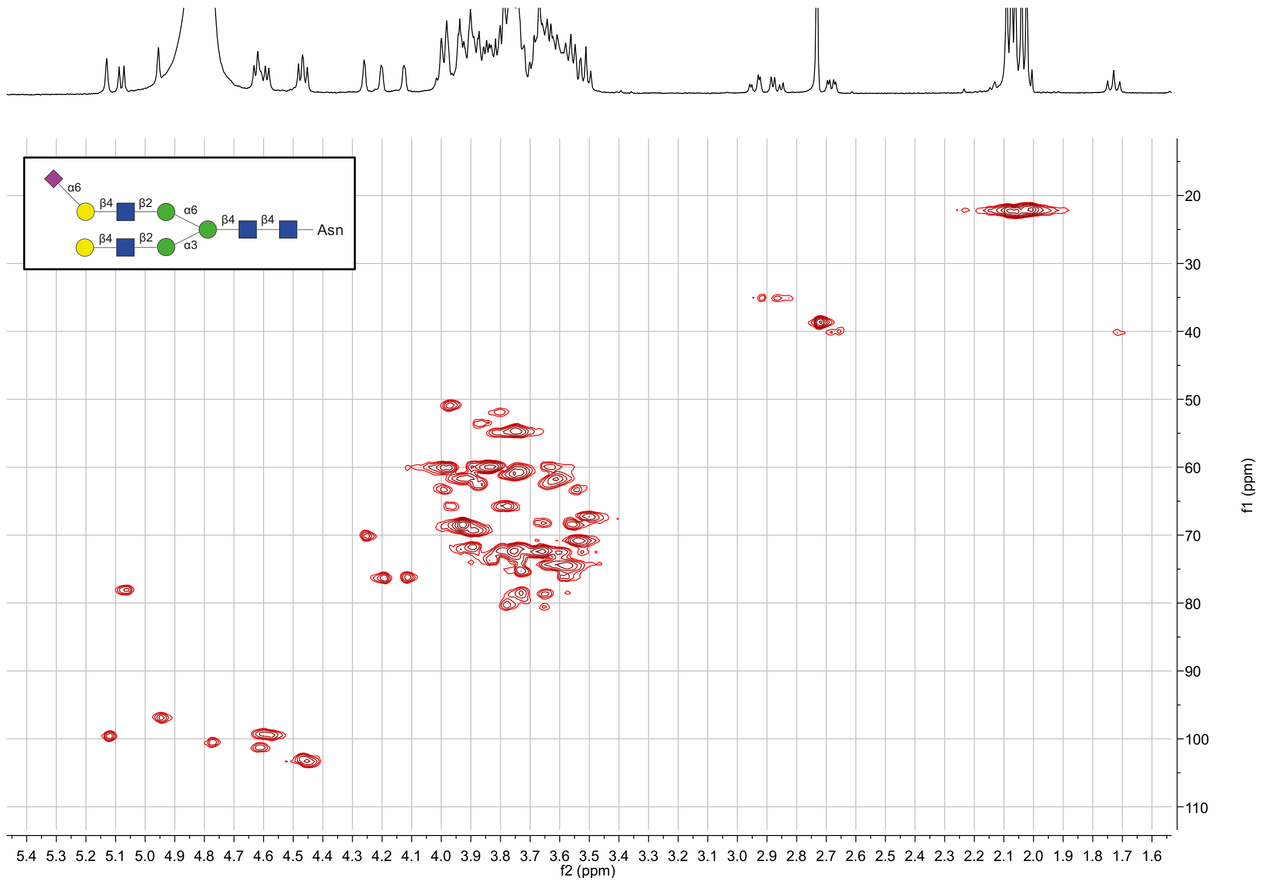

HSQC 3; 600 MHz/150 MHz; D_2O.

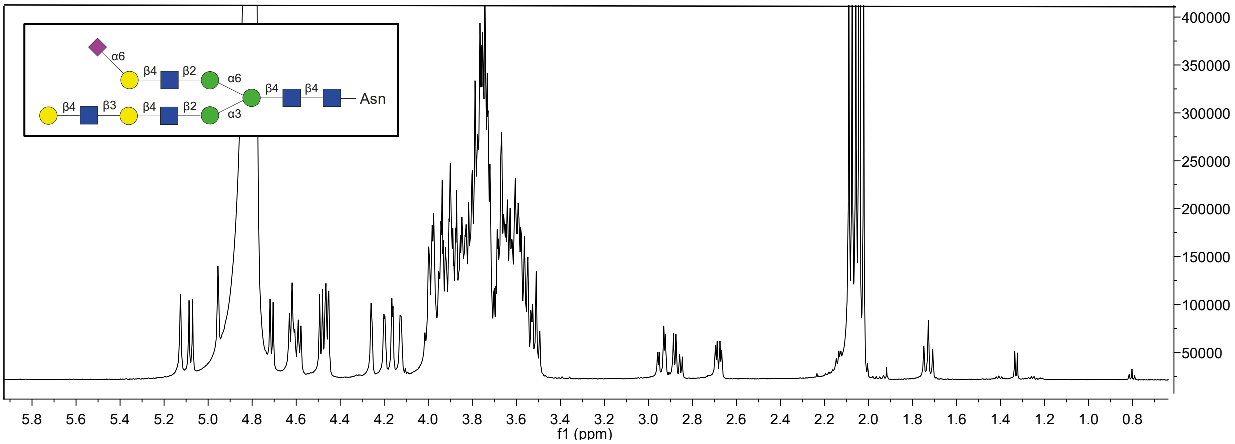

^{1}H 4; 600MHz; D_2O.

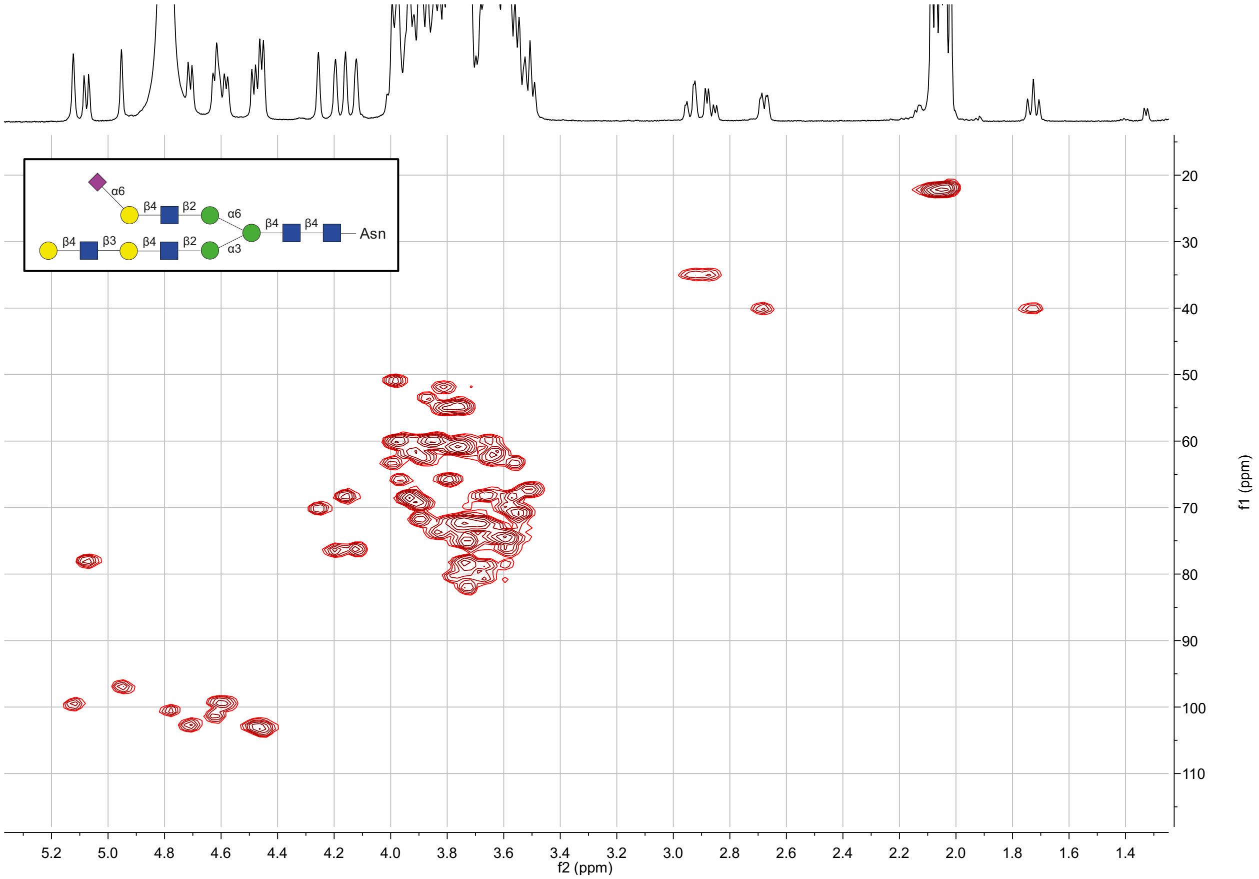

HSQC 4; 600 MHz/150 MHz; D_2O.

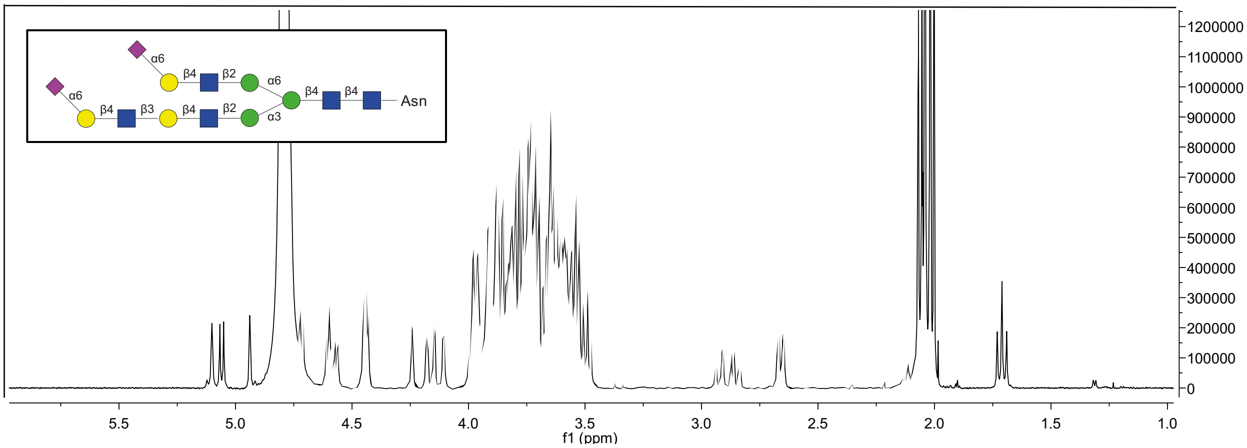

^{1}H **5; 600MHz; D_2O.**

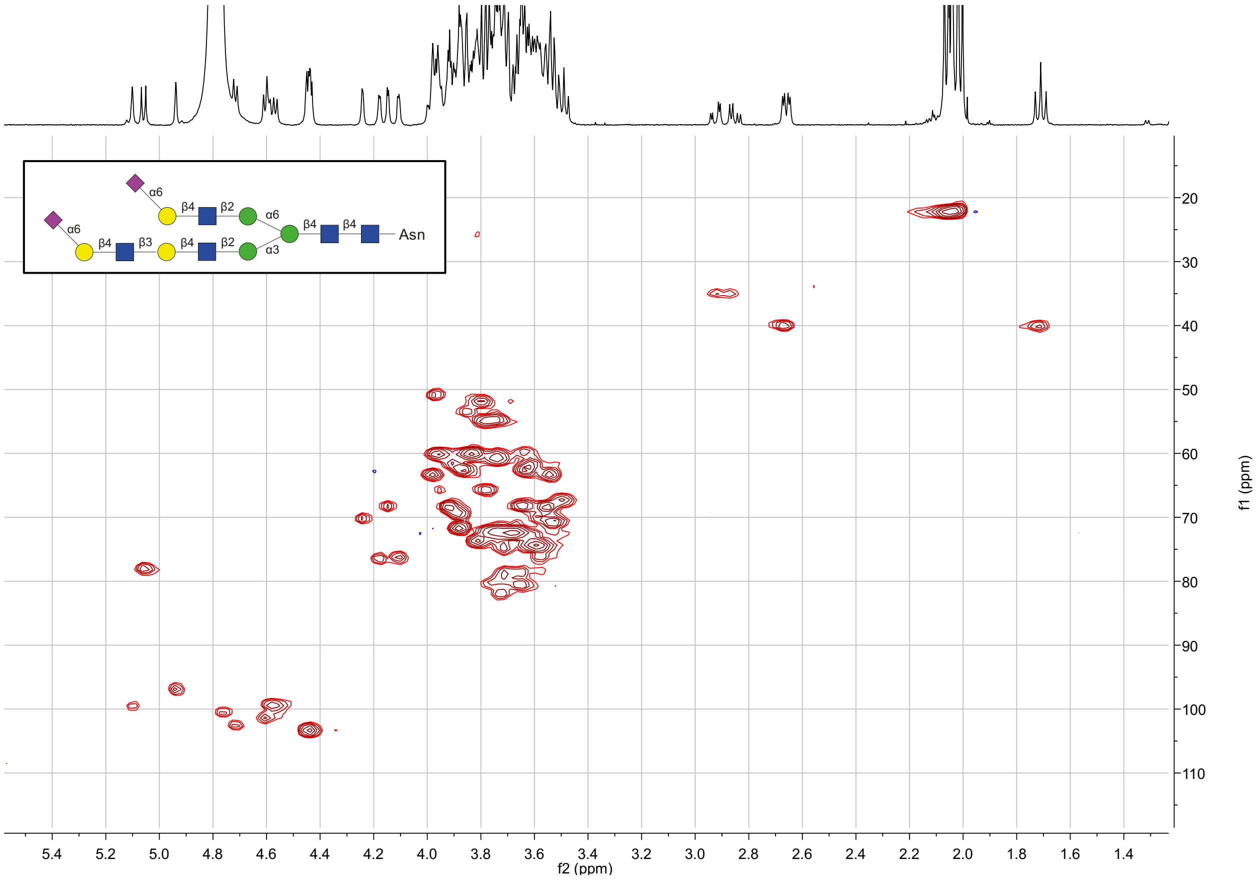

HSQC 5; 600 MHz/150 MHz; D_2O.

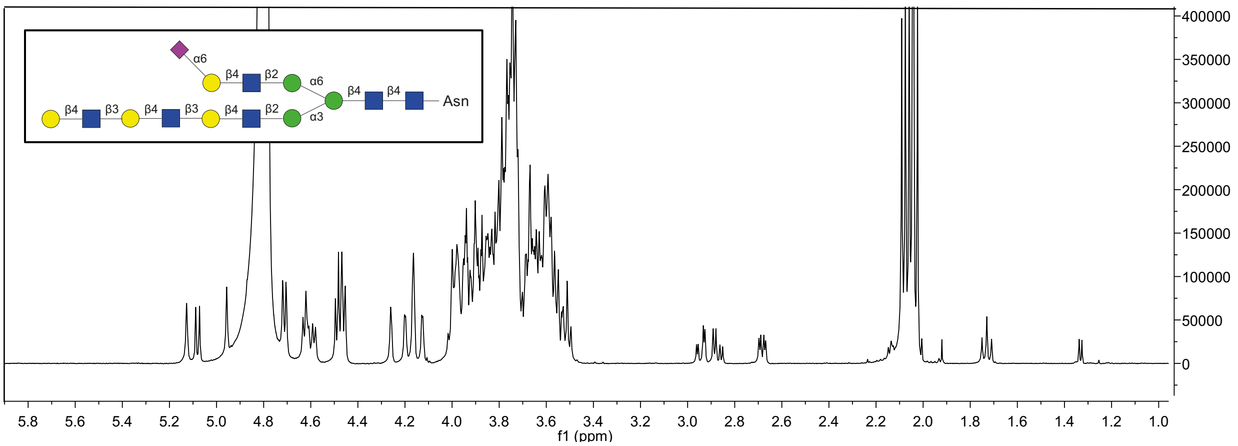

^{1}H 6; 600MHz; D_2O.

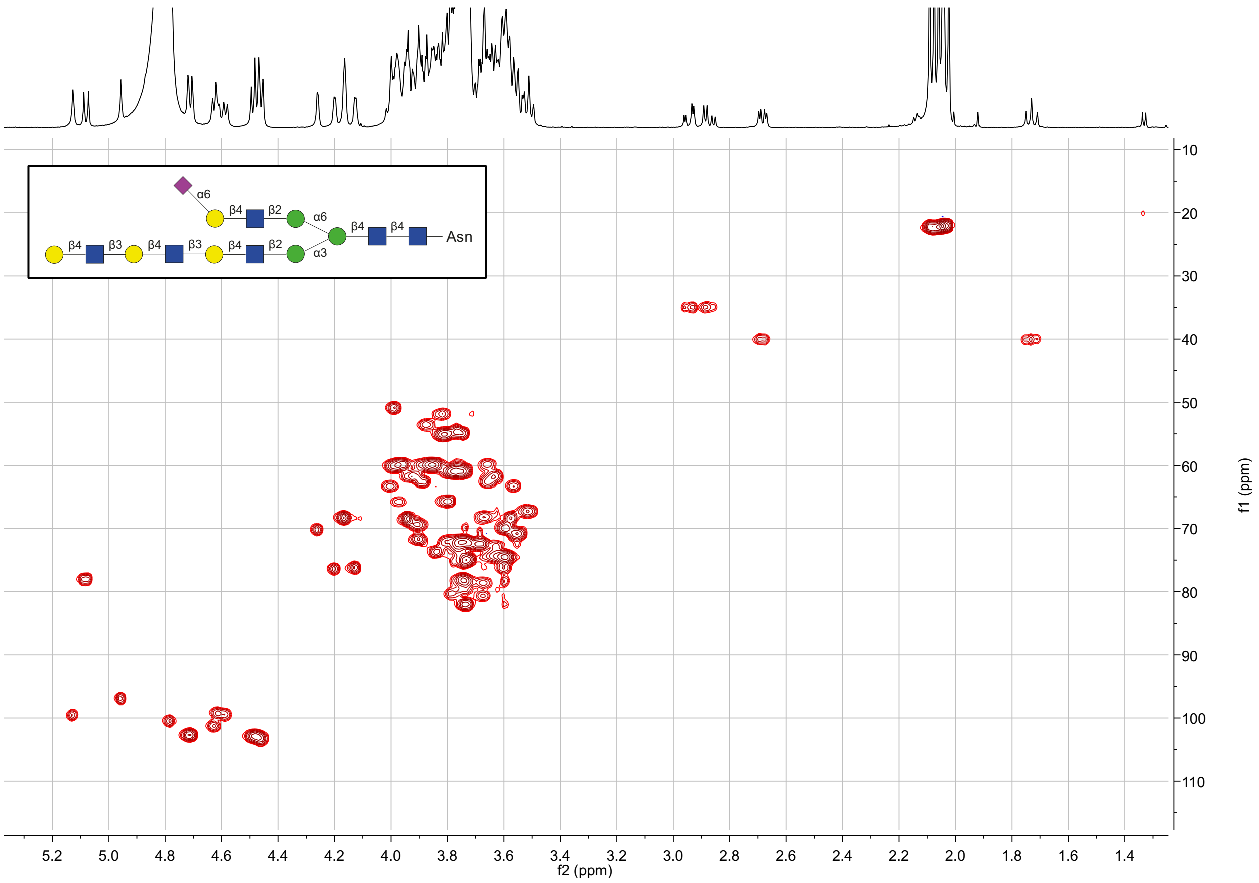

HSQC 6; 600 MHz/150 MHz; D_2O.

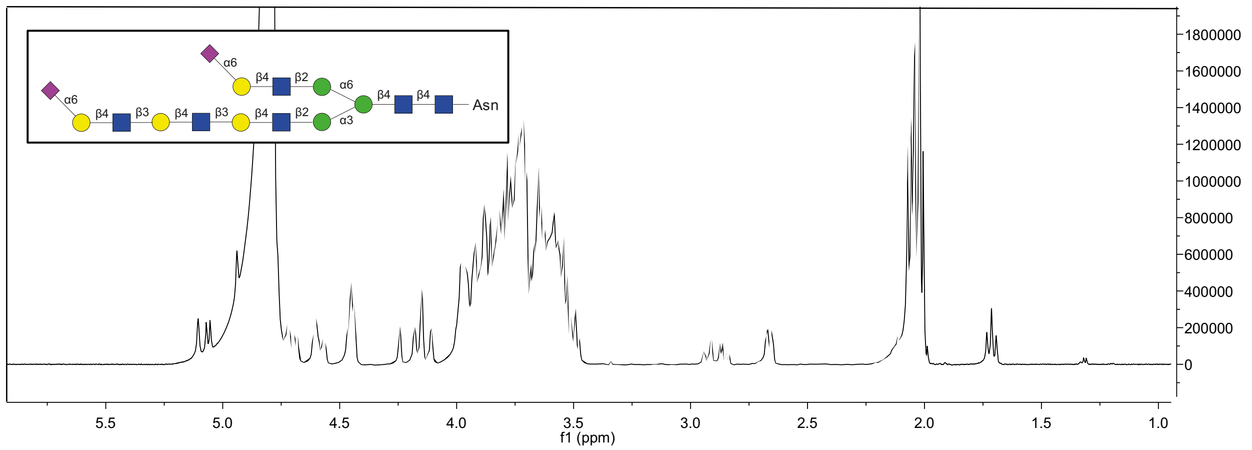

^{1}H **7; 600MHz; D_2O.**

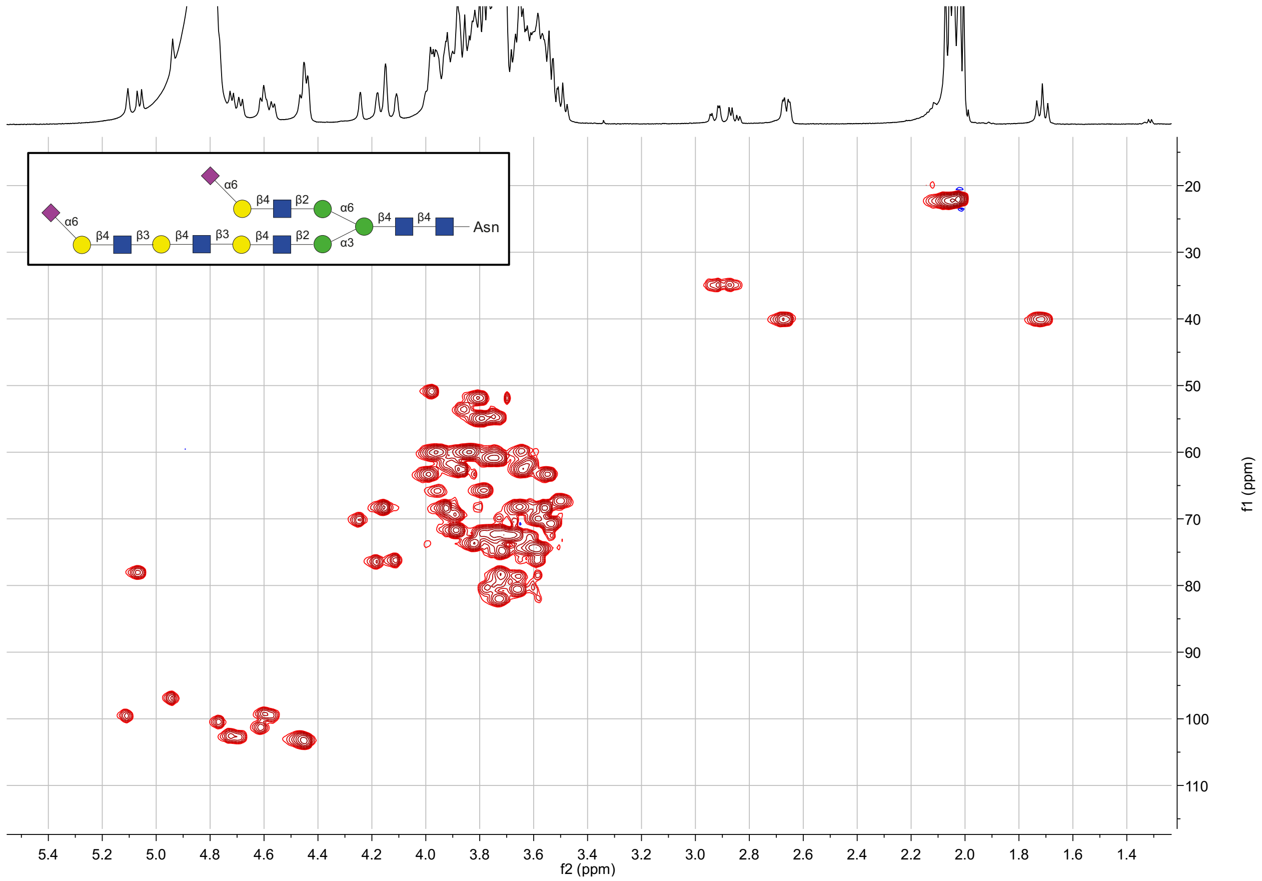

HSQC 7; 600 MHz/150 MHz; D_2O.

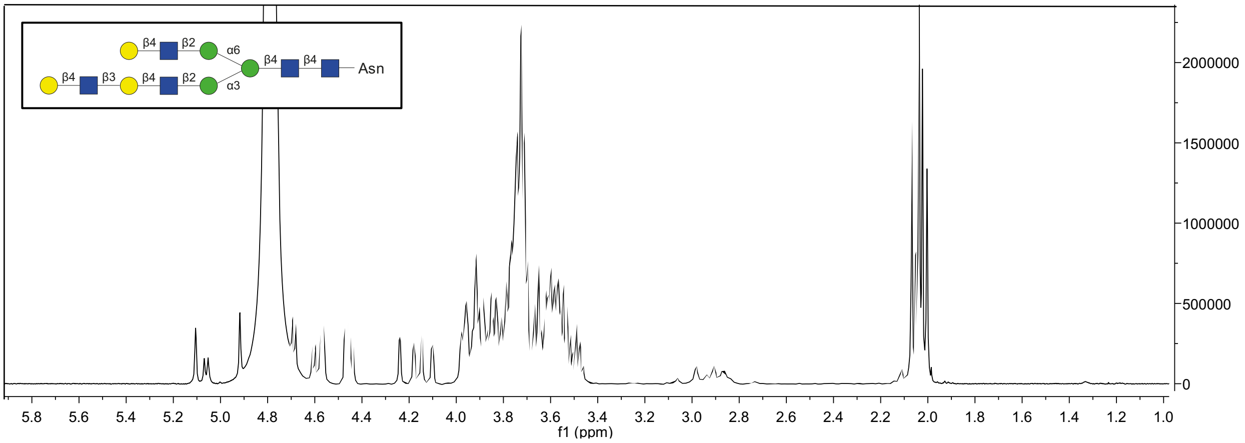

^{1}H 8; 600MHz; D_2O.

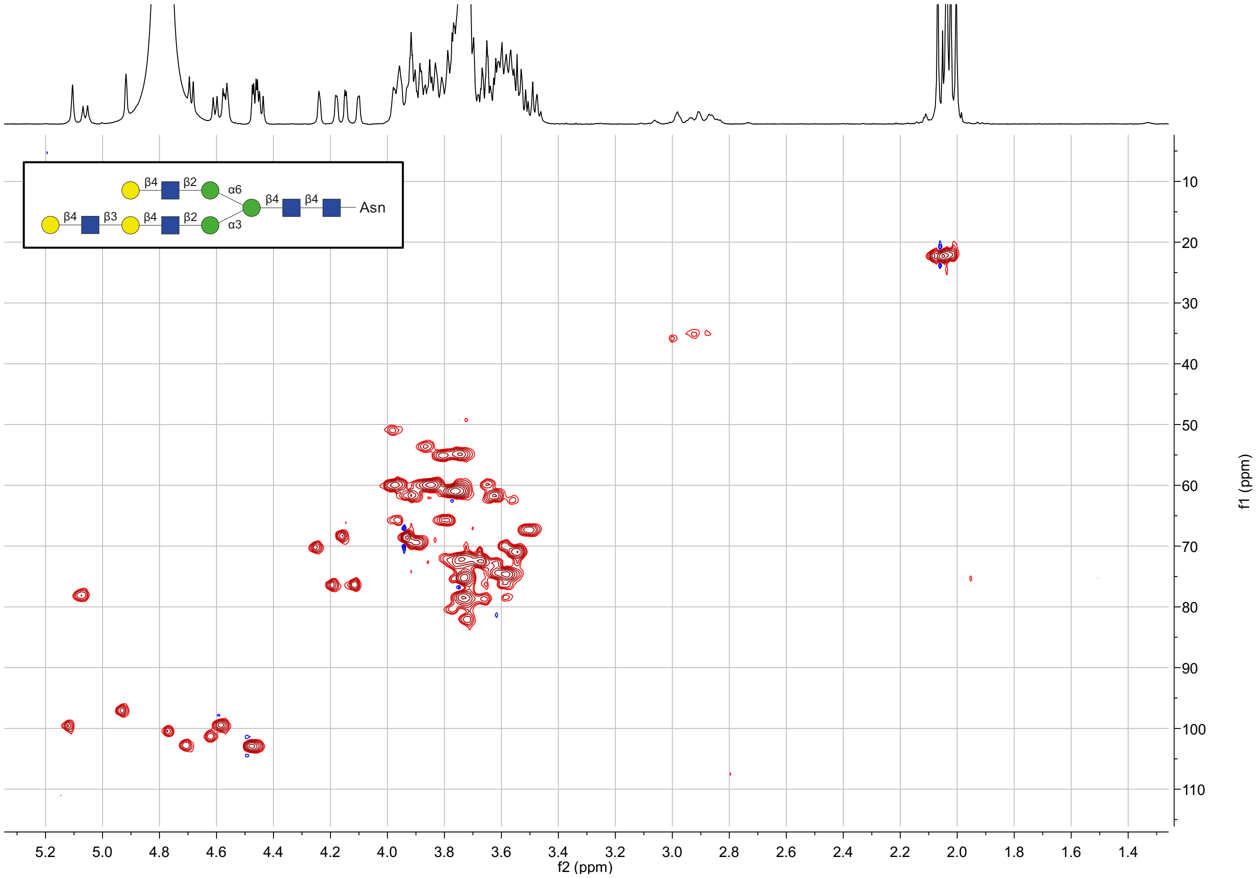

HSQC 8; 600 MHz/150 MHz; D_2O.

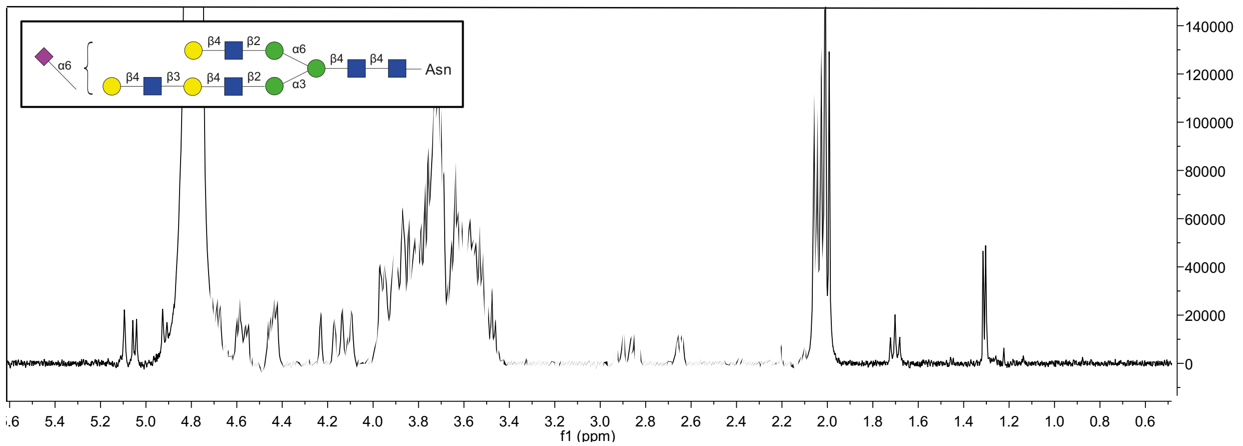

^{1}H **9; 600MHz; D_2O.**

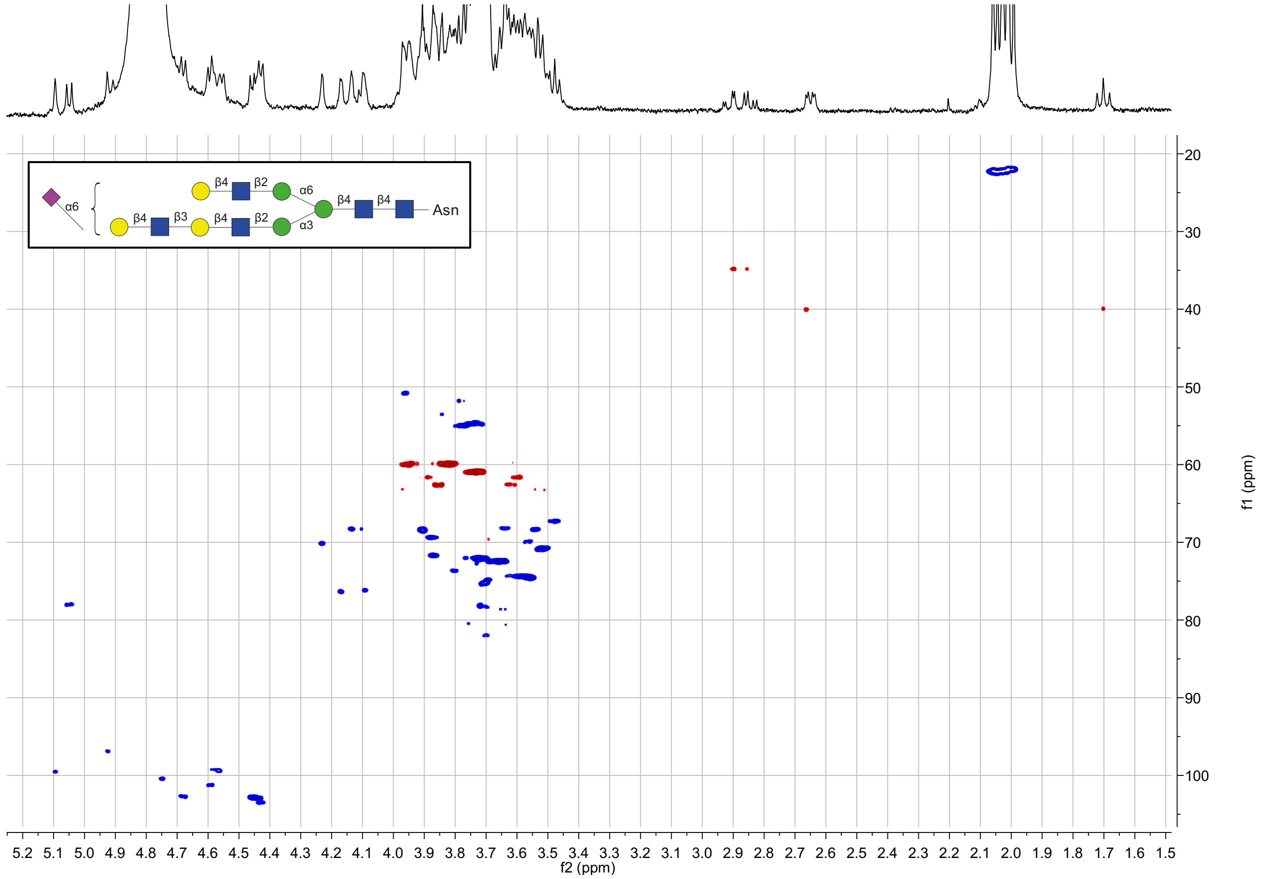

HSQC 9; 600 MHz/150 MHz; D_2O.

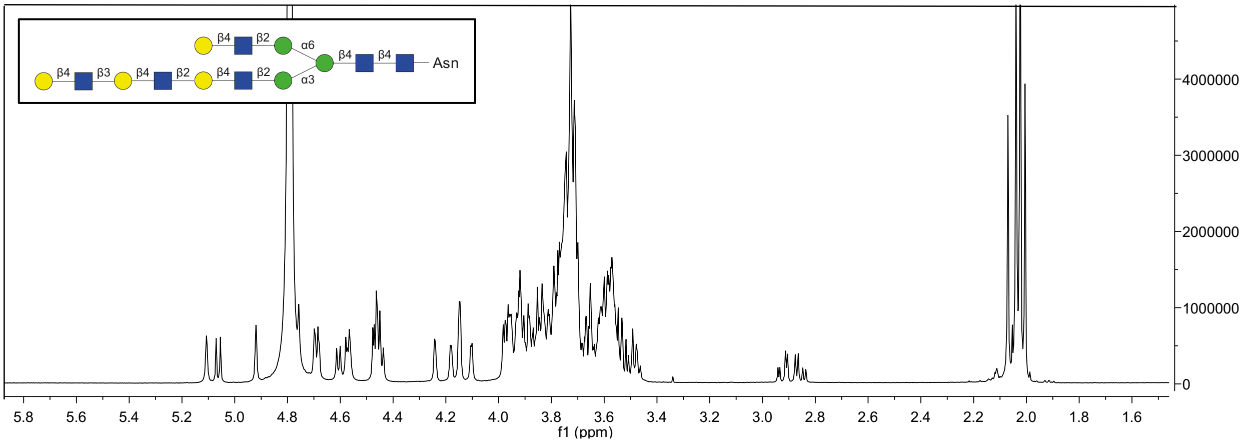

^{1}H 10; 600MHz; D_2O.

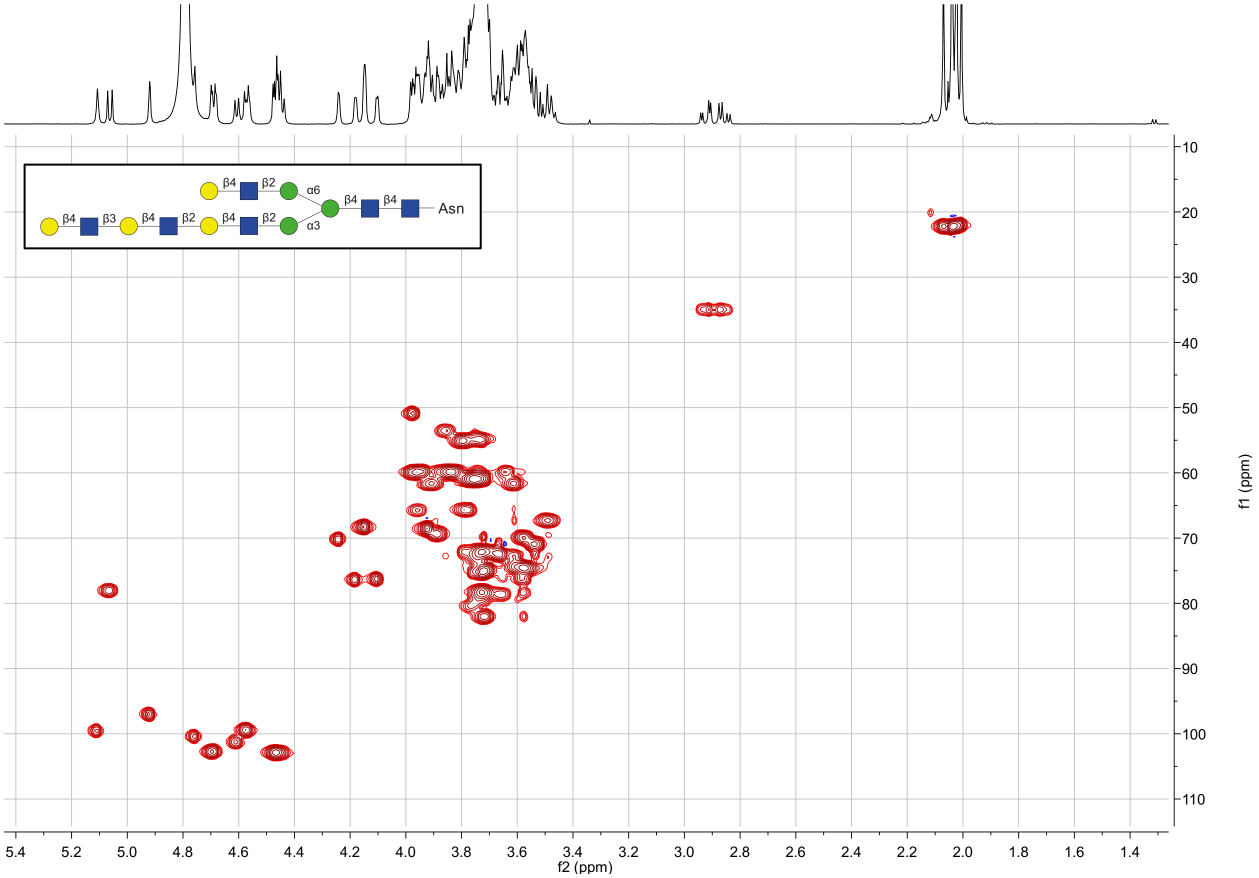

HSQC 10; 600 MHz/150 MHz; D_2O.

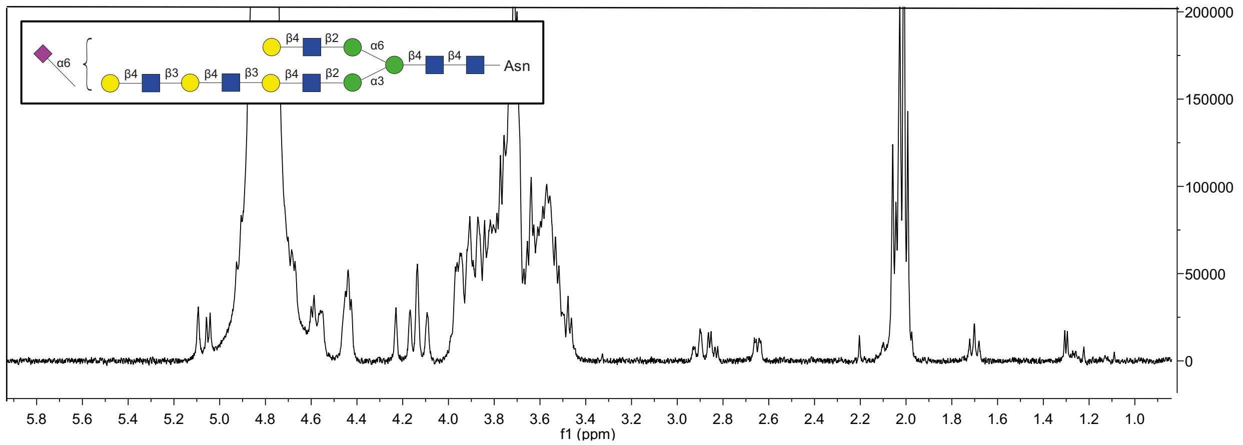

^{1}H 11; 600MHz; D_2O.

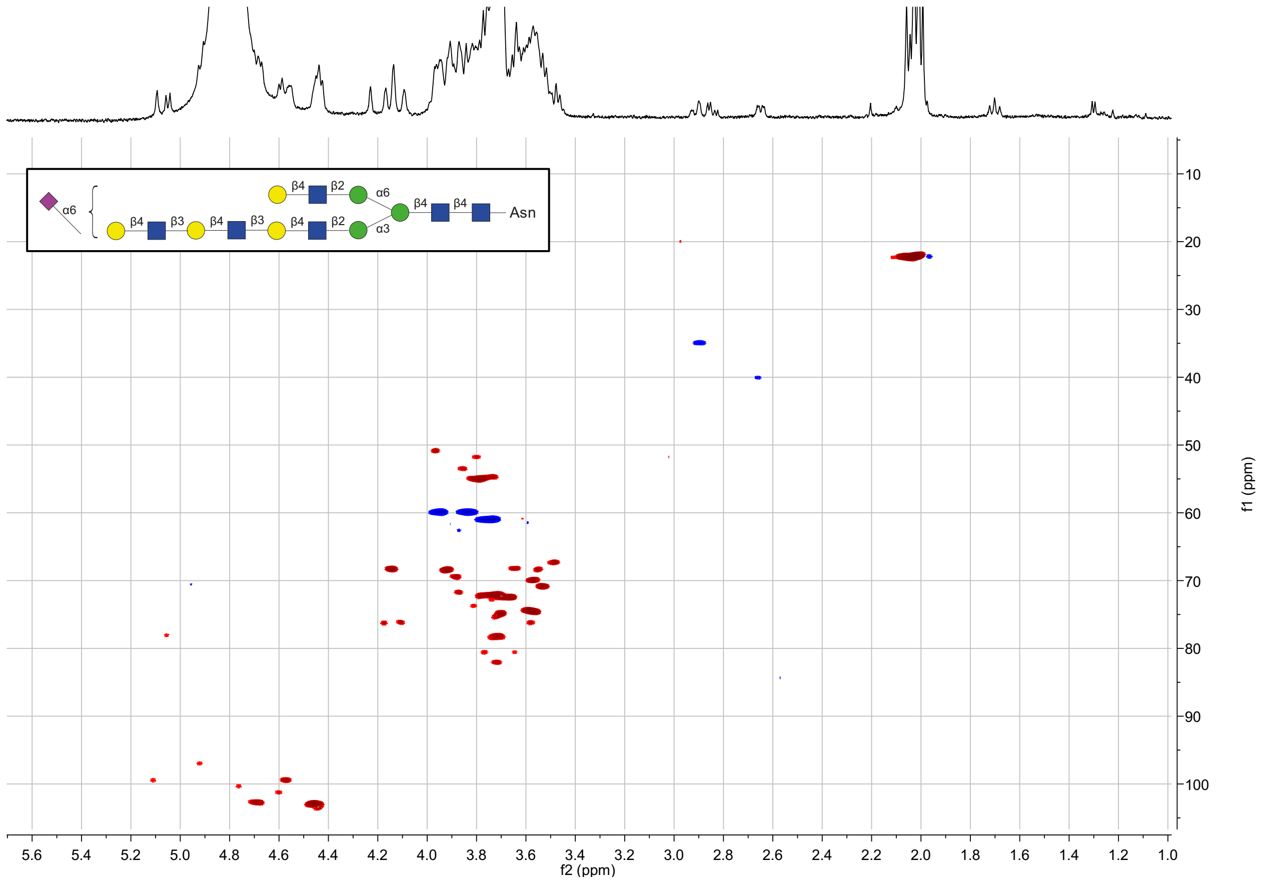

HSQC 11; 600 MHz/150 MHz; D_2O.

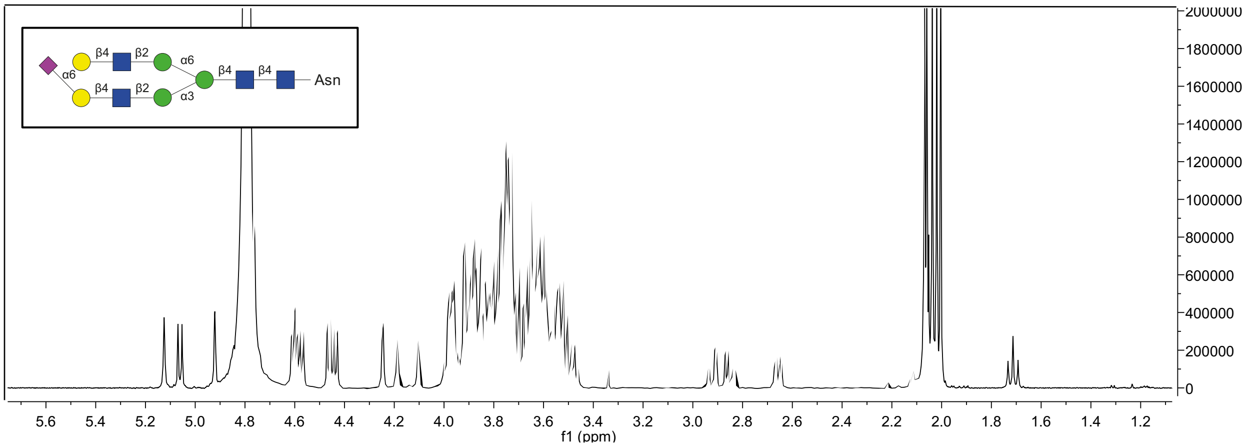

^{1}H 12; 600MHz; D_2O.

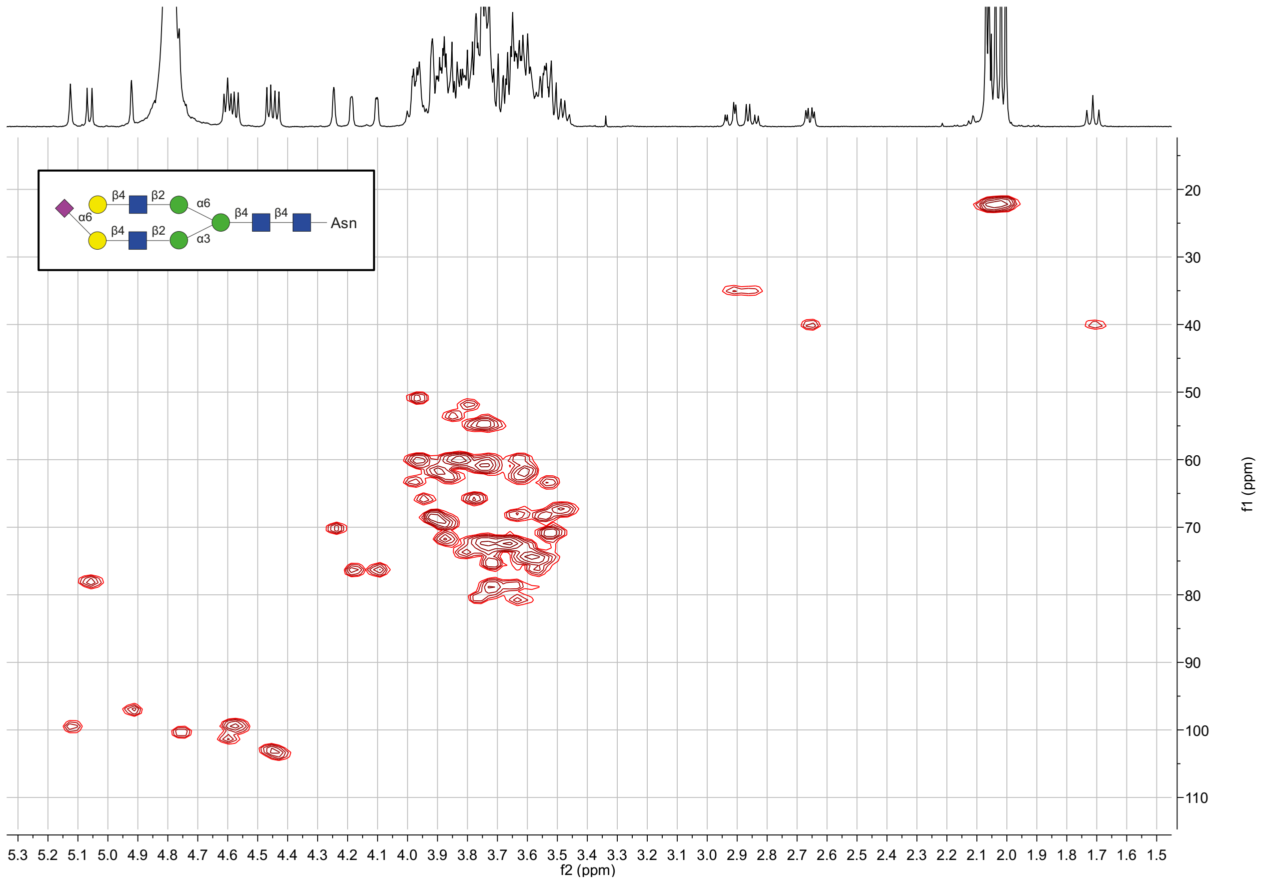

HSQC 12; 600 MHz/150 MHz; D_2O.

Extended data

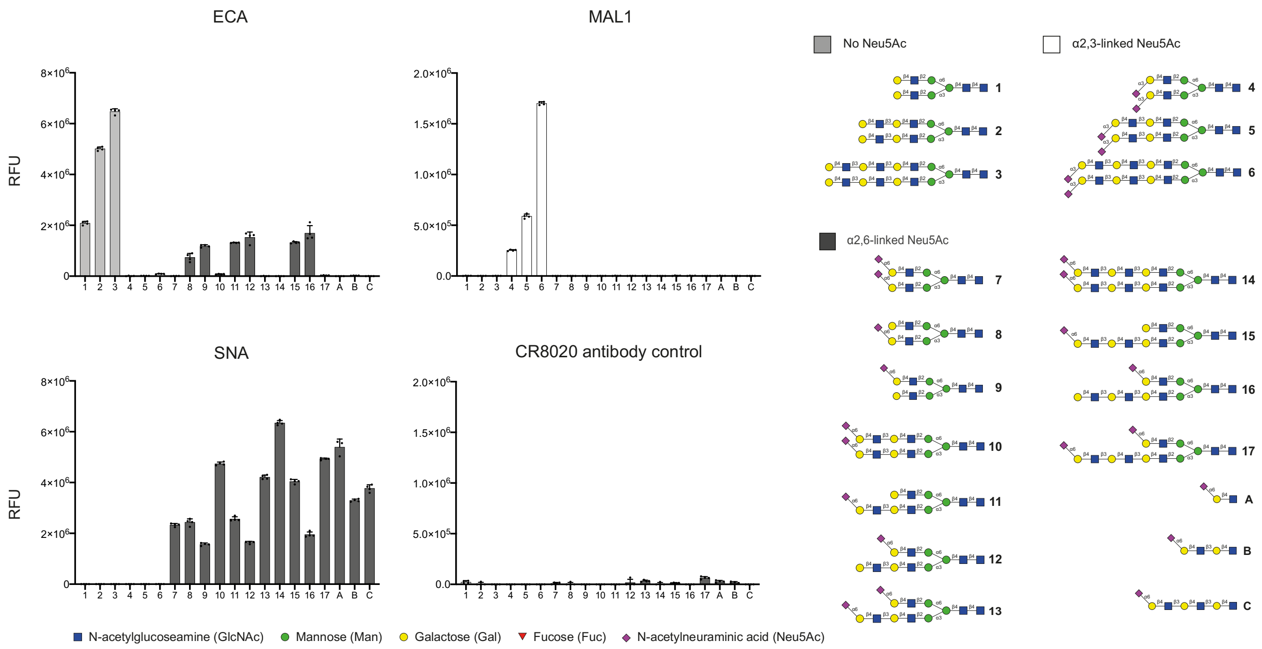

Supplementary Figure 1. Binding specificity of control lectins ECA, MAL1 and SNA and CR8020 A/H3N2 stem antibody. Glycans were printed on NHS glass slides and receptor specificity of the biotinylated lectins and the CR8020 antibody was visualized using Streptavidin-AlexaFluor-635 and a goat anti-human AlexaFluor-647 antibody. Bars represent the background-subtracted average relative fluorescence units (RFU) of four replicates ± SD. Values for all individual datapoints are represented in the Supplementary Source Data file. Glycans #1-#3: non-sialylated; #4-#6: α2,3-linked Neu5Ac; #7-#17: α2,6-linked Neu5Ac; A-C: linear α2,6-linked Neu5Ac.

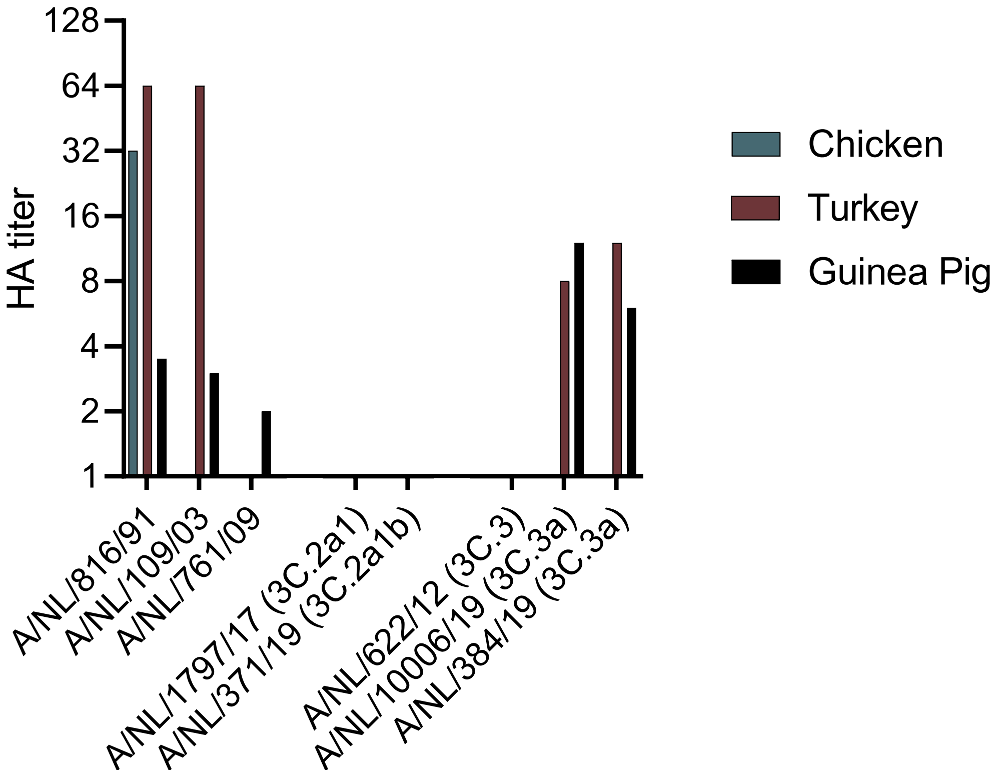

Supplementary Figure 2. Evolution of A/H3N2 hemagglutination ability of fowl and guinea pig erythrocytes. Hemagglutination (HA) titers are shown for a representative set of A/H3N2 viruses over the course of evolution in the last two decades. All HA assays were performed in the presence of 20 nM oseltamivir.

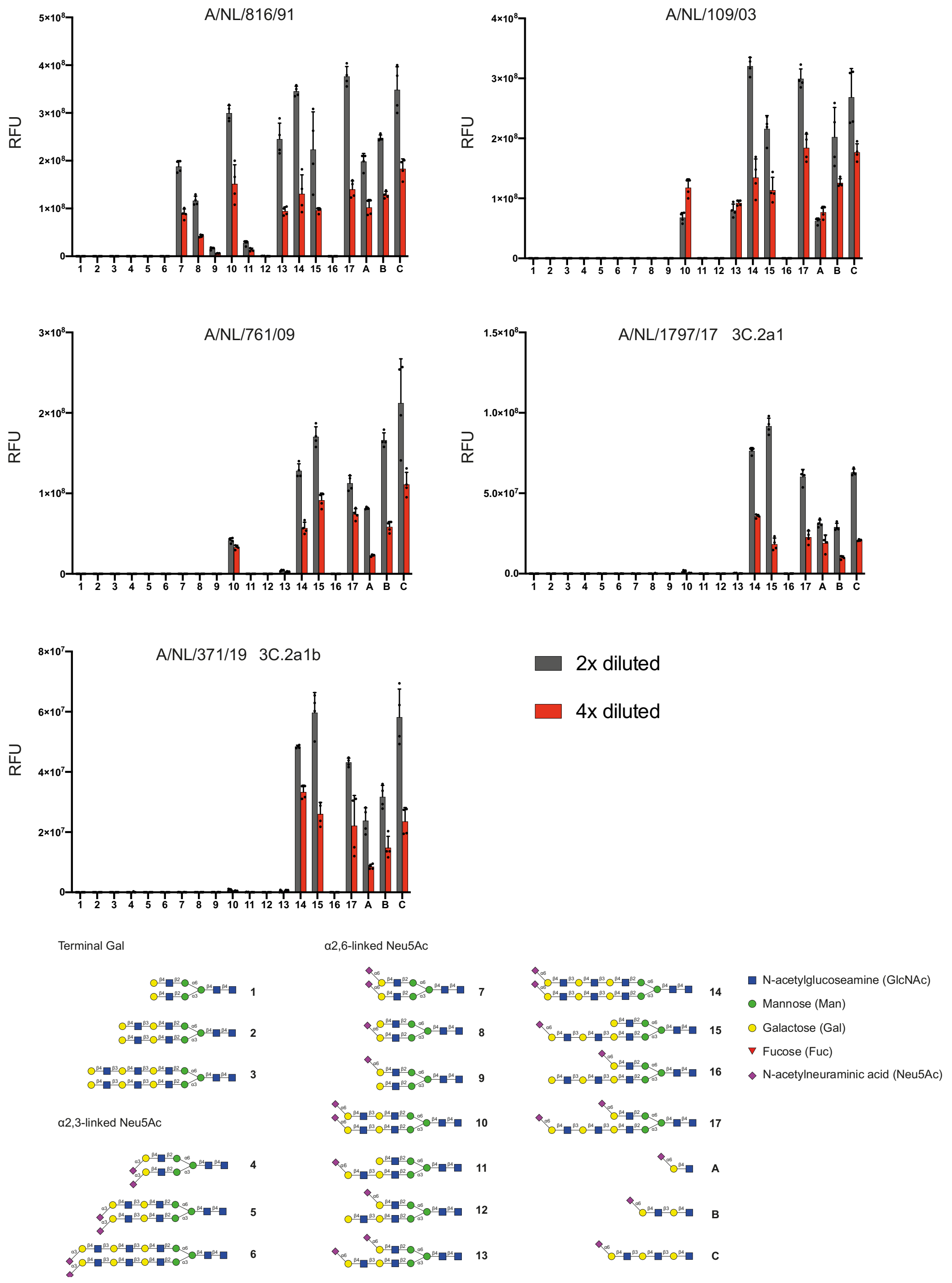

Supplementary Figure 3. Concentration-dependent response of representative A/H3N2 viruses in glycan microarray analysis. Glycans were printed on NHS glass slides and receptor specificity was visualized using the human CR8020 antibody followed with a goat anti-human AlexaFluor-555 antibody. Viruses were applied to the assay 2x and 4x diluted from the original isolate. Bars represent the background-subtracted average relative fluorescence units (RFU) of four replicates ± SD. Values for all individual datapoints are represented in the Supplementary Source Data file. Glycans #1-#3: non-sialylated; #4-#6: α2,3-linked Neu5Ac; #7-#17 and A-C: α2,6-linked Neu5Ac.

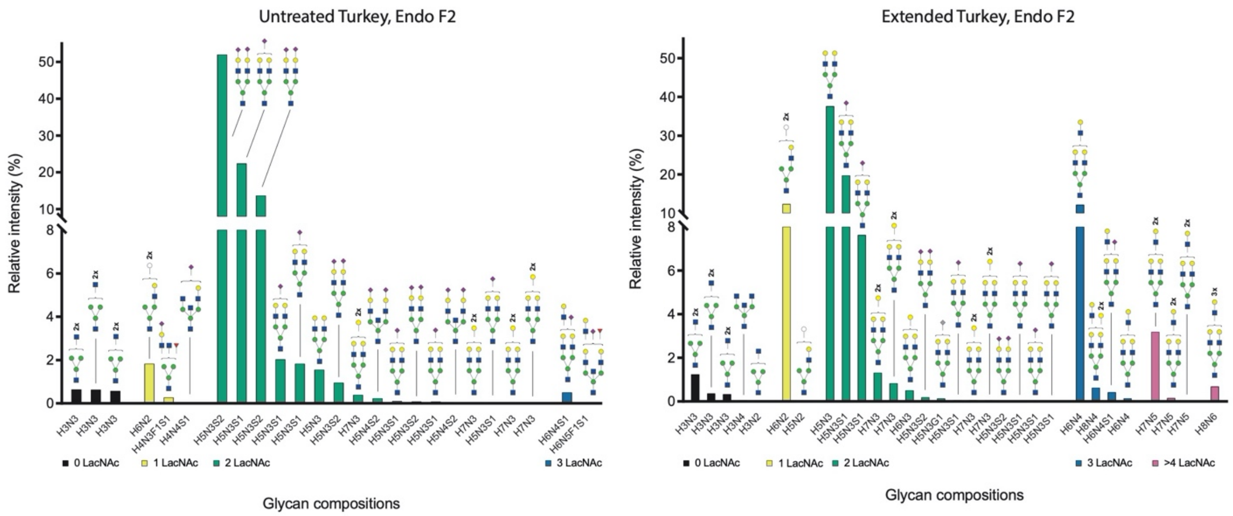

Supplementary Figure 4. Glycomic analysis of N-glycosylation of turkey erythrocytes released by Endo F2. The data is sorted by abundance and number of LacNAc units. Proposed structures are assigned to detected glycan compositions.

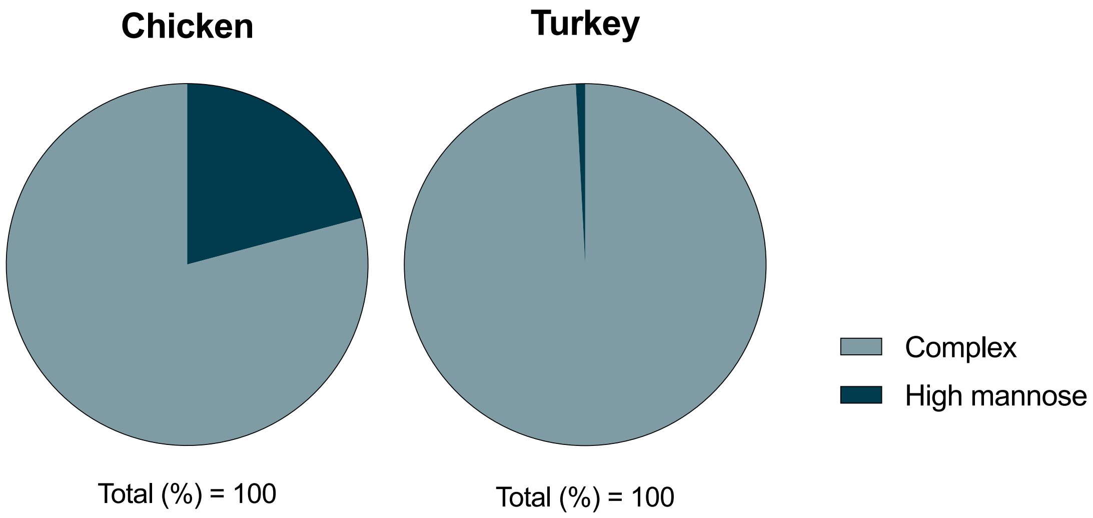

Supplementary Figure 5. Relative cell surface occupation of complex and high mannose glycans on chicken and turkey erythrocytes. The percentual distribution of complex and high mannose glycans was calculated based on the data from the glycomic analysis of untreated erythrocytes. The full overview of the glycomic data can be found in Data S1-S2.

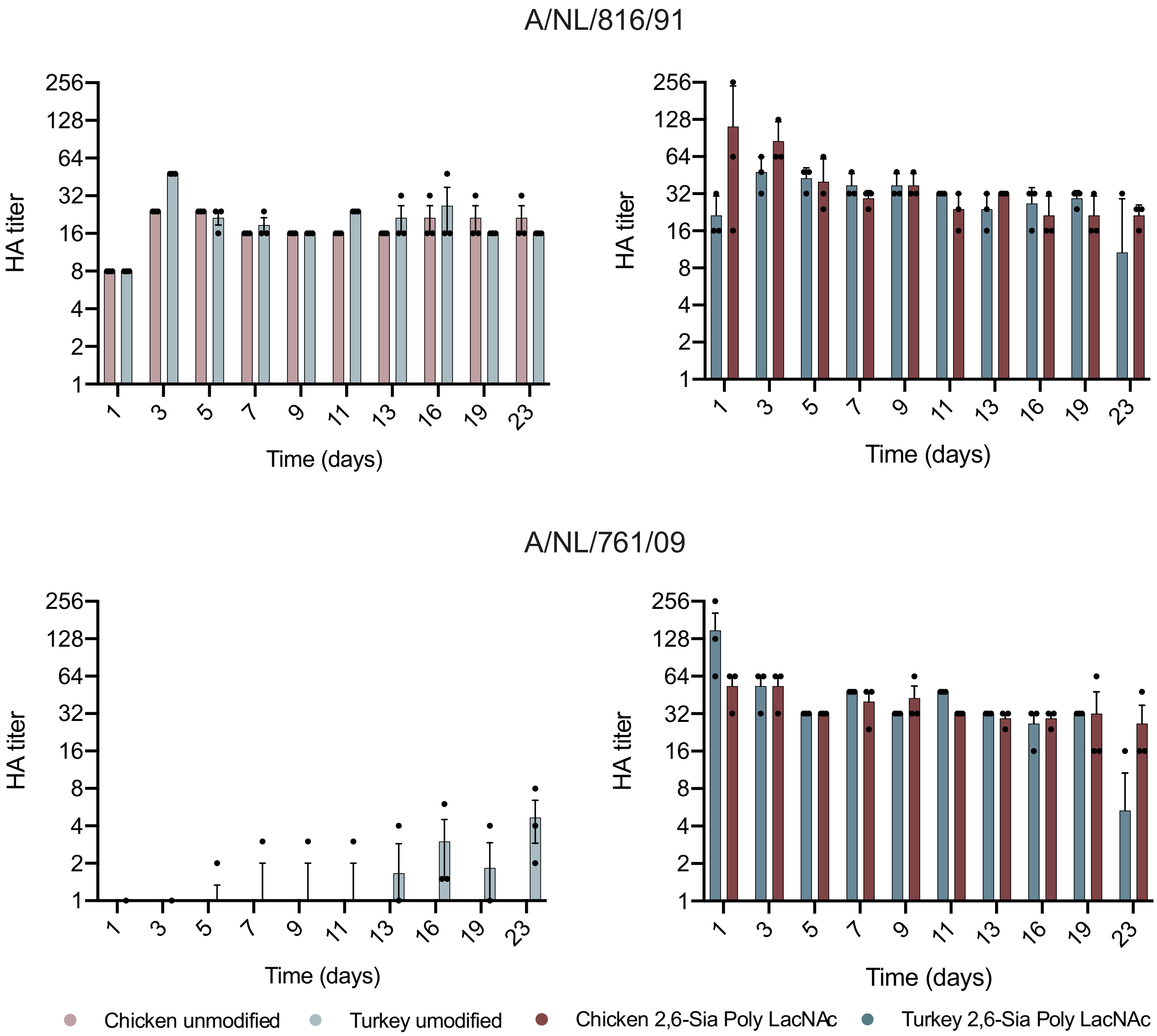

Supplementary **Figure 6. Stability assay of glyco-engineered erythrocytes**. Shown are the HA titers of A/H3N2 A/NL/761/09 and A/H3N2 A/NL/816/91 using unmodified (left) or glyco-engineered (right) erythrocytes from chicken (red) and turkey (blue). Glyco-engineering and HA assays were performed in full biological triplicates and means are plotted ± SEM.

Supplementary Table 1. Focus reduction assay of recent A/H3N2 virus isolates.

			Post-infection ferret sera raised against						
			NIB-104	A/NL/314/19	NIB-112	A/NL/3466/17	A/NL/1802/18	X-327	A/NL/384/19
**Virus**	**Passage**	**Clade**	3C.2a1a	3C.2a1b	3C.2a2	3C.2a2	3C.2a2	3C.3a	3C.3a
NIB-104 (A/Singapore/INFH-16-0019/16)	E7Mdck_Siat2hCK	3C.2a1a	240	80	240	*n.t.*	160	30	<20
A/Netherlands/1797/18	Mdck1Siat2hCK	3C.2a1	<20	160	<20	20	40	<20	<20
A/Netherlands/314/19	MdckSiat2hCK	3C.2a1b	<20	160	<20	30	60	<20	<20
A/Netherlands/10009/19	mixhCK2	3C.2a1b	<20	60	<20	*n.t.*	80	<20	<20
A/Netherlands/371/19	MdckSiatmixhCK	3C.2a1b	<20	240	<20	30	60	<20	<20
NIB-112 (A/Switzerland/8060/17)	E7E1hCK	3C.2a2	480	5120	5120	3840	240	<20	<20
A/Netherlands/3466/17	Siat1hCK1	3C.2a2	30	80	480	960	80	<20	<20
A/Netherlands/1802/18	Siat1hCK1	3C.2a2	<20	80	<20	30	640	<20	<20
A/Netherlands/10616/19	MdckSiatmixhCK	3C.2a2	<20	120	120	640	80	<20	<20
X327 (A/Kansas/014/17)	E17E1Siat1hCK	3C.3a	<20	<20	<20	<20	<20	960	30
A/Netherlands/384/19	Siat2hCK	3C.3a	<20	<20	30	<20	30	40	80
A/Netherlands/10006/19	MdckSiatmixhCK2	3C.3a	<20	<20	<20	<20	<20	20	60

n.t. = not tested

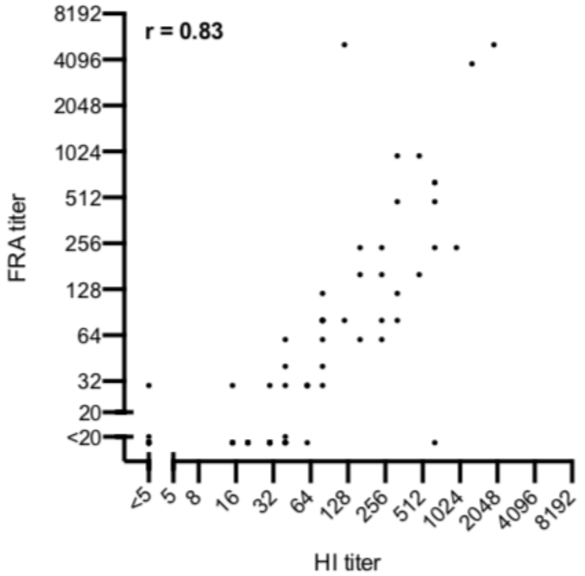

Supplementary Figure 7. Correlation of hemagglutination inhibition and focus reduction titers by recent A/H3N2 viruses. Shown is the correlation between every HI titer and FRA titer for all viruses and sera as depicted in Table 1 and Supplementary Table **1**. The titers of the focus reduction assay duplicates were averaged for the graph. Spearman's rank correlation coefficient was measured using Prism 8.3.1 (Graphpad).

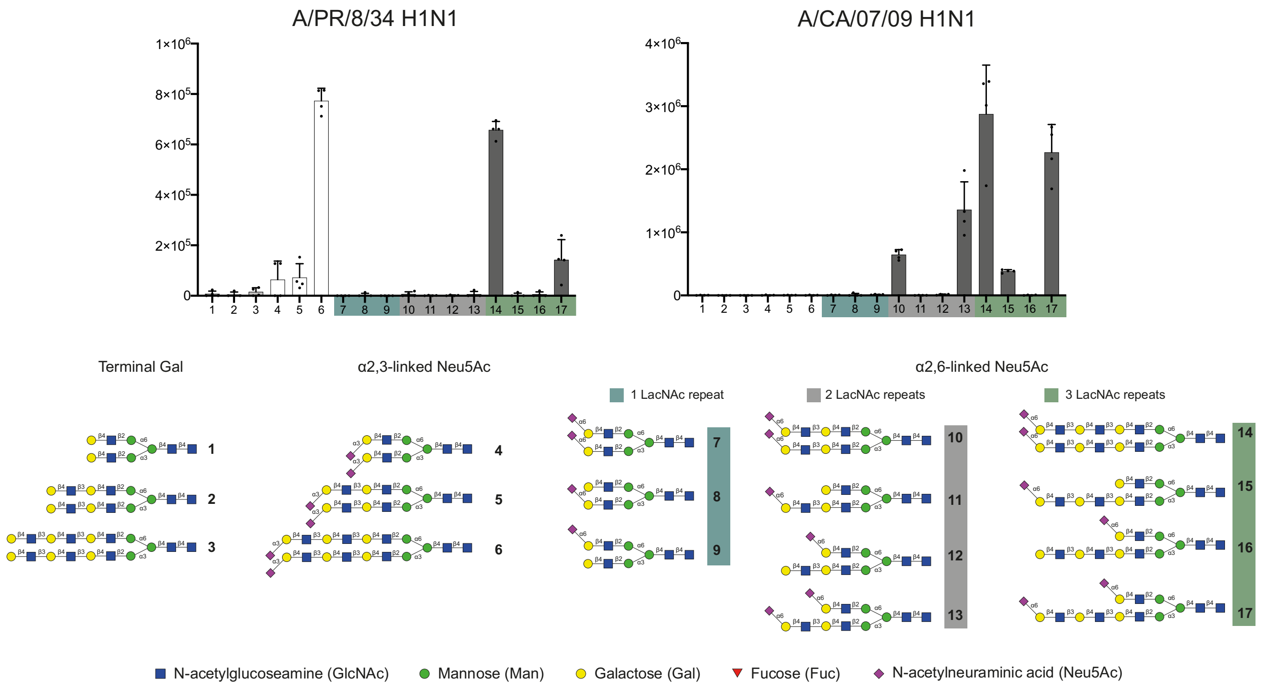

Supplementary Figure 8. **Receptor binding specificities of representative H1N1 viruses using glycan microarray analysis.** Binding was visualized using a human anti-H1 stalk antibody (CR6261). Bars represent the background-subtracted average relative fluorescence units (RFU) of four replicates ± SD. Values for all individual datapoints are represented in the Supplementary Source Data file.

Supplementary Table 2. Sequence analysis. Alignment of A/H3N2 HA sequence spanning from 1968 to 2019. Amino acid mutation refers to the A/HK/1/68 sequence. Amino acids substitutions are labelled in color code according to the changes in polarity: mutations that led to amino acids (aa) of similar polarity are labelled in gray, mutations that led to less polar aa are in pink, mutations to more polar uncharged residue are labeled in light blue, mutations to amino acids with negative charged side chain are labeled in red, mutations to amino acids with positive charged side chain are labeled in dark blue. New occurring glycosylation sites are labeled in green.

																aa sequence									aa seq										aa sequence			
Subtype	Year	Id	98	131	133	134	135	136	137	143	144	145	146	153	155	156	157	158	159	160	182	183	188	189	190	193	194	195	196	221	222	223	224	225	226	227	228
H3N2	1968	A/Hong Kong/1/68	Y	T	N	G	G	S	N	P	G	S	G	W	T	K	S	G	S	T	V	H	N	Q	E	S	L	Y	V	P	W	V	R	G	M	S	S
H3N2	1991	A/Netherlands/816/91	Y	A	S	G	E	S	Y	S	V	K	S	W	H	E	S	E	Y	T	V	H	D	R	E	S	L	Y	V	P	W	V	R	G	L	S	S
H3N2	1991	A/Siena/3/91	Y	A	S	G	E	S	Y	S	V	K	S	W	H	E	S	D	Y	K	V	H	D	R	E	S	L	Y	V	P	W	V	R	G	L	S	S
H3N2	2003	A/Netherlands/109/03	Y	T	N	G	T	S	S	S	N	K	S	W	T	H	L	K	Y	K	V	H	D	S	D	S	L	Y	A	P	R	V	R	D	I	S	S
H3N2	2003	A/New York/18/03	Y	T	N	G	T	S	S	S	N	K	S	W	T	H	L	K	Y	R	V	H	D	S	D	N	L	Y	A	P	R	V	R	D	V	S	S
H3N2	2005	A/Wisconsin/67/05	Y	T	N	G	T	S	S	S	N	N	S	W	T	H	L	K	F	K	V	H	D	N	D	F	L	Y	A	P	R	I	R	N	I	P	S
H3N2	2007	A/Brisbane/10/07	Y	T	N	G	T	S	S	S	N	N	S	W	T	H	L	K	F	K	V	H	D	N	D	F	L	Y	A	P	R	V	R	N	I	P	S
H3N2	2009	A/Perth/16/09	Y	T	N	G	T	S	S	S	K	N	S	W	T	H	L	N	F	K	V	H	D	K	D	F	L	Y	A	P	R	V	R	N	I	P	S
H3N2	2009	A/Netherlands/761/09	Y	T	N	G	T	S	S	S	N	N	S	W	T	H	L	R	F	K	V	H	D	N	D	F	L	Y	A	P	R	V	R	N	I	P	S
H3N2	2011	A/Victoria/361/11	Y	T	N	G	T	S	S	S	N	N	S	W	T	Q	L	N	F	K	V	H	D	K	D	F	L	Y	A	P	R	I	R	N	I	S	S
H3N2	2012	A/Texas/53/12	Y	T	N	G	T	S	S	S	N	N	S	W	T	H	L	N	F	K	V	H	D	K	D	F	L	Y	A	P	R	I	R	N	I	P	S
H3N2	2014	A/Hong Kong/4801/14	Y	T	N	G	T	S	S	S	S	S	S	W	T	H	L	N	Y	T	V	H	D	K	D	F	L	Y	A	P	R	I	R	D	I	P	S
H3N2	2016	A/Singapore/INFH-16-0019/16	Y	T	N	G	T	S	S	S	S	S	S	W	T	H	L	N	Y	T	V	H	D	K	D	F	L	Y	A	P	R	I	R	D	I	P	S
H3N2	2017	A/Netherlands/1797/17	Y	T	N	G	K	S	S	S	S	S	S	W	T	H	L	N	Y	T	X	H	D	K	D	F	L	Y	A	P	R	I	R	D	I	P	S
H3N2	2019	A/Netherlands/371/19	Y	T	N	G	K	S	S	S	S	S	S	W	T	H	L	N	Y	T	X	H	D	K	D	F	L	Y	A	P	R	I	R	D	I	P	S
H3N2	2013	A/Swiss/9715293/13	Y	T	N	G	T	S	S	S	N	S	S	W	T	H	L	N	S	K	V	H	D	K	D	F	L	Y	A	P	R	I	R	D	I	P	S
H3N2	2012	A/Netherlands/622/12	Y	T	N	G	T	S	S	S	N	S	S	W	T	H	L	N	F	K	V	H	D	K	D	F	L	Y	A	P	R	I	R	N	I	P	S
H3N2	2016	A/Netherlands/153/16	Y	T	N	G	T	S	S	S	N	S	S	W	T	H	L	N	S	K	V	H	D	K	D	F	L	Y	A	P	R	I	R	D	I	P	S
H3N2	2017	A/Kansas/14/17	Y	T	N	G	T	S	S	S	K	S	S	W	T	H	L	N	S	K	V	H	D	K	D	S	L	Y	A	P	R	I	R	D	I	P	S
H3N2	2019	A/Netherlands/10002/19	Y	T	N	G	T	S	S	S	K	S	S	W	T	H	L	N	S	K	V	H	D	K	D	S	L	Y	A	P	R	I	R	D	I	P	S
Consensus Sequence			Y	T	N	G	T	S	S	S	.	S	S	W	T	H	L	N	.	K	V	H	D	K	D	F	L	Y	A	P	R	I	R	.	I	P	S

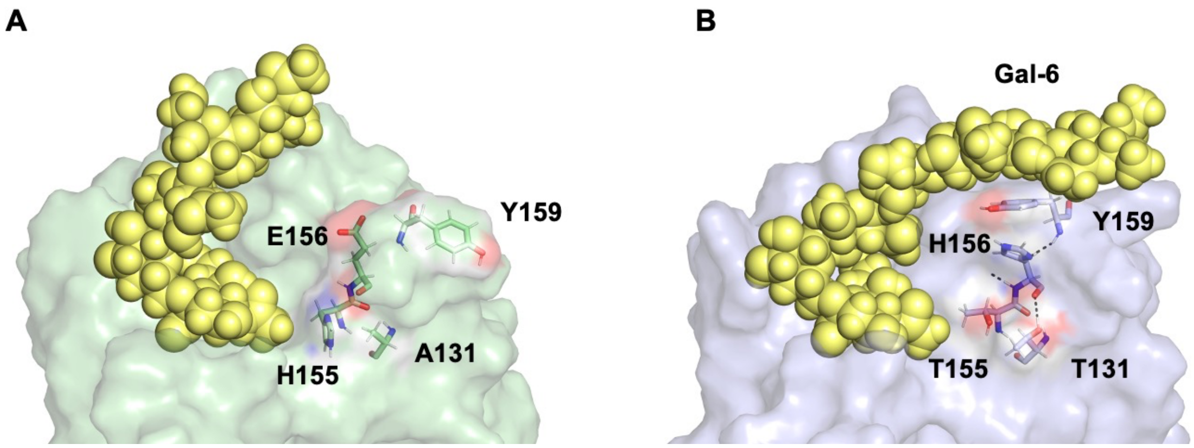

Supplementary Figure 9. Comparison of the last frames from MD simulation of (a) NL91 and (b) NL03 highlighting the amino acid substitutions which shaped the orientation of the side chain of Y159. The CH-π interaction with the proximal Gal-6 is possible only for NL/03 due to the favorable interaction between the H156 and Y159 rings. On the other hand, the unfavorable contacts between the negatively charged Glu156 and the aromatic ring displaces the Y159 side chain away from the Gal-6 residue, hampering the CH-π interaction. The H-bond network which shape the orientation of Y159 is defined by the dashed black line.

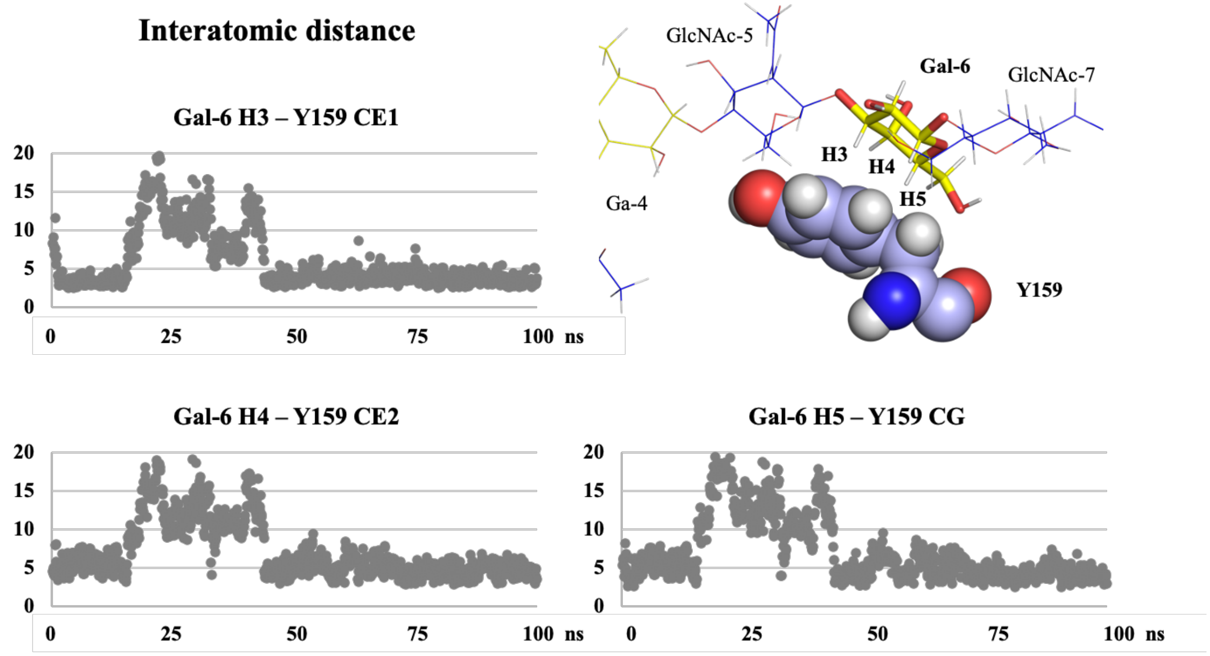

Supplementary Figure 10. Details of the CH-π interactions for Gal-6 residue as determined by measuring the distance among the Gal-6 H3, H4 and H5 and the Y159 aromatic ring along the MD simulation.

References

1 Moremen, K. W., Ramiah, A., Stuart, M. *et al.* Expression system for structural and functional studies of human glycosylation enzymes. *Nat. Chem. Biol.* **14**, 156-162, (2018).

2 McArthur, J. B., Yu, H., Zeng, J. & Chen, X. Converting *Pasteurella multocida* α2–3-sialyltransferase 1 (PmST1) to a regioselective α2–6-sialyltransferase by saturation mutagenesis and regioselective screening. *Org. Biomol. Chem.* **15**, 1700-1709, (2017).

3 Seko, A., Koketsu, M., Nishizono, M., Enoki, Y., Ibrahim, H. R., Juneja, L. R., Kim, M. & Yamamoto, T. Occurrence of a sialylglycopeptide and free sialylglycans in hen's egg yolk. *Biochim. Biophys. Acta* **1335**, 23-32, (1997).

4 Liu, L., Prudden, A. R., Capicciotti, C. J., Bosman, G. P., Yang, J.-Y., Chapla, D. G., Moremen, K. W. & Boons, G.-J. Streamlining the chemoenzymatic synthesis of complex *N*-glycans by a stop and go strategy. *Nat. Chem.* **11**, 161-169, (2018).

5 Lin, Y. P., Xiong, X., Wharton, S. A., Martin, S. R., Coombs, P. J., Vachieri, S. G., Christodoulou, E., Walker, P. A., Liu, J., Skehel, J. J., Gamblin, S. J., Hay, A. J., Daniels, R. S. & McCauley, J. W. Evolution of the receptor binding properties of the influenza A(H3N2) hemagglutinin. *Proc. Nat. Acad. Sci. U.S.A.* **109**, 21474, (2012).

6 Wu, N. C., Zost, S. J., Thompson, A. J., Oyen, D., Nycholat, C. M., McBride, R., Paulson, J. C., Hensley, S. E. & Wilson, I. A. A structural explanation for the low effectiveness of the seasonal influenza H3N2 vaccine. *PLOS Pathogens* **13**, e1006682, (2017).

7 Wu, N. C., Thompson, A. J., Xie, J., Lin, C.-W., Nycholat, C. M., Zhu, X., Lerner, R. A., Paulson, J. C. & Wilson, I. A. A complex epistatic network limits the mutational reversibility in the influenza hemagglutinin receptor-binding site. *Nat. Commun.* **9**, 1264, (2018).

8 Wu, N. C., Otwinowski, J., Thompson, A. J., Nycholat, C. M., Nourmohammad, A. & Wilson, I. A. Major antigenic site B of human influenza H3N2 viruses has an evolving local fitness landscape. *Nat. Commun.* **11**, 1233, (2020).

9 Peng, W., de Vries, R. P., Grant, O. C., Thompson, A. J., McBride, R., Tsogtbaatar, B., Lee, P. S., Razi, N., Wilson, I. A., Woods, R. J. & Paulson, J. C. Recent H3N2 viruses have evolved specificity for extended, branched human-type receptors, conferring potential for increased avidity. *Cell Host Microbe* **21**, 23-34, (2017).
