# Supplementary material for: Glycan remodeled erythrocytes facilitate antigenic characterization of recent A/H3N2 influenza viruses
Source: Nat Commun. 2021 Sep 14;12:5449. doi: 10.1038/s41467-021-25713-1 (PMC8440751; doi:10.1038/s41467-021-25713-1)
Supplement: Supplementary file 3 — Description of Additional Supplementary Information [file 41467_2021_25713_MOESM3_ESM.pdf]

Description of Additional Supplementary Files

File Name: Supplementary data 1

Description: Full glycomic data of N-glycans on unmodified fowl erythrocytes. Provided as a separate pdf file.

File Name: Supplementary data 2

Description: Full glycomic data of N-glycans on 2,6-Sia Poly-LN fowl erythrocytes. Provided as a separate pdf file.

File Name: Source data

Description: All source data. Provided as a separate xlsx file.
