# Supplementary material for: Glycan remodeled erythrocytes facilitate antigenic characterization of recent A/H3N2 influenza viruses
Source: Nat Commun. 2021 Sep 14;12:5449. doi: 10.1038/s41467-021-25713-1 (PMC8440751; doi:10.1038/s41467-021-25713-1)
Supplement: Supplementary file 4 — Supplementary data 1 [file 41467_2021_25713_MOESM4_ESM.pdf]

Data S1. Structural assignment and relative abundance of N-glycans on unmodified fowl erythrocytes.

Chicken unmodified

Proposed structure	Relative abundance [%]	mass [Da]	rt [min]	Hex	HexNAc	Fuc	NeuAc	NeuGc
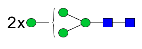	15,432	1355,48619	13,654	5	2	0	0	0
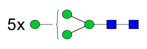	7,871	1841,64466	17,369	8	2	0	0	0
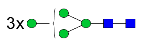	6,358	1517,53901	14,986	6	2	0	0	0
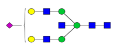	4,939	2255,81973	16,964	5	5	0	1	0
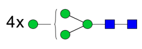	4,912	1679,59184	16,283	7	2	0	0	0
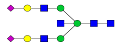	4,619	2546,91514	17,996	5	5	0	2	0
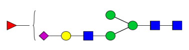	4,049	1833,66607	14,778	4	3	1	1	0
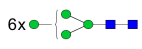	3,852	2003,69748	18,294	9	2	0	0	0
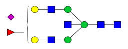	3,547	2401,87763	17,17	5	5	1	1	0
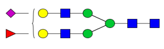	3,545	2198,79826	16,341	5	4	1	1	0
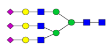	3,404	3000,06338	19,345	6	5	0	3	0
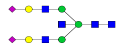	2,733	2546,91514	18,682	5	5	0	2	0
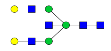	2,316	1964,72431	15,226	5	5	0	0	0
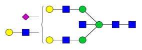	2,271	2620,95192	17,518	6	6	0	1	0
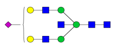	1,994	2255,81973	16,008	5	5	0	1	0
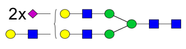	1,483	2708,96797	18,096	6	5	0	2	0
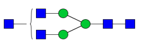	1,482	1640,61866	13,232	3	5	0	0	0
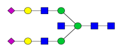	1,290	2546,91514	16,859	5	5	0	2	0
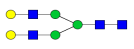	1,167	1761,64494	14,475	5	4	0	0	0
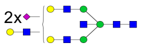	1,137	2912,04734	18,51	6	6	0	2	0
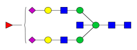	1,106	2692,97305	18,178	5	5	1	2	0
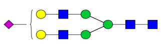	1,043	2052,74035	16,121	5	4	0	1	0
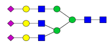	0,999	3000,06338	20,008	6	5	0	3	0
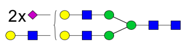	0,954	2708,96797	18,622	6	5	0	2	0
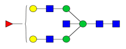	0,945	2110,78222	15,386	5	5	1	0	0
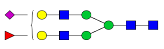	0,942	2198,79826	15,406	5	4	1	1	0
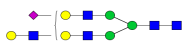	0,886	2417,87255	17,618	6	5	0	1	0
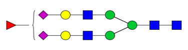	0,866	2489,89368	17,47	5	4	1	2	0
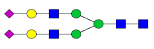	0,863	2343,83577	17,977	5	4	0	2	0

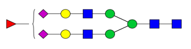	0,808	2489,89368	16,584	5	4	1	2	0
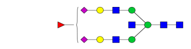	0,796	2692,97305	18,849	5	5	1	2	0
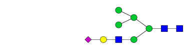	0,723	2011,7138	15,926	6	3	0	1	0
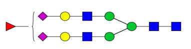	0,619	2489,89368	18,16	5	4	1	2	0
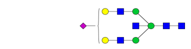	0,607	2255,81973	15,857	5	5	0	1	0
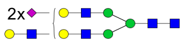	0,576	2708,96797	18,082	6	5	0	2	0
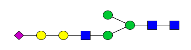	0,547	1849,66098	14,777	5	3	0	1	0
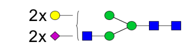	0,509	2140,7564	18,167	5	3	0	2	0
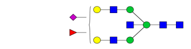	0,496	2401,87763	16,178	5	5	1	1	0
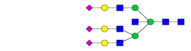	0,451	3203,14275	20,462	6	6	0	3	0
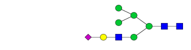	0,430	2011,7138	16,819	6	3	0	1	0
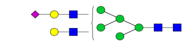	0,422	2376,846	18,505	7	4	0	1	0
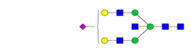	0,404	2255,81973	16,974	5	5	0	1	0
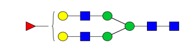	0,368	1907,70285	14,718	5	4	1	0	0
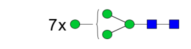	0,361	2165,75031	19,126	10	2	0	0	0
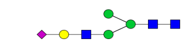	0,358	1687,60816	14,569	4	3	0	1	0
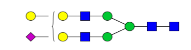	0,281	2214,79318	17,37	6	4	0	1	0
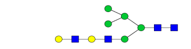	0,261	2085,75058	16,791	7	4	0	0	0
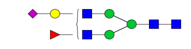	0,260	2036,74544	15,469	4	4	1	1	0
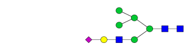	0,235	2011,7138	16,989	6	3	0	1	0
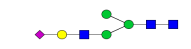	0,226	1687,60816	15,095	4	3	0	1	0
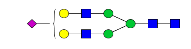	0,218	2052,74035	15,226	5	4	0	1	0
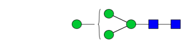	0,215	1193,43337	11,784	4	2	0	0	0
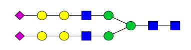	0,214	2667,94142	18,781	7	4	0	2	0
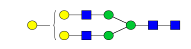	0,202	1923,69776	15,599	6	4	0	0	0
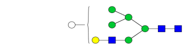	0,200	1882,67121	8,886	7	3	0	0	0
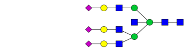	0,182	3203,14275	19,405	6	6	0	3	0
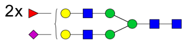	0,176	2344,85617	17,233	5	4	2	1	0
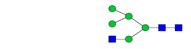	0,133	1558,56556	14,051	5	3	0	0	0
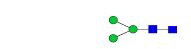	0,130	1031,38054	10,145	3	2	0	0	0
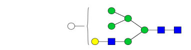	0,125	1882,67121	8,339	7	3	0	0	0
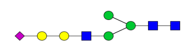	0,114	1849,66098	16,074	5	3	0	1	0

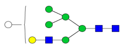	0,108	1882,67121	9,065	7	3	0	0	0
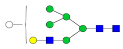	0,107	1882,67121	8,539	7	3	0	0	0
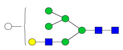	0,103	1882,67121	8,778	7	3	0	0	0
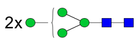	0,098	1355,48619	18,501	5	2	0	0	0
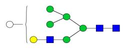	0,091	1882,67121	8,258	7	3	0	0	0
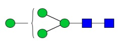	0,090	1193,43337	11,921	4	2	0	0	0
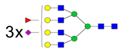	0,080	3511,25349	20,472	7	6	1	3	0
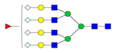	0,076	3866,32844	9,005	7	6	1	0	4
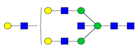	0,071	2329,8565	16,988	6	6	0	0	0
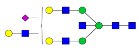	0,070	2620,95192	18,719	6	6	0	1	0
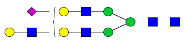	0,070	2417,87255	17,263	6	5	0	1	0
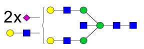	0,070	2912,04734	18,111	6	6	0	2	0
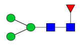	0,057	1177,43845	10,645	3	2	1	0	0
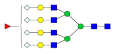	0,046	3866,32844	8,887	7	6	1	0	4
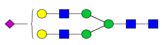	0,036	2052,74035	16,413	5	4	0	1	0
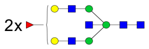	0,023	2256,84013	8,263	5	5	2	0	0
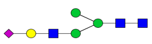	0,022	1687,60816	13,688	4	3	0	1	0
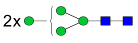	0,021	1355,48619	18,733	5	2	0	0	0
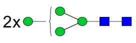	0,020	1355,48619	18,839	5	2	0	0	0
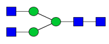	0,019	1437,53929	12,372	3	4	0	0	0
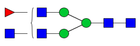	0,019	1786,67657	8,853	3	5	1	0	0
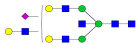	0,018	2620,95192	18,549	6	6	0	1	0
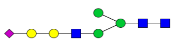	0,012	1849,66098	9,341	5	3	0	1	0
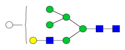	0,012	1882,67121	9,254	7	3	0	0	0
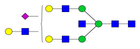	0,011	2620,95192	12,513	6	6	0	1	0

Turkey unmodified

Proposed structure	Relative abundance [%]	mass [Da]	rt [min]	Hex	HexNAc	Fuc	NeuAc	NeuGc
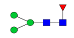	6,934	1177,438453	13,655	3	2	1	0	0
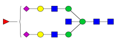	5,812	2692,97305	19,018	5	5	1	2	0
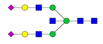	3,858	2546,915141	19,561	5	5	0	2	0
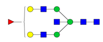	3,670	2110,782217	8,574	5	5	1	0	0
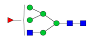	3,213	1704,623472	15,253	5	3	1	0	0
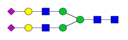	2,696	2343,835769	16,875	5	4	0	2	0
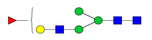	2,376	1542,570649	24,931	4	3	1	0	0
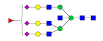	2,263	3349,200662	19,971	6	6	1	3	0
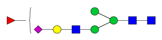	2,262	1833,666065	16,814	4	3	1	1	0
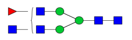	1,938	1786,67657	16,527	3	5	1	0	0
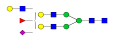	1,856	2563,930457	19,654	6	5	1	1	0
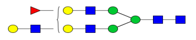	1,807	2272,83504	16,335	6	5	1	0	0
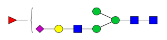	1,752	1833,666065	17,178	4	3	1	1	0
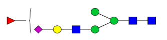	1,707	1833,666065	15,065	4	3	1	1	0
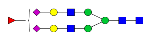	1,687	2489,893678	16,378	5	4	1	2	0
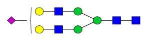	1,659	2052,740352	24,916	5	4	0	1	0
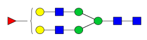	1,624	1948,729394	17,432	4	5	1	0	0
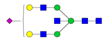	1,343	2255,819725	16,526	5	5	0	1	0
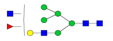	1,275	2069,755668	14,412	6	4	1	0	0
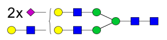	1,259	2708,967965	21,243	6	5	0	2	0
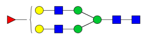	1,219	1907,702845	16,649	5	4	1	0	0
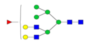	1,209	2231,808491	19,358	7	4	1	0	0
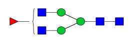	1,187	1583,597198	24,738	3	4	1	0	0
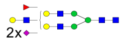	1,178	2855,025873	18,554	6	5	1	2	0
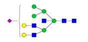	1,133	2434,887864	14,631	7	5	1	0	0
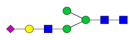	1,116	1687,608156	11,58	4	3	0	1	0
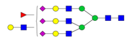	1,029	3511,253486	20,458	7	6	1	3	0
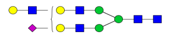	1,001	2417,872548	16,514	6	5	0	1	0
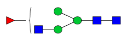	1,001	1380,517825	19,35	3	3	1	0	0
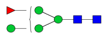	0,990	1339,491276	11,365	4	2	1	0	0
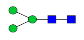	0,990	1031,380544	10,847	3	2	0	0	0
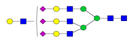	0,983	3365,195577	23,142	7	6	0	3	0
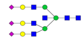	0,927	3203,142754	19,322	6	6	0	3	0
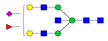	0,893	2401,877634	17,863	5	5	1	1	0
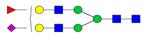	0,890	2198,798261	8,091	5	4	1	1	0

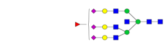	0,852	3349,200662	20,298	6	6	1	3	0
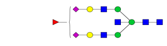	0,837	2692,97305	18,797	5	5	1	2	0
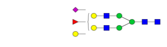	0,830	2360,851084	18,693	6	4	1	1	0
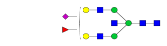	0,682	2401,877634	15,935	5	5	1	1	0
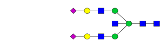	0,654	2546,915141	15,845	5	5	0	2	0
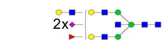	0,628	3058,105246	20,774	6	6	1	2	0
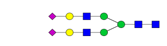	0,598	2343,835769	17,235	5	4	0	2	0
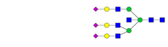	0,596	3203,142754	19,552	6	6	0	3	0
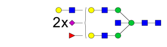	0,588	3058,105246	20,604	6	6	1	2	0
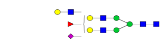	0,577	2563,930457	20,294	6	5	1	1	0
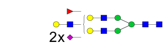	0,563	2855,025873	21,302	6	5	1	2	0
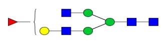	0,557	1745,650021	15,66	4	4	1	0	0
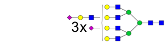	0,556	4021,42319	24,324	8	7	0	4	0
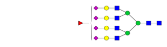	0,525	3802,348902	19,538	7	6	1	4	0
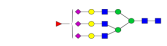	0,515	3146,12129	18,236	6	5	1	3	0
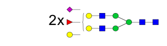	0,501	2506,908993	20,168	6	4	2	1	0
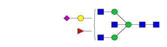	0,497	2239,82481	15,356	4	5	1	1	0
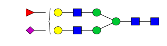	0,496	2198,798261	21,657	5	4	1	1	0
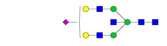	0,476	2255,819725	17,339	5	5	0	1	0
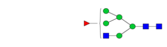	0,462	1995,718888	15,626	5	3	1	1	0
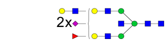	0,456	3058,105246	19,152	6	6	1	2	0
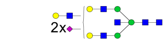	0,455	2912,047337	20,874	6	6	0	2	0
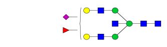	0,454	2401,877634	17,051	5	5	1	1	0
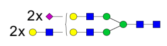	0,452	3074,100161	19,56	7	6	0	2	0
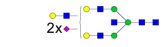	0,437	2912,047337	18,447	6	6	0	2	0
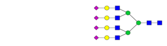	0,435	3656,290994	21,876	7	6	0	4	0
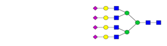	0,431	3656,290994	19,133	7	6	0	4	0
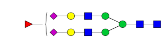	0,422	2489,893678	17,165	5	4	1	2	0
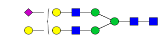	0,421	2214,793176	24,507	6	4	0	1	0
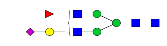	0,419	2036,745438	16,514	4	4	1	1	0
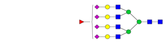	0,397	3802,348902	22,835	7	6	1	4	0
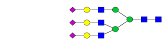	0,384	3000,063381	21,695	6	5	0	3	0
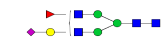	0,373	2036,745438	18,532	4	4	1	1	0
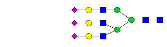	0,366	3000,063381	19,755	6	5	0	3	0
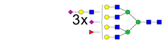	0,365	4167,481098	23,528	8	7	1	4	0
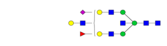	0,363	2767,009829	19,548	6	6	1	1	0
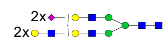	0,339	3074,100161	20,164	7	6	0	2	0

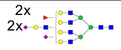	0,335	4241,517878	23,398	9	8	1	3	0
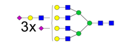	0,331	4183,475982	23,503	9	7	0	4	0
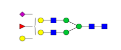	0,325	2360,851084	17,543	6	4	1	1	0
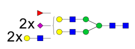	0,317	3220,158069	18,159	7	6	1	2	0
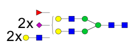	0,313	3220,158069	19,456	7	6	1	2	0
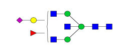	0,311	2239,82481	18,553	4	5	1	1	0
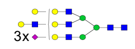	0,309	3527,248401	20,066	8	6	0	3	0
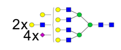	0,307	4548,608209	23,854	10	8	0	4	0
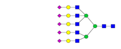	0,306	4312,518606	21,545	8	7	0	5	0
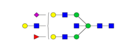	0,286	2767,009829	20,859	6	6	1	1	0
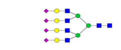	0,284	3656,290994	23,503	7	6	0	4	0
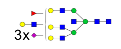	0,284	3714,332858	19,035	7	7	1	3	0
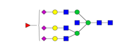	0,271	3349,200662	21,632	6	6	1	3	0
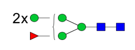	0,264	1501,544099	12,581	5	2	1	0	0
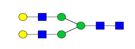	0,264	1761,644936	14,996	5	4	0	0	0
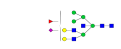	0,260	2725,98328	20,953	7	5	1	1	0
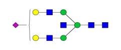	0,257	2255,819725	14,971	5	5	0	1	0
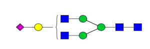	0,256	1890,687529	15,34	4	4	0	1	0
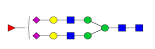	0,250	2489,893678	21,075	5	4	1	2	0
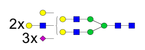	0,247	3673,306309	22,374	8	6	1	3	0
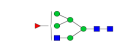	0,239	1995,718888	16,1	5	3	1	1	0
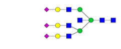	0,237	3203,142754	19,642	6	6	0	3	0
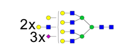	0,234	4257,512792	23,046	10	8	0	3	0
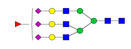	0,232	3146,12129	18,06	6	5	1	3	0
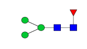	0,208	1177,438453	12,854	3	2	1	0	0
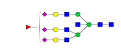	0,207	3349,200662	21,554	6	6	1	3	0
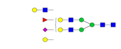	0,206	3091,115476	19,6	8	6	1	1	0
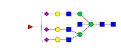	0,202	3349,200662	22,602	6	6	1	3	0
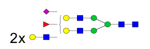	0,193	2929,062653	21,598	7	6	1	1	0
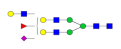	0,189	2563,930457	18,98	6	5	1	1	0
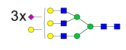	0,182	3162,116205	20,083	7	5	0	3	0
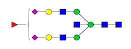	0,179	2692,97305	21,315	5	5	1	2	0
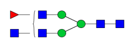	0,171	1786,67657	13,924	3	5	1	0	0
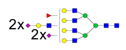	0,169	4532,613294	23,031	9	8	1	4	0
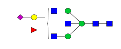	0,168	2239,82481	18,728	4	5	1	1	0
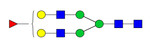	0,161	1907,702845	17,912	5	4	1	0	0
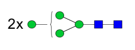	0,154	1355,486191	13,734	5	2	0	0	0

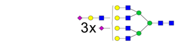	0,144	4183,475982	24,441	9	7	0	4	0
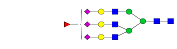	0,141	3146,12129	19,017	6	5	1	3	0
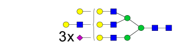	0,138	3527,248401	20,794	8	6	0	3	0
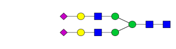	0,136	2343,835769	16,712	5	4	0	2	0
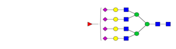	0,134	3802,348902	18,68	7	6	1	4	0
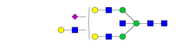	0,131	2620,951921	17,63	6	6	0	1	0
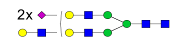	0,126	2708,967965	21,983	6	5	0	2	0
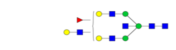	0,122	2475,914413	18,697	6	6	1	0	0
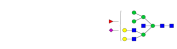	0,114	2725,98328	19,672	7	5	1	1	0
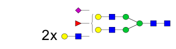	0,111	2929,062653	22,464	7	6	1	1	0
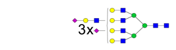	0,109	4021,42319	24,71	8	7	0	4	0
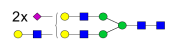	0,106	2708,967965	19,628	6	5	0	2	0
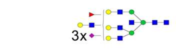	0,105	3714,332858	20,924	7	7	1	3	0
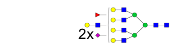	0,104	3585,290265	23,677	8	7	1	2	0
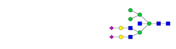	0,102	2871,020788	20,629	7	5	0	2	0
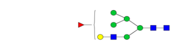	0,100	1866,676295	17,355	6	3	1	0	0
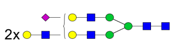	0,097	2783,004744	21,956	7	6	0	1	0
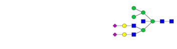	0,094	2871,020788	20,869	7	5	0	2	0
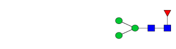	0,092	1177,438453	12,028	3	2	1	0	0
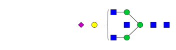	0,092	2093,766901	15,465	4	5	0	1	0
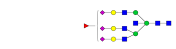	0,088	3349,200662	20,041	6	6	1	3	0
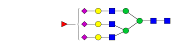	0,086	3146,12129	17,483	6	5	1	3	0
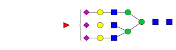	0,082	3146,12129	20,563	6	5	1	3	0
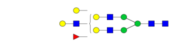	0,078	2596,940687	19,054	8	5	1	0	0
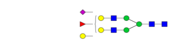	0,077	2360,851084	18,543	6	4	1	1	0
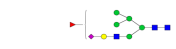	0,076	2157,771712	16,319	6	3	1	1	0
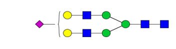	0,075	2052,740352	18,385	5	4	0	1	0
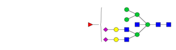	0,075	3017,078697	21,915	7	5	1	2	0
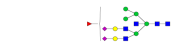	0,073	3017,078697	20,403	7	5	1	2	0
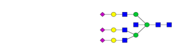	0,066	3203,142754	21,572	6	6	0	3	0
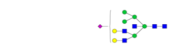	0,065	2579,925372	19,157	7	5	0	1	0
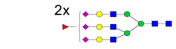	0,065	3146,12129	19,489	6	5	1	3	0
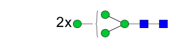	0,065	1355,486191	10,471	5	2	0	0	0
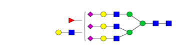	0,065	3511,253486	23,997	7	6	1	3	0
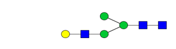	0,064	1396,51274	12,283	4	3	0	0	0
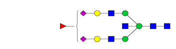	0,063	2692,97305	18,111	5	5	1	2	0
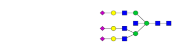	0,061	3203,142754	17,698	6	6	0	3	0

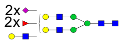	0,058	3001,083782	17,713	6	5	2	2	0
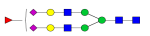	0,057	2489,893678	19,81	5	4	1	2	0
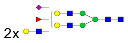	0,051	2929,062653	18,425	7	6	1	1	0
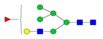	0,051	1866,676295	15,74	6	3	1	0	0
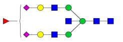	0,051	2692,97305	16,217	5	5	1	2	0
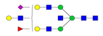	0,050	2767,009829	20,561	6	6	1	1	0
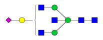	0,050	2093,766901	17,544	4	5	0	1	0
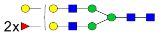	0,049	2215,813577	8,325	6	4	2	0	0
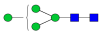	0,049	1193,433367	15,45	4	2	0	0	0
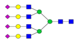	0,044	3656,290994	23,673	7	6	0	4	0
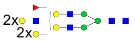	0,043	2962,072883	19,047	9	6	1	0	0
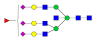	0,043	3349,200662	22,616	6	6	1	3	0
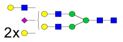	0,042	2741,978195	20,149	8	5	0	1	0
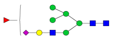	0,042	2157,771712	24,639	6	3	1	1	0
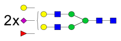	0,039	2651,946501	17,469	6	4	1	2	0
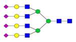	0,038	3656,290994	19,234	7	6	0	4	0
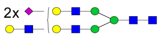	0,038	2708,967965	21,233	6	5	0	2	0
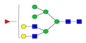	0,038	2231,808491	19,209	7	4	1	0	0
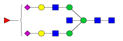	0,037	2692,97305	19,776	5	5	1	2	0
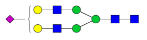	0,035	2052,740352	24,794	5	4	0	1	0
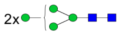	0,035	1355,486191	19,072	5	2	0	0	0
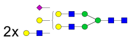	0,035	2945,057568	19,358	8	6	0	1	0
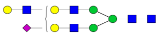	0,034	2417,872548	18,052	6	5	0	1	0
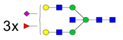	0,034	2693,993451	17,973	5	5	3	1	0
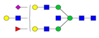	0,028	2767,009829	17,683	6	6	1	1	0
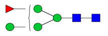	0,026	1339,491276	14,065	4	2	1	0	0
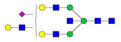	0,026	2620,951921	15,657	6	6	0	1	0
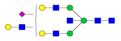	0,024	2620,951921	19,812	6	6	0	1	0
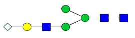	0,024	1703,60304	20,295	4	3	0	0	1
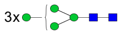	0,023	1517,539014	12,555	6	2	0	0	0
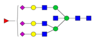	0,022	3349,200662	23,161	6	6	1	3	0
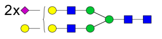	0,021	2505,888592	17,844	6	4	0	2	0
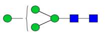	0,020	1193,433367	14,025	4	2	0	0	0
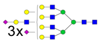	0,019	4183,475982	24,766	9	7	0	4	0
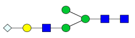	0,019	1703,60304	21,123	4	3	0	0	1
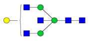	0,019	1802,671485	17,217	4	5	0	0	0
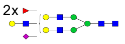	0,018	2709,988366	17,053	6	5	2	1	0

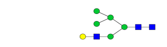	0,018	1720,618387	14,242	6	3	0	0	0
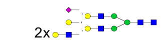	0,017	2945,057568	16,757	8	6	0	1	0
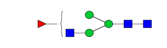	0,017	1380,517825	12,954	3	3	1	0	0
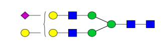	0,017	2214,793176	13,352	6	4	0	1	0
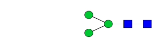	0,017	1031,380544	14,073	3	2	0	0	0
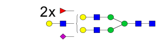	0,016	2709,988366	19,19	6	5	2	1	0
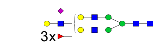	0,014	2856,046275	21,931	6	5	3	1	0
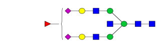	0,013	2692,97305	19,909	5	5	1	2	0
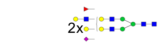	0,013	2888,036104	19,672	8	5	1	1	0
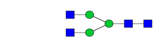	0,013	1437,539289	11,368	3	4	0	0	0
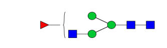	0,012	1380,517825	15,244	3	3	1	0	0
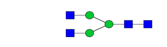	0,012	1437,539289	10,964	3	4	0	0	0
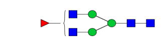	0,012	1583,597198	23,98	3	4	1	0	0
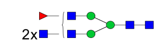	0,009	1989,755943	12,834	3	6	1	0	0
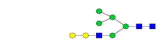	0,009	1882,67121	15,762	7	3	0	0	0
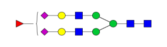	0,008	2489,893678	14,744	5	4	1	2	0
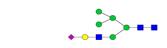	0,008	2011,713803	16,32	6	3	0	1	0
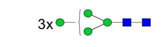	0,008	1517,539014	13,451	6	2	0	0	0
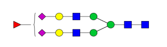	0,008	2489,893678	18,934	5	4	1	2	0
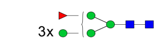	0,007	1663,596923	12,527	6	2	1	0	0
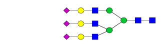	0,007	3000,063381	20,414	6	5	0	3	0
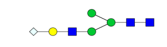	0,007	1703,60304	13,13	4	3	0	0	1
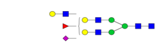	0,006	2563,930457	19,71	6	5	1	1	0
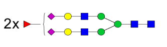	0,006	2635,951586	19,806	5	4	2	2	0
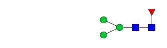	0,005	1177,438453	12,545	3	2	1	0	0
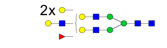	0,005	2596,940687	16,989	8	5	1	0	0
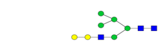	0,005	1882,67121	16,426	7	3	0	0	0
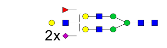	0,005	2855,025873	22,006	6	5	1	2	0
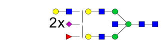	0,005	3058,105246	20,865	6	6	1	2	0
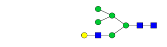	0,005	1720,618387	14,535	6	3	0	0	0
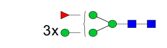	0,005	1663,596923	14,367	6	2	1	0	0
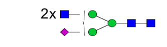	0,004	1728,634705	14,592	3	4	0	1	0
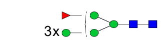	0,004	1663,596923	24,051	6	2	1	0	0
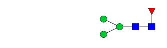	0,003	1177,438453	10,416	3	2	1	0	0
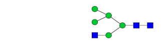	0,003	1558,565563	24,079	5	3	0	0	0
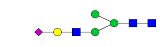	0,003	1687,608156	23,606	4	3	0	1	0
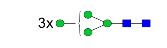	0,002	1517,539014	14,166	6	2	0	0	0

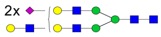	0,002	2708,967965	20,287	6	5	0	2	0
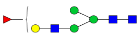	0,002	1542,570649	24,956	4	3	1	0	0
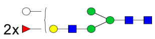	0,002	1850,681381	15,98	5	3	2	0	0
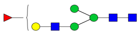	0,002	1542,570649	11,896	4	3	1	0	0
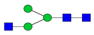	0,001	1234,459916	16,392	3	3	0	0	0
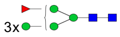	0,001	1663,596923	8,26	6	2	1	0	0
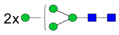	0,001	1355,486191	12,551	5	2	0	0	0
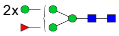	0,001	1501,544099	11,497	5	2	1	0	0
