# Supplementary material for: Glycan remodeled erythrocytes facilitate antigenic characterization of recent A/H3N2 influenza viruses
Source: Nat Commun. 2021 Sep 14;12:5449. doi: 10.1038/s41467-021-25713-1 (PMC8440751; doi:10.1038/s41467-021-25713-1)
Supplement: Supplementary file 5 — Supplementary data 2 [file 41467_2021_25713_MOESM5_ESM.pdf]

Data S2. Structural assignment and relative abundance of N-glycans on 2,6-Sia Poly-LacNac fowl erythrocytes.

Chicken 2,6-Sia Poly-LacNAc

Proposed structure	Relative abundance [%]	mass [Da]	rt [min]	Hex	HexNAc	Fuc	NeuAc	NeuGc
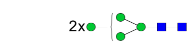	9,283	1355,48619	13,972	5	2	0	0	0
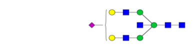	6,341	2255,81972	17,482	5	5	0	1	0
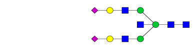	4,577	2546,91514	19,133	5	5	0	2	0
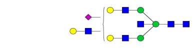	3,986	2620,95192	19,131	6	6	0	1	0
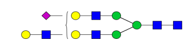	3,753	2417,87255	18,385	6	5	0	1	0
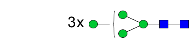	3,691	1517,53901	15,323	6	2	0	0	0
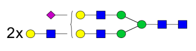	3,550	2783,00474	19,424	7	6	0	1	0
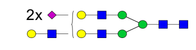	2,787	2708,96796	19,937	6	5	0	2	0
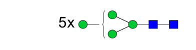	2,354	1841,64466	17,795	8	2	0	0	0
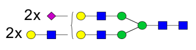	2,282	3074,10016	20,899	7	6	0	2	0
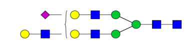	2,232	2417,87255	18,121	6	5	0	1	0
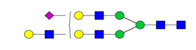	2,189	2417,87252	18,374	6	5	0	1	0
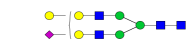	1,829	2214,79318	17,852	6	4	0	1	0
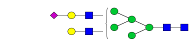	1,829	2376,846	18,929	7	4	0	1	0
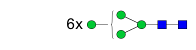	1,789	2003,69748	18,715	9	2	0	0	0
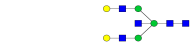	1,748	1964,72431	15,579	5	5	0	0	0
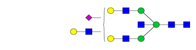	1,633	2620,95192	18,826	6	6	0	1	0
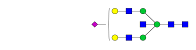	1,545	2255,81972	17,219	5	5	0	1	0
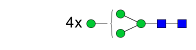	1,494	1679,59184	16,719	7	2	0	0	0
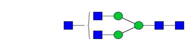	1,484	1640,61866	13,587	3	5	0	0	0
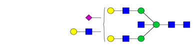	1,482	2620,95192	18,464	6	6	0	1	0
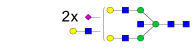	1,386	2912,04734	20,187	6	6	0	2	0
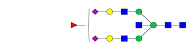	1,341	2692,97305	19,287	5	5	1	2	0
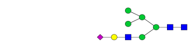	1,158	2011,71377	17,384	6	3	0	1	0
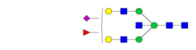	1,094	2401,87763	17,675	5	5	1	1	0
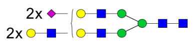	1,083	3074,10016	20,962	7	6	0	2	0
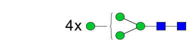	1,025	1679,59184	16,51	7	2	0	0	0
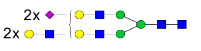	0,923	3074,10016	20,751	7	6	0	2	0
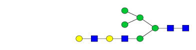	0,776	2085,75058	17,2	7	4	0	0	0
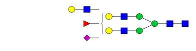	0,753	2563,93046	18,523	6	5	1	1	0
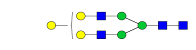	0,731	1923,69776	15,986	6	4	0	0	0
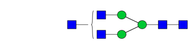	0,722	1640,61866	13,582	3	5	0	0	0

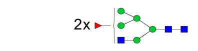	0,717	1850,68138	15,345	5	3	2	0	0
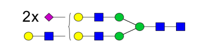	0,678	2708,96796	19,945	6	5	0	2	0
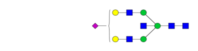	0,669	2255,81972	17,483	5	5	0	1	0
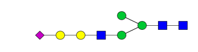	0,635	1849,66098	16,258	5	3	0	1	0
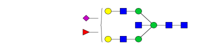	0,626	2401,87763	17,407	5	5	1	1	0
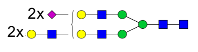	0,617	3074,10016	20,806	7	6	0	2	0
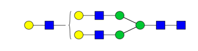	0,592	2126,77713	16,81	6	5	0	0	0
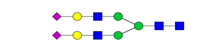	0,582	2343,83577	18,495	5	4	0	2	0
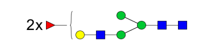	0,541	1688,62856	14,068	4	3	2	0	0
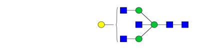	0,520	1802,67148	14,545	4	5	0	0	0
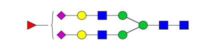	0,501	2489,89368	18,676	5	4	1	2	0
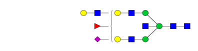	0,489	2767,00983	18,978	6	6	1	1	0
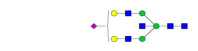	0,474	2255,81972	17,211	5	5	0	1	0
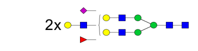	0,474	2929,06265	19,508	7	6	1	1	0
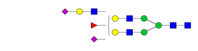	0,462	2855,02587	20,066	6	5	1	2	0
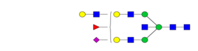	0,456	2767,00983	19,249	6	6	1	1	0
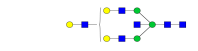	0,418	2329,8565	17,006	6	6	0	0	0
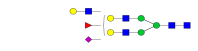	0,413	2563,93046	18,276	6	5	1	1	0
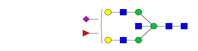	0,407	2401,87763	17,682	5	5	1	1	0
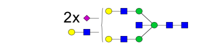	0,392	2912,04734	20,526	6	6	0	2	0
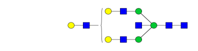	0,370	2329,8565	16,958	6	6	0	0	0
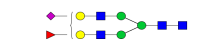	0,361	2198,79826	16,843	5	4	1	1	0
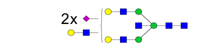	0,360	2912,04734	20,531	6	6	0	2	0
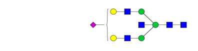	0,352	2255,81972	16,512	5	5	0	1	0
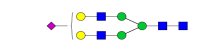	0,352	2052,74035	16,636	5	4	0	1	0
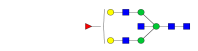	0,342	2110,78222	15,799	5	5	1	0	0
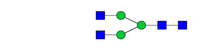	0,338	1437,53929	12,738	3	4	0	0	0
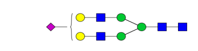	0,327	2052,74032	16,632	5	4	0	1	0
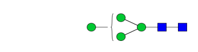	0,314	1193,43337	12,28	4	2	0	0	0
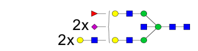	0,297	3423,23744	21,579	7	7	1	2	0
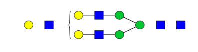	0,292	2126,77713	16,321	6	5	0	0	0
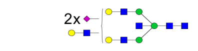	0,281	2912,04734	19,778	6	6	0	2	0
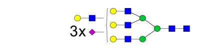	0,280	3365,19558	22,189	7	6	0	3	0
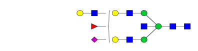	0,280	2767,00983	18,598	6	6	1	1	0
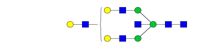	0,278	2329,8565	17,405	6	6	0	0	0

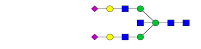	0,258	2546,91514	18,491	5	5	0	2	0
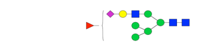	0,245	2157,77171	17,582	6	3	1	1	0
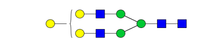	0,244	1923,69776	16,307	6	4	0	0	0
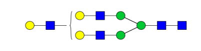	0,243	2126,77713	16,314	6	5	0	0	0
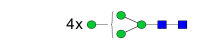	0,240	1679,59184	16,711	7	2	0	0	0
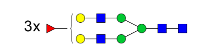	0,239	2199,81866	16,614	5	4	3	0	0
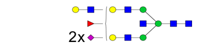	0,234	3058,10525	20,292	6	6	1	2	0
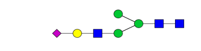	0,228	1687,60816	15,02	4	3	0	1	0
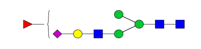	0,227	1833,66607	15,277	4	3	1	1	0
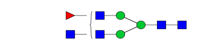	0,226	1786,67657	13,832	3	5	1	0	0
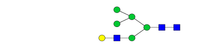	0,225	1720,61839	15,633	6	3	0	0	0
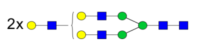	0,222	2491,90933	18,116	7	6	0	0	0
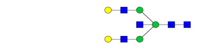	0,221	1964,72431	15,576	5	5	0	0	0
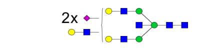	0,220	2912,04734	19,778	6	6	0	2	0
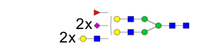	0,206	3220,15807	20,884	7	6	1	2	0
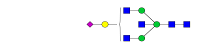	0,201	2093,7669	16,716	4	5	0	1	0
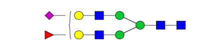	0,193	2198,79826	16,851	5	4	1	1	0
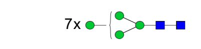	0,192	2165,75031	19,504	10	2	0	0	0
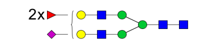	0,184	2344,85617	17,569	5	4	2	1	0
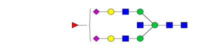	0,171	2692,97305	19,296	5	5	1	2	0
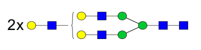	0,169	2491,90933	17,768	7	6	0	0	0
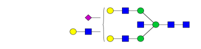	0,164	2620,95192	19,131	6	6	0	1	0
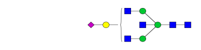	0,157	2093,7669	15,576	4	5	0	1	0
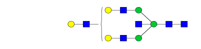	0,157	2329,8565	17,413	6	6	0	0	0
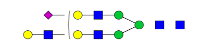	0,152	2417,87252	18,388	6	5	0	1	0
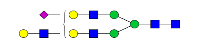	0,152	2417,87255	18,388	6	5	0	1	0
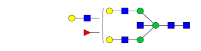	0,151	2475,91441	17,182	6	6	1	0	0
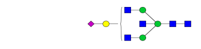	0,145	2093,7669	16,302	4	5	0	1	0
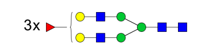	0,144	2199,81866	16,614	5	4	3	0	0
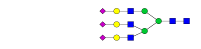	0,142	3000,06338	21,495	6	5	0	3	0
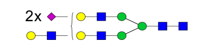	0,137	2708,96796	19,694	6	5	0	2	0
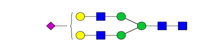	0,136	2052,74035	16,751	5	4	0	1	0
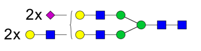	0,135	3074,10016	20,913	7	6	0	2	0
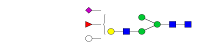	0,129	1995,71889	16,492	5	3	1	1	0
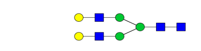	0,129	1761,64494	15,563	5	4	0	0	0

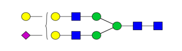	0,122	2214,79314	18,367	6	4	0	1	0
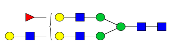	0,115	2272,83504	16,536	6	5	1	0	0
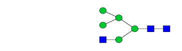	0,113	1558,56556	14,316	5	3	0	0	0
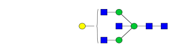	0,112	1802,67148	14,527	4	5	0	0	0
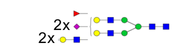	0,112	3220,15807	20,771	7	6	1	2	0
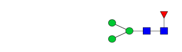	0,111	1177,43845	10,828	3	2	1	0	0
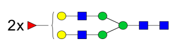	0,110	2053,76075	15,853	5	4	2	0	0
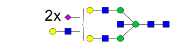	0,109	2912,04734	20,179	6	6	0	2	0
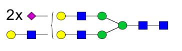	0,107	2708,96796	19,427	6	5	0	2	0
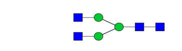	0,105	1396,51274	13,082	4	3	0	0	0
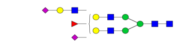	0,104	2855,02587	19,826	6	5	1	2	0
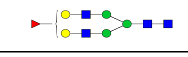	0,103	1907,70284	15,078	5	4	1	0	0
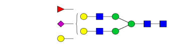	0,101	2360,85105	18,06	6	4	1	1	0
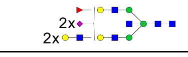	0,093	3423,23744	21,034	7	7	1	2	0
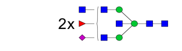	0,089	2426,90927	21,624	3	6	2	1	0
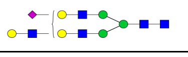	0,088	2417,87252	17,162	6	5	0	1	0
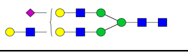	0,088	2417,87255	17,162	6	5	0	1	0
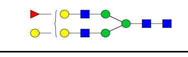	0,084	2069,75567	16,142	6	4	1	0	0
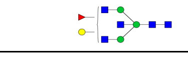	0,084	1948,72939	14,8	4	5	1	0	0
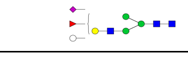	0,079	1995,71889	16,486	5	3	1	1	0
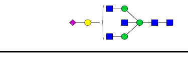	0,075	2093,7669	16,312	4	5	0	1	0
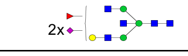	0,074	2530,92023	18,282	4	5	1	2	0
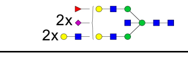	0,072	3423,23744	21,581	7	7	1	2	0
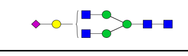	0,069	1890,68753	14,86	4	4	0	1	0
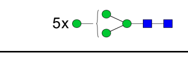	0,067	1841,64466	17,793	8	2	0	0	0
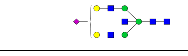	0,066	2255,81972	17,753	5	5	0	1	0
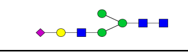	0,066	1687,60816	14,338	4	3	0	1	0
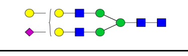	0,065	2214,79318	15,92	6	4	0	1	0
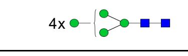	0,063	1679,59184	16,507	7	2	0	0	0
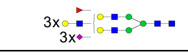	0,062	3876,38568	22,873	8	7	1	3	0
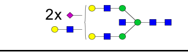	0,061	2912,04734	19,751	6	6	0	2	0
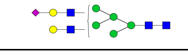	0,057	2376,846	17,066	7	4	0	1	0
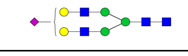	0,057	2052,74035	15,44	5	4	0	1	0
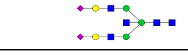	0,056	2546,91514	17,436	5	5	0	2	0
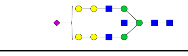	0,054	2579,92537	18,681	7	5	0	1	0

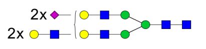	0,054	3074,10016	20,27	7	6	0	2	0
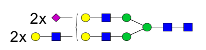	0,053	3074,10016	20,262	7	6	0	2	0
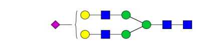	0,051	2052,74035	15,689	5	4	0	1	0
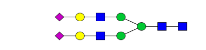	0,051	2343,83577	17,843	5	4	0	2	0
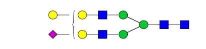	0,050	2214,79318	16,677	6	4	0	1	0
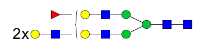	0,050	2637,96724	17,937	7	6	1	0	0
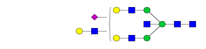	0,049	2620,95192	18,026	6	6	0	1	0
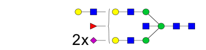	0,049	3058,10525	19,903	6	6	1	2	0
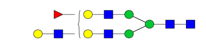	0,049	2272,83504	16,978	6	5	1	0	0
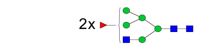	0,048	1850,68138	15,382	5	3	2	0	0
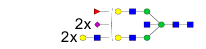	0,047	3423,23744	21,403	7	7	1	2	0
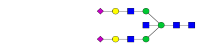	0,044	2546,91514	18,502	5	5	0	2	0
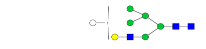	0,044	1882,67121	8,934	7	3	0	0	0
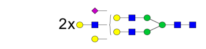	0,043	2741,97819	19,615	8	5	0	1	0
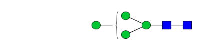	0,042	1193,43337	12,059	4	2	0	0	0
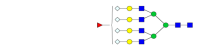	0,042	3866,32844	8,973	7	6	1	0	4
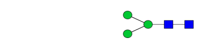	0,042	1031,38054	10,021	3	2	0	0	0
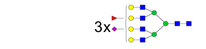	0,040	3511,25349	22,193	7	6	1	3	0
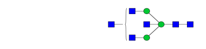	0,040	1843,69803	14,637	3	6	0	0	0
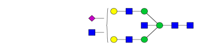	0,037	2458,8991	18,025	5	6	0	1	0
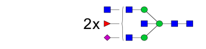	0,036	2426,90927	20,987	3	6	2	1	0
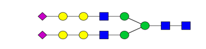	0,036	2667,94142	19,236	7	4	0	2	0
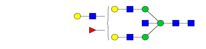	0,034	2475,91441	17,592	6	6	1	0	0
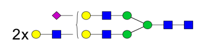	0,034	2783,00474	19,748	7	6	0	1	0
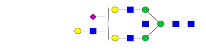	0,033	2620,95192	17,668	6	6	0	1	0
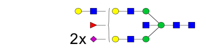	0,033	3058,10525	20,307	6	6	1	2	0
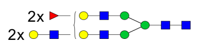	0,033	2784,02515	19,04	7	6	2	0	0
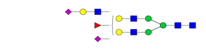	0,033	2855,02587	20,08	6	5	1	2	0
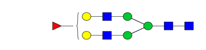	0,033	1907,70284	14,985	5	4	1	0	0
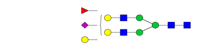	0,031	2360,85108	17,638	6	4	1	1	0
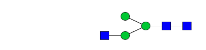	0,030	1234,45992	11,45	3	3	0	0	0
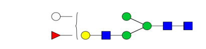	0,028	1704,62347	14,581	5	3	1	0	0
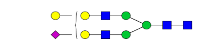	0,028	2214,79318	17,457	6	4	0	1	0
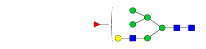	0,027	1866,6763	15,844	6	3	1	0	0
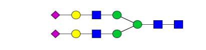	0,027	2343,83577	16,983	5	4	0	2	0

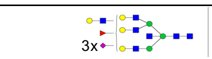	0,027	3714,33286	9,072	7	7	1	3	0
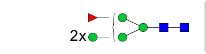	0,027	1501,5441	14,205	5	2	1	0	0
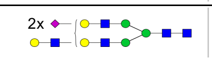	0,026	2708,96796	19,423	6	5	0	2	0
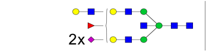	0,025	3058,10525	19,893	6	6	1	2	0
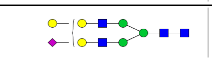	0,024	2214,79318	17,754	6	4	0	1	0
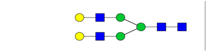	0,024	1761,64494	15,001	5	4	0	0	0
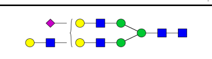	0,022	2417,87255	17,518	6	5	0	1	0
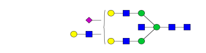	0,021	2620,95192	18,298	6	6	0	1	0
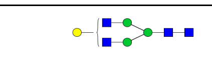	0,019	1599,59211	14,179	4	4	0	0	0
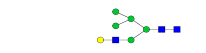	0,019	1720,61839	15,642	6	3	0	0	0
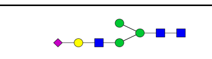	0,018	1687,60816	15,634	4	3	0	1	0
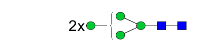	0,016	1355,48619	19,29	5	2	0	0	0
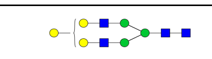	0,016	1923,69776	15,992	6	4	0	0	0
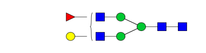	0,015	1745,65002	14,447	4	4	1	0	0
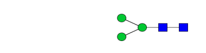	0,015	1031,38054	8,723	3	2	0	0	0
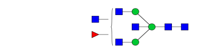	0,014	1989,75594	14,849	3	6	1	0	0
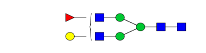	0,014	1745,65002	14,207	4	4	1	0	0
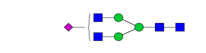	0,014	1728,63471	15,008	3	4	0	1	0
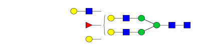	0,013	2434,88786	17,35	7	5	1	0	0
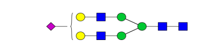	0,013	2052,74035	17,334	5	4	0	1	0
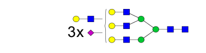	0,012	3365,19558	21,667	7	6	0	3	0
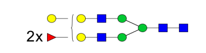	0,011	2215,81358	17,689	6	4	2	0	0
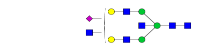	0,010	2458,8991	18,022	5	6	0	1	0
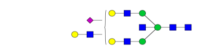	0,009	2620,95192	18,397	6	6	0	1	0
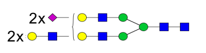	0,008	3074,10016	20,208	7	6	0	2	0
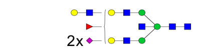	0,008	3058,10525	20,13	6	6	1	2	0
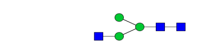	0,008	1234,45992	10,832	3	3	0	0	0
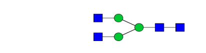	0,008	1396,51274	22,327	4	3	0	0	0
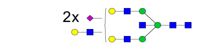	0,007	2912,04734	19,767	6	6	0	2	0
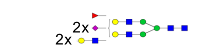	0,007	3220,15807	20,409	7	6	1	2	0
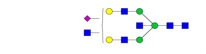	0,006	2458,8991	18,376	5	6	0	1	0
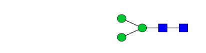	0,006	1031,38054	8,172	3	2	0	0	0
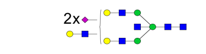	0,006	2912,04734	20,054	6	6	0	2	0
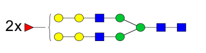	0,005	2377,8664	17,812	7	4	2	0	0
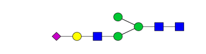	0,005	1687,60816	20,794	4	3	0	1	0

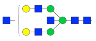	0,005	2167,80368	16,532	5	6	0	0	0
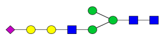	0,005	1849,66098	8,195	5	3	0	1	0
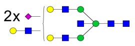	0,005	2912,04734	19,103	6	6	0	2	0
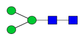	0,005	1031,38054	8,231	3	2	0	0	0
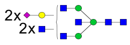	0,005	2953,07389	21,981	5	7	0	2	0
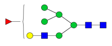	0,005	1866,6763	8,529	6	3	1	0	0
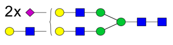	0,005	2708,96796	19,299	6	5	0	2	0
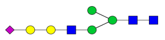	0,005	1849,66098	16,524	5	3	0	1	0
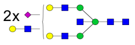	0,005	2912,04734	19,535	6	6	0	2	0
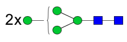	0,004	1355,48619	18,765	5	2	0	0	0
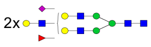	0,003	2929,06265	19,514	7	6	1	1	0
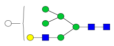	0,003	1882,67121	17,1	7	3	0	0	0
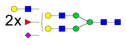	0,002	2709,98837	18,546	6	5	2	1	0
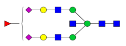	0,002	2692,97305	18,03	5	5	1	2	0
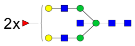	0,002	2256,84013	8,809	5	5	2	0	0
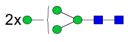	0,002	1355,48619	18,889	5	2	0	0	0
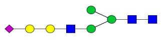	0,002	1849,66098	8,312	5	3	0	1	0
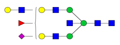	0,001	2767,00983	18,584	6	6	1	1	0
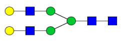	0,001	1761,64494	14,915	5	4	0	0	0
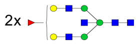	0,001	2256,84013	8,502	5	5	2	0	0
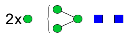	0,001	1355,48619	19,198	5	2	0	0	0
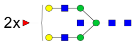	0,001	2256,84013	8,328	5	5	2	0	0
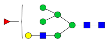	0,001	1866,6763	22,892	6	3	1	0	0
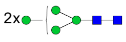	0,001	1355,48619	14,292	5	2	0	0	0
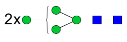	0,001	1355,48619	14,619	5	2	0	0	0
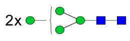	0,001	1355,48619	14,04	5	2	0	0	0

Turkey 2,6-Sia Poly-LacNac

Proposed structure	Relative abundance [%]	mass [Da]	rt [min]	Hex	HexNAc	Fuc	NeuAc	NeuGc
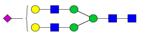	7,668	2052,740321	16,408	5	4	0	1	0
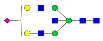	6,298	2255,819725	17,249	5	5	0	1	0
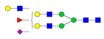	5,608	2563,930457	17,98	6	5	1	1	0
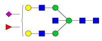	5,244	2401,877634	17,447	5	5	1	1	0
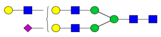	5,134	2417,872517	17,836	6	5	0	1	0
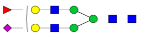	5,010	2198,798261	16,609	5	4	1	1	0
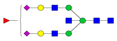	3,123	2692,97305	19,197	5	5	1	2	0
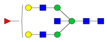	3,093	2110,782217	15,428	5	5	1	0	0
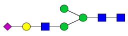	3,043	1687,608156	14,785	4	3	0	1	0
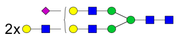	2,749	2783,004744	19,227	7	6	0	1	0
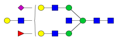	2,715	2767,009829	18,392	6	6	1	1	0
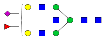	2,623	2401,877634	17,17	5	5	1	1	0
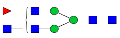	2,251	1786,67657	13,47	3	5	1	0	0
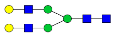	2,053	1761,644936	14,458	5	4	0	0	0
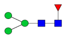	1,959	1177,438453	10,683	3	2	1	0	0
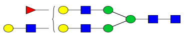	1,929	2272,83504	16,143	6	5	1	0	0
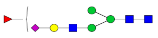	1,911	1833,666065	15,053	4	3	1	1	0
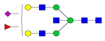	1,902	2401,877634	17,431	5	5	1	1	0
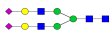	1,726	2343,835769	18,398	5	4	0	2	0
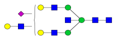	1,698	2620,951921	18,252	6	6	0	1	0
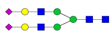	1,639	2546,915141	19,054	5	5	0	2	0
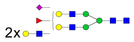	1,539	2929,062653	19,326	7	6	1	1	0
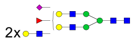	1,532	2929,062653	18,992	7	6	1	1	0
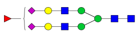	1,527	2489,893678	18,57	5	4	1	2	0
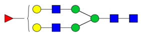	1,447	1907,702845	14,707	5	4	1	0	0
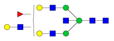	1,412	2475,914413	16,768	6	6	1	0	0
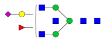	1,225	2239,82481	16,287	4	5	1	1	0
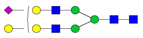	1,163	2214,793145	17,099	6	4	0	1	0
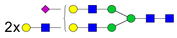	1,103	2783,004744	18,854	7	6	0	1	0
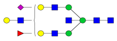	1,033	2767,009829	18,719	6	6	1	1	0
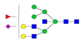	1,031	2725,98328	18,554	7	5	1	1	0
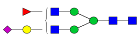	0,995	2036,745438	15,784	4	4	1	1	0
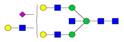	0,890	2620,951921	18,564	6	6	0	1	0
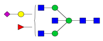	0,837	2239,82481	16,714	4	5	1	1	0
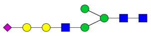	0,831	1995,718888	16,273	5	3	1	1	0

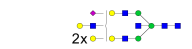		0,800	2945,057568	19,887	8	6	0	1	0
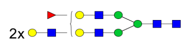		0,782	2637,967236	17,495	7	6	1	0	0
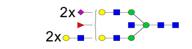		0,758	3423,237442	20,901	7	7	1	2	0
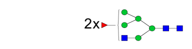		0,662	1850,681381	14,991	5	3	2	0	0
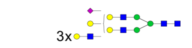		0,584	3310,189763	20,866	9	7	0	1	0
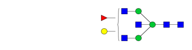		0,565	1948,729394	14,436	4	5	1	0	0
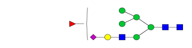		0,524	2157,771712	17,347	6	3	1	1	0
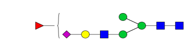		0,514	1833,666065	14,001	4	3	1	1	0
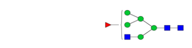		0,500	1704,623472	13,911	5	3	1	0	0
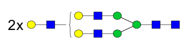		0,498	2491,909328	17,773	7	6	0	0	0
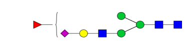		0,469	1833,666065	14,305	4	3	1	1	0
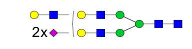		0,418	2708,967965	19,593	6	5	0	2	0
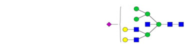		0,407	2579,925372	18,473	7	5	0	1	0
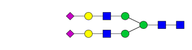		0,382	2343,835769	17,727	5	4	0	2	0
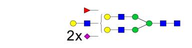		0,342	2855,025873	19,73	6	5	1	2	0
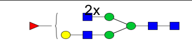		0,315	1745,650021	13,693	4	4	1	0	0
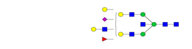		0,302	3091,115476	19,525	8	6	1	1	0
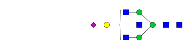		0,295	2093,766901	16,489	4	5	0	1	0
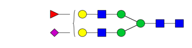		0,289	2198,798261	15,866	5	4	1	1	0
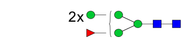		0,270	1501,544099	13,837	5	2	1	0	0
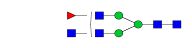		0,267	1786,67657	13,884	3	5	1	0	0
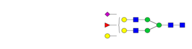		0,260	2360,851053	17,271	6	4	1	1	0
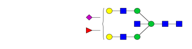		0,249	2401,877634	17,801	5	5	1	1	0
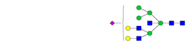		0,243	2579,925372	18,822	7	5	0	1	0
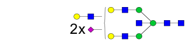		0,238	2912,047337	20,135	6	6	0	2	0
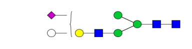		0,216	1849,660949	16,452	5	3	0	1	0
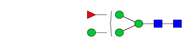		0,213	1339,491276	12,369	4	2	1	0	0
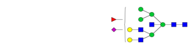		0,208	2725,98328	18,905	7	5	1	1	0
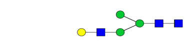		0,174	1396,51274	12,674	4	3	0	0	0
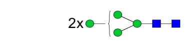		0,154	1355,486191	19,612	5	2	0	0	0
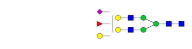		0,146	2360,851053	17,783	6	4	1	1	0
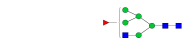		0,143	1704,623472	14,253	5	3	1	0	0
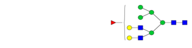		0,134	2231,808491	16,263	7	4	1	0	0
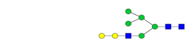		0,129	1882,67121	8,6	7	3	0	0	0
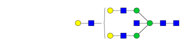		0,122	2329,856504	16,703	6	6	0	0	0
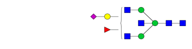		0,122	2239,82481	15,526	4	5	1	1	0
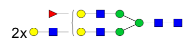		0,115	2637,967236	17,774	7	6	1	0	0

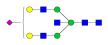	0,106	2255,819725	16,256	5	5	0	1	0
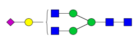	0,102	1890,687529	14,603	4	4	0	1	0
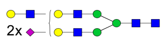	0,094	2708,967965	19,651	6	5	0	2	0
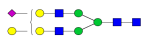	0,090	2214,793145	17,57	6	4	0	1	0
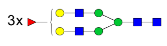	0,084	2199,818662	16,209	5	4	3	0	0
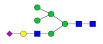	0,070	2011,713772	17,382	6	3	0	1	0
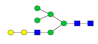	0,069	1882,67121	8,083	7	3	0	0	0
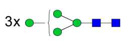	0,068	1517,539014	15,04	6	2	0	0	0
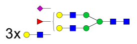	0,066	3294,194849	19,948	8	7	1	1	0
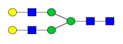	0,064	1761,644936	14,866	5	4	0	0	0
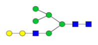	0,062	1882,67121	8,234	7	3	0	0	0
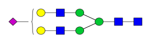	0,047	2052,740321	15,709	5	4	0	1	0
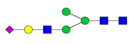	0,043	1687,608156	15,318	4	3	0	1	0
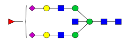	0,039	2692,97305	19,612	5	5	1	2	0
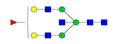	0,031	2110,782217	15,815	5	5	1	0	0
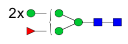	0,025	1501,544099	13,974	5	2	1	0	0
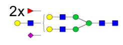	0,024	2709,988366	19,647	6	5	2	1	0
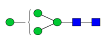	0,021	1193,433367	12,171	4	2	0	0	0
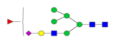	0,019	2157,771712	17,669	6	3	1	1	0
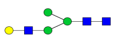	0,018	1396,51274	22,453	4	3	0	0	0
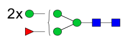	0,017	1501,544099	18,522	5	2	1	0	0
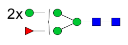	0,015	1501,544099	18,223	5	2	1	0	0
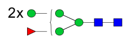	0,015	1501,544099	19,174	5	2	1	0	0
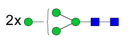	0,014	1355,486191	19,137	5	2	0	0	0
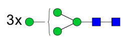	0,011	1517,539014	22,968	6	2	0	0	0
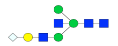	0,011	1906,682412	8,191	4	4	0	0	1
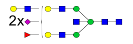	0,010	3058,105246	20,195	6	6	1	2	0
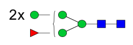	0,008	1501,544099	18,746	5	2	1	0	0
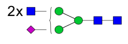	0,007	1728,634705	20,355	3	4	0	1	0
