# Supplementary material for: Glycan remodeled erythrocytes facilitate antigenic characterization of recent A/H3N2 influenza viruses
Source: Nat Commun. 2021 Sep 14;12:5449. doi: 10.1038/s41467-021-25713-1 (PMC8440751; doi:10.1038/s41467-021-25713-1)
Supplement: Supplementary file 6 — Reporting Summary [file 41467_2021_25713_MOESM6_ESM.pdf]

nature research

Corresponding author(s): Robert P. de Vries and Geert-Jan Boons

Last updated by author(s): Dec 18, 2020

Reporting Summary

Nature Research wishes to improve the reproducibility of the work that we publish. This form provides structure for consistency and transparency in reporting. For further information on Nature Research policies, see our Editorial Policies and the Editorial Policy Checklist.

Statistics

For all statistical analyses, confirm that the following items are present in the figure legend, table legend, main text, or Methods section.

n/a	Confirmed	
☐	☒	The exact sample size (n) for each experimental group/condition, given as a discrete number and unit of measurement
☐	☒	A statement on whether measurements were taken from distinct samples or whether the same sample was measured repeatedly
☒	☐	The statistical test(s) used AND whether they are one- or two-sided<br>*Only common tests should be described solely by name; describe more complex techniques in the Methods section.*
☒	☐	A description of all covariates tested
☒	☐	A description of any assumptions or corrections, such as tests of normality and adjustment for multiple comparisons
☐	☒	A full description of the statistical parameters including central tendency (e.g. means) or other basic estimates (e.g. regression coefficient) AND variation (e.g. standard deviation) or associated estimates of uncertainty (e.g. confidence intervals)
☒	☐	For null hypothesis testing, the test statistic (e.g. F, t, r) with confidence intervals, effect sizes, degrees of freedom and P value noted<br>*Give P values as exact values whenever suitable.*
☒	☐	For Bayesian analysis, information on the choice of priors and Markov chain Monte Carlo settings
☒	☐	For hierarchical and complex designs, identification of the appropriate level for tests and full reporting of outcomes
☐	☒	Estimates of effect sizes (e.g. Cohen's d, Pearson's r), indicating how they were calculated

Our web collection on statistics for biologists contains articles on many of the points above.

Software and code

Policy information about availability of computer code

Data collection	VnmrJ 4 and TopSpin 4 were used to collect NMR data. The LC-MS data was collected using a 1260 Infinity liquid chromatography system (Agilent Technologies) coupled to a 6560 IM-QTOF mass spectrometer (Agilent technologies). LC-MS data aquisition was performed using Agilent MassHunter Workstation Data Acquisition B.09 software. Innopsys Mapix 8.1.0 was used to collect glycan microarray data. For the structural studies protein sequences and structures were derived from the 3DPlu database (http://3dflu.cent.uw.edu.pl/index.html) and the GISAID webpage (https://www.gisaid.org). Glycan receptors for the MD simulation were generated using the carbohydrate builder GLYCAM webpage (http://glycam.org). The mutagenesis tool implemented in The PyMOL Molecular Graphics System, Version 2.0 Schrödinger, LLC, was used for the structural model of NL17.
Data analysis	MestReNova 11 was used to analyse NMR data. PNNL prepocessor v2017.11, IM-MS prepocessor and Agilent MassHunter IM-MS browser 8.0 were used to process LC-MS data. Glycan structures were identified from processed MS data using the online Glycomod tool available at https://web.expasy.org/glycomod/. Microsoft Excel (Office2016) was used to process microarray data. The Excel Macro for batch processing was uploaded to https://github.com/enthalpyliu/carbohydrate-microarray-processing. Graphpad Prism 8.3.1 was used to plot bar charts and graphs. Spearmans rank correlation coefficient was calculated using in the build-in function in Graphpad Prism 8.3.1. The MD simulations were performed using the Amber16 Program4 with the protein.ff14SB, the GLYCAM_06j-1 and the water.tip3p force field parameters.

For manuscripts utilizing custom algorithms or software that are central to the research but not yet described in published literature, software must be made available to editors and reviewers. We strongly encourage code deposition in a community repository (e.g. GitHub). See the Nature Research guidelines for submitting code & software for further information.

Data

Policy information about availability of data

All manuscripts must include a data availability statement. This statement should provide the following information, where applicable:

- Accession codes, unique identifiers, or web links for publicly available datasets
- A list of figures that have associated raw data
- A description of any restrictions on data availability

The data supporting the findings in this paper are available in the manuscript and Supporting informtation. A full overview of the identified glycomic structures can be found in the Supplementary Data files S1 and S2. The source data files for Figure 1A and supplementary figures S1,S3 and S8 can be found in Data file S3.

Field-specific reporting

Please select the one below that is the best fit for your research. If you are not sure, read the appropriate sections before making your selection.

[x] Life sciences [ ] Behavioural & social sciences [ ] Ecological, evolutionary & environmental sciences

For a reference copy of the document with all sections, see nature.com/documents/nr-reporting-summary-flat.pdf

Life sciences study design

All studies must disclose on these points even when the disclosure is negative.

Sample size	For HA, HI and FRA assays, viruses and vaccine strains were chosen, representative for distinct evolutionary time points and clades. All HA experiements are performed in three biological replicates. Performing three independent experiments is generally accepted in the field.
Data exclusions	No data were excluded from the analyses
Replication	The HI and FRA assays were performed in biological duplicates. The HA assays were performed in biological triplicates. All replication attempts were successful and produced consistent results. All repetitions performed by other independently were successful and consistent
Randomization	Not relevant for this study, the experimental setup did not allow for randomization.
Blinding	Investigators were blinded while performing the HA, HI and FRA assays.

Reporting for specific materials, systems and methods

We require information from authors about some types of materials, experimental systems and methods used in many studies. Here, indicate whether each material, system or method listed is relevant to your study. If you are not sure if a list item applies to your research, read the appropriate section before selecting a response.

Materials & experimental systems

n/a	Involved in the study	
[ ]	[x]	Antibodies
[ ]	[x]	Eukaryotic cell lines
[x]	[ ]	Palaeontology and archaeology
[ ]	[x]	Animals and other organisms
[x]	[ ]	Human research participants
[x]	[ ]	Clinical data
[x]	[ ]	Dual use research of concern

Methods

n/a	Involved in the study	
[x]	[ ]	ChIP-seq
[x]	[ ]	Flow cytometry
[x]	[ ]	MRI-based neuroimaging

Antibodies

Antibodies used	Ferret antisera were raised in house by intranasal inoculation with influenza A virus and serum collection at 14 days post inoculation. Antibodies used for microarray studies: CR8020 A/H3N2 (PMID: 21737702) stem antibody kindly provided by Dr. Dirk Eggink, secondary Goat anti-human Alex-647 (cat# A21445 Thermo), and streptavidin AlexaFluor-635 (cat# S32364 Thermo)
Validation	Ferret antisera were titrated against homologous and heterologous influenza viruses in-house. The CR8020 A/H3N2 antibody was validated in an ELISA assay against multiple H1 and H3 hemagglutinin proteins produced in-house. Another validation was the use of this antibody against H1 viruses on the array, which the antibody did not recognize.

Eukaryotic cell lines

Policy information about cell lines

Cell line source(s)	American Type Culture Collection (MDCK), Prof. Dr. Kawaoka (hCK) and Prof. Dr. Matrosovich (MDCK-SIAT) PMID: 12857911
Authentication	None of the cell lines used were authenticated.
Mycoplasma contamination	All cell lines tested negative for mycoplasma
Commonly misidentified lines (See ICLAC register)	None of the cell lines used in this study have been identified as commonly misidentified lines

Animals and other organisms

Policy information about studies involving animals; ARRIVE guidelines recommended for reporting animal research

Laboratory animals	Mustela putorius furo, male, 6-12 month old
Wild animals	the study did not involve wild animals
Field-collected samples	the study did not involve samples collected from the field
Ethics oversight	Independent animal experimentation ethical review committee ‘stichting DEC consult’.

Note that full information on the approval of the study protocol must also be provided in the manuscript.
